# Trade-off between critical metal requirement and transportation decarbonization in automotive electrification

**DOI:** 10.1038/s41467-023-37373-4

**Published:** 2023-04-11

**Authors:** Chunbo Zhang, Xiang Zhao, Romain Sacchi, Fengqi You

**Affiliations:** 1 Robert Frederick Smith School of Chemical and Biomolecular Engineering, Cornell University, Ithaca, New York 14853 USA; 2 Systems Engineering, Cornell University, Ithaca, New York 14853 USA; 3 Technology Assessment Group, Laboratory for Energy Systems Analysis, Paul Scherrer Institute, Villigen, Switzerland; 4 Cornell Atkinson Center for Sustainability, Cornell University, Ithaca, New York 14853 USA

**Keywords:** Energy infrastructure, Climate sciences, Energy and society

## Abstract

Automotive electrification holds the promise of mitigating transportation-related greenhouse gas (GHG) emissions, yet at the expense of growing demand for critical metals. Here, we analyze the trade-off between the decarbonization potential of the road transportation sector and its critical metal requirement from the demand-side perspective in 48 major countries committing to decarbonize their road transportation sectors aided by electric vehicles (EVs). Our results demonstrate that deploying EVs with 40–100% penetration by 2050 can increase lithium, nickel, cobalt, and manganese demands by 2909–7513%, 2127–5426%, 1039–2684%, and 1099–2838%, respectively, and grow platinum group metal requirement by 131–179% in the 48 investigated countries, relative to 2020. Higher EV penetration reduces GHG emissions from fuel use regardless of the transportation energy transition, while those from fuel production are more sensitive to energy-sector decarbonization and could reach nearly “net zero” by 2040.

## Abstract (web summary)

This study analyzes the trade-off between the decarbonization potential of the road transportation sector and its critical metal requirements in 48 countries. Our results show that transportation electrification may result in an upsurge in critical metal demand, and decarbonizing fuel production is critical for adequately mitigating greenhouse gas emissions from road transportation.

## Introduction

Climate change has come to the top of the global agenda. The United Nations Climate Change Conference in Glasgow reaffirmed the Paris Agreement’s carbon neutrality goals and called for an urgent phase-down of fossil fuels^1^. The transportation sector can contribute to approximately one-quarter of all energy-induced greenhouse gas (GHG) emissions, and three-quarters of transportation emissions are from road traffic^2^. Therefore, decarbonizing the road transportation sector is crucial. Reducing road traffic activities and improving the efficiency of internal combustion engine vehicles (ICEVs) can cut GHG emissions from combusting fossil fuels. Yet, deep decarbonization of the road transportation sector requires a substantial shift to cleaner fuels^3^. Electricity generated from renewable sources, such as wind, solar, geothermal, tidal, and hydro energy, is seen as an ideal solution to mitigate GHG emissions^4^. Substituting ICEVs with electric vehicles (EVs) powered by renewable electricity can pave the way to achieving transportation decarbonization goals^5^. For instance, European Commission has issued a ban on the sale of new petrol and diesel cars from 2035^6^. In addition to electrification, biofuels—defined as liquid fuels and blending components produced from biomass materials (e.g., bioethanol and biodiesel)^7^—can offer great help to further decarbonization^8^. The current application of biofuels is still hampered by their higher production costs^9^, water footprint^10,11^, and land use change emissions^12^ compared with fossil fuels. More importantly, biofuel production consumes crops and competes with arable land for food production when total global food demands are expected to increase by 35–56% by 2050^13^. With technological advances and production yield gains, the next-generation biofuels made from non-food-based feedstock, such as lignocellulose-based and algae-based biofuels^14^, are expected to be sustainable energy sources^9^. In these contexts, biofuels and electrification effectively decarbonize road transportation.

Adopting EVs is a key to decarbonizing transportation at the expense of tremendous critical metal requirements than their fossil fuel counterparts^15^, exacerbating the supply risks of critical metals. Critical metals can generally be understood as those essential to the functioning and integrity of a wide range of industrial ecosystems, usually with a high economic value but also raising an equally high risk of supply disruption^16^. This study focuses on the critical metals used for the electrification of the transportation sector. Current EVs are exclusively powered by lithium-ion batteries (LIBs)^17^ due to the unmatchable characteristics of their high energy and power density^18^. Lithium nickel cobalt aluminum oxide (NCA), lithium nickel manganese cobalt oxide (NMC), and lithium iron phosphate (LFP) batteries are currently the most widely used EV LIBs^19^, for which lithium, nickel, cobalt, and manganese are needed in the battery cathode production. Other than these four metals, the criticality of platinum group metals (PGMs) has been credited much earlier due to their high rarity^20^. PGMs, such as platinum and palladium, are used in catalytic converters to oxidize carbon monoxide and hydrocarbons in tailpipe exhausts of ICEVs. The current fuel cell electric vehicles (FCEVs) also adopt PGMs to catalyze electrode reactions, increasing the reliance of the transportation sector on the availability of PGMs. The booming EV market, therefore, may lead to the potential supply shortage of critical metals^21^. Hence, investigating the effects of deploying EVs on critical metals requirements and mitigation of transportation GHG emissions is essential. Past studies have explored the GHG emissions mitigation potential and critical material requirements of electrifying transportation in specific countries and regions, including China^22–27^, the USA^26,28–34^, Europe^8,26,34–38^, India^26,39,40^, and the world^41–49^, for various road transportation sectors corresponding to passenger vehicles^8,24–27,38–40,44,45,50^, freight^8,25,31^, light-duty vehicles^8,24,25,27,31,33,39,42,44,45,50^, and heavy-duty vehicles^8,25,39,42^ sectors. Despite the efforts made to project the impacts of EV deployment, several knowledge gaps still remain. First, most studies investigated a specific transportation sector^22,27–29,31–33,36,37,39,41–55^ or a specific category of EVs^27–29,31–33,36,37,39,42–55^. Lacking perspective from the full range of vehicles composing a fleet may lead to incomplete or truncated conclusions. The analysis scope can be broadened by considering the electrification of a broader range of vehicles, including battery electric vehicles (BEVs), plug-in hybrid electric vehicles (PHEVs), hybrid electric vehicles (HEVs), and FCEVs in both light-/heavy-duty and passenger/freight sectors to lay out holistic strategies to mitigate GHG emissions and critical metal use. Second, current studies explored transportation electrification from an integrated global perspective^41,44,46,50,52,56^ or for a specific region^22,24,28,29,32,33,35–37,39,51,53–55,57–60^ without regional analyses to show the spatial and temporal variability of GHG emissions and material requirements associated with electrifying transportation. Therefore, localized projections of transportation electrification in specific areas would help better plan the regional EV adaptation strategies and identify hotspots for mitigating global GHG emissions and critical metal use. Third, existing studies considered the impacts of EV penetration and decarbonization of energy systems separately^31,32,39,49,58,59^ but did not explicitly discuss the synergistic coupling between EV penetration and energy transition in alleviating GHG emissions and shortening the time to reach carbon peak. Finally, relevant studies only discussed the critical metal requirements and GHG mitigation potential of transportation electrification separately^29,32,33,37,39,42–45,48–50,56,58–61^ without identifying potential integrated strategies for GHG emissions mitigation in a resource-efficient manner.

Here, we quantify the spatiotemporal patterns of GHG emissions and critical metals requirement in electrifying the light-/heavy-duty passenger and freight road fleets in multiple regions under different EV penetration and energy transition scenarios, considering a complete range of major EV technologies, including BEVs, PHEVs, HEVs, and FCEVs. Transportation fuels considered in this study include electricity, gasoline, bioethanol, natural gas, diesel, biodiesel, and hydrogen. Forty-eight countries committing to decarbonizing their transportation sector are considered in this study. The investigated areas account for 61% of the global population in 2020^62^, which can shed light on the global trend in electrifying road transportation. The temporal scope ranges from 2010, in which the multi-governmental policy forum Electric Vehicles Initiative was launched to boost the deployment of EVs worldwide^63^, to 2050, when global anthropogenic GHG emissions are expected to reach “net zero”^64^. We quantify the vehicle stocks and flows and the associated requirements of critical metals from the demand-side perspective, including lithium, cobalt, nickel, manganese, and PGMs, for four EV penetration rates (40, 60, 80, and 100%) by referring to the International Energy Agency (IEA)‘s outlook for the future EV market^4,63,65^. We also examine the GHG emissions of road transportation under three energy system transition scenarios derived from the integrated assessment model (IAM) IMAGE 3.2 to limit global temperature rise to 3.5, 2, and 1.5 °C by the end of this century, compared to pre-industrial levels^66^. Compared to the baseline 3.5 °C scenario, the more ambitious 2 °C and 1.5 °C scenarios indicate a higher penetration of renewables in the electricity system, cleaner hydrogen production processes, a higher proportion of biofuels blended in gasoline and diesel, and the more widespread application of carbon capture and storage (CCS) technologies in fuel production. The findings of this study can provide insights into identifying and tackling the latent critical metal supply risks and climate change mitigation potential of alternative fuels in transportation electrification.

## Results

### Future vehicle and battery market

**Fig. 1:**
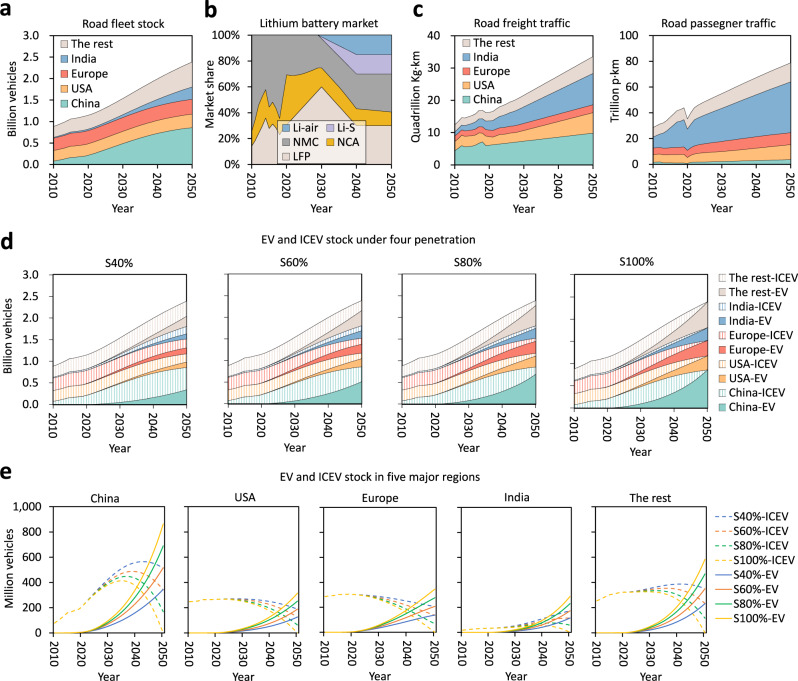
Future markets of road fleet, lithium-ion batteries, and freight and passenger traffic. **a** Road fleet stock of China, the USA, Europe, India, and the rest of the regions. **b** Projection of the future lithium-ion battery market. **c** Road freight (expressed in Kg·km, or one kilogram transported over one kilometer) and passenger (expressed in p·km, or one passenger transported over one kilometer) traffic activities. **d**, **e** Stock of EV and ICEV in five major regions. ICEV internal combustion engine vehicle, EV electric vehicle, NMC lithium nickel manganese cobalt oxide battery, NCA lithium nickel cobalt aluminum oxide battery, LFP lithium iron phosphate battery, Li-S lithium-sulfur battery, Li-air lithium-air battery. HEVs are accounted for as ICEVs.

Information on future automotive markets (2010–2050), the EV battery market, and the transportation service market is collected and modeled to quantify the regionalized use of critical metals and fuels for each vehicle type. Figure 1a shows the total regionalized future vehicle stock. From 2010 to 2050, the total stock of vehicles in the 48 countries investigated is estimated to grow by a factor of 2.7, from 0.88 billion to 2.39 billion. Europe and the USA possessed the most vehicles in 2010, accounting for 32% (0.29 billion) and 28% (0.25 billion) of the total vehicle stock, respectively. Their respective share of vehicle stocks will decrease to 14% and 13% by 2050 due to the rapid expansion of vehicle markets in developing countries. China will surpass other countries’ vehicle market size expansion from 0.08 billion (9%) in 2010 to 0.86 billion (36%) in 2050. India also sees its vehicle ownership increase exponentially from 0.02 billion (2%) in 2010 to 0.29 billion (12%) in 2050. From 2010 to 2050, light-duty passenger and commercial vehicles account for an average of 82% and 13% of total vehicles in the investigated regions, respectively, while heavy-duty passenger and commercial vehicles have a market share of less than 5%^43,67^. The details of the assumptions and sources for the future vehicle market are given in Supplementary Information.

Four EV deployment scenarios are established according to four EV penetration levels: S40%, S60%, S80%, and S100% scenarios to analyze the future global automotive market. They are based on EV penetration scenarios such as Stated Policies, Announced Pledges, BLUE Map, Sustainable Development, and Net Zero Emissions, all devised by the IEA^4,63,65^. The “40%” penetration level in the “S40% scenario” indicates that EV stock in each region will increase to 40% of the total road fleet stock by 2050. Figure 1d, e shows the extrapolated ICEV and EV stock by 2050 for China, the USA, Europe, India, and other regions.

In all four scenarios, the EV stocks are monotonously increasing within all regions. BEVs will play a pivotal role in automobile electrification, accounting for about 60% of the global vehicle stock by 2050. PHEVs will account for 25% of the global EV stock in 2050, and FCEVs are assumed to have a market share of ~15% in 2050 based on IEA’s projection^63^. In addition, the ICEV stock trend differs across regions and scenarios. China’s ICEV stock has grown since 2010 and will peak between 2035 and 2040. The stocks of ICEVs for developed economies will top earlier: 2022–2026 for the USA and 2019–2020 for Europe. The ICEV stock in India will keep rising until 2050 under the S40% and S60% scenarios and peak in 2043 and 2039 under the S80% and S100% scenarios, respectively. Supplementary Figs. S4–S13 show the detailed mode-specific stock of EVs and ICEVs.

LIB technology dominates the traction batteries used to power EVs. As shown in Fig. 1b, NMC/NCA, LFP, and Li-S/Li-air are three typical technologies in the future EV battery market. NMC/NCA battery technologies are the most favorable to EVs. The specific energy of NMC/NCA LIBs has increased from less than 100 Wh/kg in the 1990s to more than 250 Wh/kg currently at the cell level^17^. Because cobalt is not as abundant as nickel, the LIB industry has optimized the composition of NMC111 to produce material-efficient LIB alternatives such as NMC523, NMC622, NMC811, and NMC955. Compared to NMC/NCA batteries, LFP batteries offer a longer life span, greater economic viability, and excellent thermal and electrochemical stability^68,69^. The specific energy of LFP batteries, ~170 Wh/kg at the cell level^70^, is relatively lower than that of NMC/NCA batteries. However, their greater thermal stability allows for the integration of the cells directly in the battery pack (i.e., “cell-to-pack”, or “battery blade”), thereby increasing their specific energy at the battery level to an extent similar to NMC/NCA batteries. Based on the previous study^44^, we assume that the market share of LFP batteries will expand to 60% in 2030, then gradually decrease to 30% in 2040 and remain stable till 2050 (Fig. 1b). Moreover, this study considers two emerging solid-state battery technologies—Li-S and Li-air batteries—that are envisioned as next-generation technologies, outperforming LIBs in specific energy^17^. Solid-state Li-S batteries can achieve a maximum specific energy of 600 Wh/kg at the cell level^17^, leading to a longer EV range, light battery mass, and lower production costs. However, low cycle life, high self-discharge rates, and safety issues are critical technical challenges for sulfur cathodes in Li-S batteries^71^. Li-air batteries with a higher maximum specific energy (i.e., ~800 Wh/kg at the cell level) than the Li–S batteries^17^ still exhibit lower charging life^72^. Based on the forecast of Xu et al.^44^, we assume that technological advancements in solid-state batteries will enable Li-S/air batteries to be commercially available by 2030, and their market share will stabilize at 30% in 2040, as shown in Fig. 1c.

Figure 1c depicts the loads for transportation for freight and passenger. In 2010, China had the largest freight transportation market, accounting for ~35% of the total freight market globally, although this share will drop to 29% by 2050. India’s freight traffic market share will grow rapidly from 10% in 2010 to 29% in 2050. Moreover, Fig. 1c shows that the total freight traffic activities are impeded by the COVID-19-induced blockade in 2020, with passenger traffic proportionally more impacted. China is almost negligible (5%) relative to the total passenger traffic globally because the passenger load from the railway systems is omitted. India has the highest road passenger traffic activity, accounting for 50% in 2050. Passenger traffic in the USA and Europe accounts for 15% and 12% of the global transportation load, respectively.

### Requirement of critical metals

**Fig. 2:**
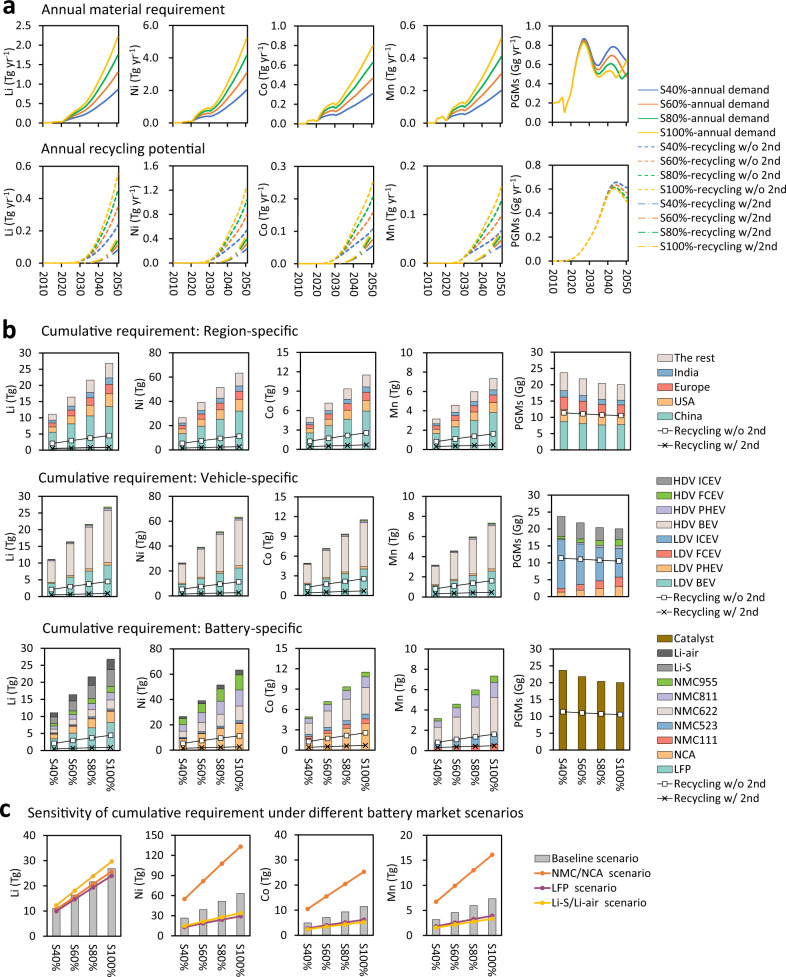
Annual and cumulative critical metal requirements and the closed-loop recycling potential of critical metals. **a** Annual demand and recycling potential with or without a second use. **b** Region-specific/vehicle-specific/battery-specific cumulative (from 2010 to 2050) demand for critical metals and the cumulative potential secondary production from recycling. **c** Sensitivity of cumulative requirement under different battery scenarios. NMC/NCA scenario illustrates that the market share of NMC/NCA will increase to 100% by 2050. “Recycling w/o 2nd” indicates retired batteries that are directly recycled without a second life as energy storage systems (ESSs). “Recycling w/2nd” denotes retired batteries reused as ESSs before recycling. LDV light-duty vehicle, HDV heavy-duty vehicle, BEV battery electric vehicle, PHEV plug-in hybrid electric vehicle, FCEV fuel cell electric vehicle, ICEV internal combustion engine vehicle, LFP lithium iron phosphate battery, NCA lithium nickel cobalt aluminum oxide battery, NMC lithium nickel cobalt manganese oxide battery, Li-S lithium-sulfur battery, Li-air lithium-air battery.

Based on the simulated future market trends, we estimate the future requirement for critical metals for the decarbonization transition of the road transportation sector. As observed in Fig. 2a, if deploying EVs with 40–100% penetration by 2050, the demand for critical battery metals, including lithium, nickel, cobalt, and manganese, can be continuously increased since 2020 by 2909 (S40% scenario)–7513% (S100% scenario), 2127–5426%, and 1039–2684%, and 1099–2838%, respectively, because of the higher EV penetration levels that require battery manufacturing. The annual demand for lithium increases monotonically from 2010 to 2050: 0.7–900 Gg in the S40% scenario and 0.7–2200 Gg in the S100% scenario. The demand for nickel tops that of other critical metals, ranging from 2.0 Tg in the S40% scenario to 5.2 Tg in the S100% scenario in 2050. The yearly demand for cobalt and manganese is in the same order of magnitude in 2050, at around 0.3–0.8 Tg and 0.2–0.5 Tg, respectively. The demand for PGMs, which fluctuates between 0.1 and 0.9 Gg over the 2010–2050 period, is significantly lower than for the other four battery metals. The demand for PGMs used in PHEVs and FCEVs increase to 0.3–0.7 Gg by 2050, while the need for PGMs in ICEVs peaks in 2026 at 0.7–0.8 Gg (see Supplementary Fig. S15). In the S40% case, PGMs used for ICEVs still account for 58% of the total demand for PGM in 2050. However, in the S100% scenario, ICEVs cease to be sold in 2045, and from then on, PGMs are used exclusively for PHEVs and FCEVs only.

**Fig. 3:**
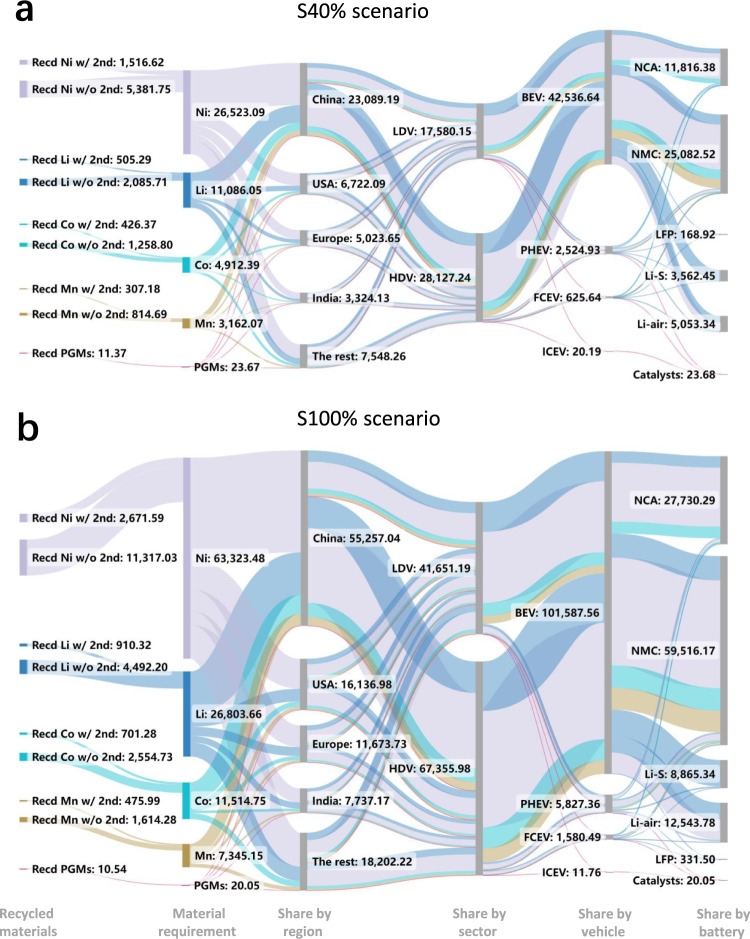
Sankey diagrams for cumulative critical metal requirements (Gg) under the 40 and 100% electric vehicle penetration scenarios. **a** Cumulative critical metal requirements (from 2010 to 2050) under the S40% scenario. **b** Cumulative critical metal requirements under the S100% scenario. Note: The nodes of recycling materials show the cumulative recycling potential of critical metals from either recycling with or without a second life. “Recd Li w/o 2nd” indicates the cumulative recycling potential of lithium without a second use. “Recd Li w/2nd” denotes the cumulative recycling potential of lithium after the second use. PGM platinum group metal, LDV light-duty vehicle, HDV heavy-duty vehicle, BEV battery electric vehicle, PHEV plug-in hybrid electric vehicle, FCEV fuel cell electric vehicle, ICEV internal combustion engine vehicle. LFP Lithium iron phosphate battery, NCA lithium nickel cobalt aluminum oxide battery, NMC lithium nickel cobalt manganese battery, Li-S lithium-sulfur battery, Li-air lithium-air battery.

The cumulative demand for critical metals from 2010 to 2050 in S40% and S100% scenarios are depicted as Sankey diagrams in Fig. 3 (see Supplementary Fig. S14 for S60% and S80% scenarios). A higher EV penetration enhances the cumulative requirements for the four battery-related metals but reduces the need for PGMs. The contribution analysis of cumulative requirements is further shown in Fig. 2b. The region-wise contribution analysis illustrates that China is the world’s largest consumer of critical metals, accounting for ~50% of the cumulative demand for lithium, nickel, cobalt, and manganese and 40% of the cumulative demand for PGMs. Vehicle-wise breakdown of cumulative requirements indicates that over 90% of the total battery metal demand is for BEVs: 55–59% for heavy-duty and 29–38% for light-duty BEVs. PGMs show 59–85% of their total demand for ICEVs, with 43–60% for light-duty ICEVs and 16–24% for heavy-duty ICEVs. In the S40% scenario, PHEVs and FCEVs consume only 15% of the cumulative PGM demand, which rises to 41% in the S100% case. It is also noted that transportation electrification can slightly reduce the cumulative requirement of PGMs by 2050, but the annual demand of PGMs in the S100% scenario is 1.5 Gg higher than that in the S40% scenario in 2050. The battery-wise contribution analysis shows that ~40% of the lithium is used to manufacture NMC/NCA batteries, and around 30% of that is used for Li-S/Li-air and LFP batteries, respectively. It is also found that nickel, cobalt, and manganese are used almost exclusively for producing NMC/NCA batteries. We further evaluate the sensitivity of the assumptions on future battery markets to obtain the upper and lower bounds of the critical metal requirements. Based on the baseline battery market scenario (Fig. 1b), we examined three additional extreme battery market scenarios, in which the market shares of (1) NMC/NCA, (2) LFP, and (3) Li-S/Li-air battery technologies will gradually increase to 100% by 2050, respectively. The results of the sensitivity analysis in Fig. 2c show that the cumulative requirement for lithium remains relatively stable across the three battery scenarios, while that of cobalt, nickel, and manganese could be halved in the Li-S/Li-air and LFP scenarios and may double if NMC/NCA battery technology dominates the future battery market.

We then compare the cumulative demand for the five critical metals with their proven global resources and reserves in 2020 to reveal potential supply risks. Resources for all five metals are sufficient to produce automotive batteries and catalysts. Regarding reserves of critical metals, manganese supply is sufficient regardless of the EV penetration level, as global manganese reserves in 2020 were above 1300 Tg and 200 Tg for EV batteries^73^, which far exceeds the cumulative demand for manganese in 2050 (3–7 Tg). PGM reserves for the automotive industry in 2020 are around 27 Gg, 90% of which originates from South Africa^73^. By 2050, PGM reserves can meet the cumulative demand, from 20 Gg in the S100% scenario to 24 Gg in the S40% scenario. Global proven lithium reserves in 2020 are around 21 Tg, 44% and 22% of which are in Chile and Australia, respectively. It is assumed that 74% of these, or 16 Tg, are available for batteries. The cumulative demand for lithium ranges from 11–27 Tg, determined by the level of EV penetration. Therefore, lithium supplies may only fall short under the high EV penetration rates (80–100%), depending on what share of lithium reserves are assumed to be available for batteries. However, with more and more lithium deposits being discovered, and explorations and new mining technologies turning resources into reserves, available lithium reserves are expected to increase and close this gap. Based on the share of reserves in 2020 being available for battery production (10 Tg for nickel and 3 Tg for cobalt^73^), nickel and cobalt may fail to meet the cumulative demand by 2050 of 27–63 Tg and 5–12 Tg, respectively^73^. If a larger share or even all the 2020 reserves of 94 Tg for nickel and 7 Tg for cobalt would be available for battery production, there would be no supply risk for nickel. For cobalt, this gap could be closed by a stronger shift to low-cobalt or no cobalt containing battery chemistries. The Democratic Republic of the Congo and Australia have the world’s largest cobalt reserves of ~3.6 Tg and 1.4 Tg^73^, respectively. The three main nickel reserves are located in Indonesia (21 Tg), Australia (20 Tg), and Brazil (16 Tg)^73^. Interestingly, the extraction and processing of critical metals are currently centralized in a few developing and politically unstable countries. It is worth noting that reserves of the five critical metals have remained relatively stable over the last two decades^73^, while reserves may undergo major volatilities in the future based on new geological discoveries, technological advancements, etc.

Recycling discarded batteries allows critical metal recovery that partially substitutes raw resource extraction. Figure 2a shows that this substitution rate is less than 1% in 2020, but will increase to 24–35% by 2050. All EV LIBs will be retired when reaching 80% of their initial energy storage capacity and could be reused for a second life as ESSs after automotive use to reduce peak power consumption from the electricity grid^74^. Reusing discarded EV batteries as ESSs can delay the recycled content rate in batteries by 8–18% in 2050. However, it displaces the need for primary metals required for the manufacture of ESSs—although this has been left out of the scope of this study. Recycling PGMs can satisfy 77–121% of the demand in 2050. Between 2010 and 2050, the accumulated amount of recovered critical metals from discarded batteries can offset the need to extract metallic ores by 17–25%, further decreasing to 3–10% of all reused batteries. Moreover, recycling PGMs can offset natural resource extraction by 47–53% due to the earlier deployment of ICEVs.

### Road transportation GHG emissions

**Fig. 4:**
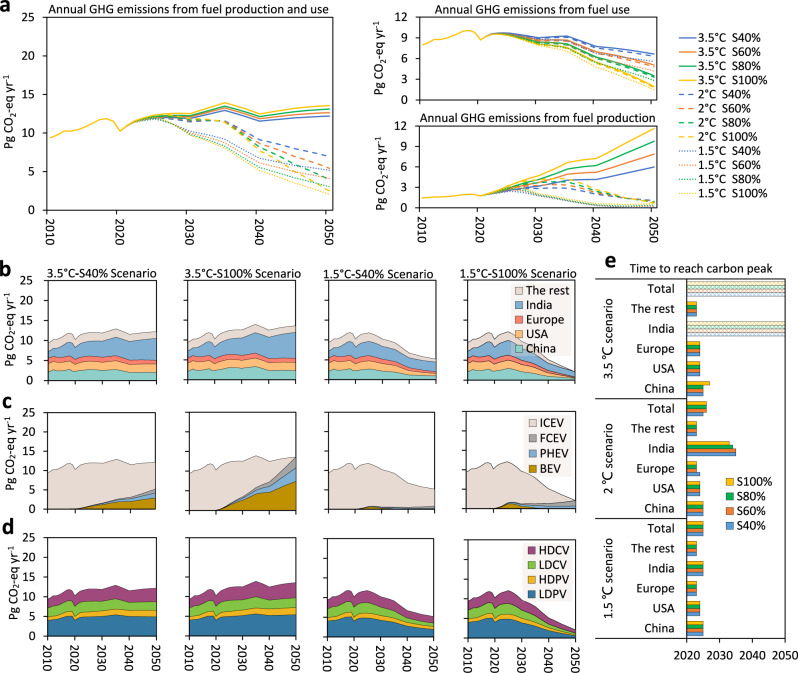
Comparison of annual road transportation fuel greenhouse gas emissions. **a** Annual greenhouse gas (GHG) emissions (Pg CO_2_-eq yr^−1^) from fuel production and use in the 48 counties across four EV penetration scenarios (40–100%) under three energy transition scenarios (1.5–3.5 °C). The results for 2010–2020 are historical statistics; the results for 2021–2050 are projected. **b** Country-wise breakdown of annual GHG emissions. **c** Powertrain-wise breakdown of annual GHG emissions. **d** Sector-wise breakdown of annual GHG emissions. Results for the rest scenarios are given in the SI. **e** Emission peak time in different areas under the 3.5, 2, and 1.5 °C scenarios. The dot-pattern bars in panel (**e**) indicate cases where carbon neutrality by 2050 cannot be achieved. LDPV light-duty passenger vehicle, HDPV heavy-duty passenger vehicle, LDCV light-duty commercial vehicle, HDCV heavy-duty commercial vehicle, BEV battery electric vehicle, PHEV plug-in hybrid electric vehicle, FCEV fuel cell electric vehicle, ICEV internal combustion engine vehicle.

We consider four EV penetration scenarios with three energy transition pathways (1.5–3.5 °C global warming limit conditions) to assess the GHG emissions from fuel for road transportation. Figure 4a (left) shows the global GHG emissions from transportation from 2010 to 2050. The 3.5 °C scenario presents a moderate growth in GHG emissions, whereas the 2 °C and 1.5 °C scenarios witness a sharply declining tendency around 2025 and 2035, respectively. In the 3.5 °C scenario, an increment in EV penetration can lead to more GHG emissions, rising from 10.2 Pg CO_2_-eq in 2020 to 12.2 Pg CO_2_-eq in the S40% scenario and 13.6 Pg CO_2_-eq in the S100% scenario in 2050. In the 2 °C and 1.5 °C scenarios, a higher EV penetration reduces GHG emissions. Figure 4a (right) shows the split of these emissions between fuel use and production. Both electrification and energy transition can cut GHG emissions from fuel use, but electrification dominates in emission mitigation from fuel combustion. In the 3.5 °C-S40% scenario, GHG emissions from fuel use in 2050 could be reduced by 23% from their 2020 level. For the most climate-ambitious 1.5 °C energy transition scenario, where the penetration remains at 40%, a 35% reduction in GHG emissions from fuel use could be achieved. If penetration increases to 100%, even in the mildest 3.5 °C scenario, the GHG emissions from fuel use can be reduced by 77%. GHG emissions from fuel production are more sensitive to energy transition than EV penetration. As seen in Fig. 4a (right), the rise of GHG emissions from fuel production continues throughout the period in the 3.5 °C scenario. These GHG emissions are expected to reach almost zero in 2050 in the 2 °C and 1.5 °C scenarios.

Figure 4b–d depict the contribution of annual GHG emissions from fuel production and use in different regions. The annual GHG emissions in the 3.5 °C scenario increase with age and cannot peak by 2050, while overall GHG emissions in the 2 °C and 1.5 °C scenarios peak in 2025–2026. It is clear from Fig. 4b that India and China are the two biggest GHG emitters. The periods to reach the peak in carbon emissions in each region in Fig. 4b are further summarized in Fig. 4e. The carbon peak time in China, the USA, Europe, and the rest of the regions will stabilize between 2023 and 2027, regardless of EV penetration and energy transition. In contrast, the time of reaching carbon peak in India is more sensitive to energy transition than to EV penetration level. In the 3.5 °C scenario, GHG emissions in India cannot peak by 2050 at four EV penetration levels but are expected to peak in 2033–2035 in the 2 °C scenario and in 2025 in the 1.5 °C scenario. From the perspective of emissions from different vehicles (Fig. 4c), we can see that GHG emissions in the 3.5 °C scenario cannot be reduced even if EV penetration reaches 100% by 2050, while the GHG emissions from EVs are almost negligible in the 1.5 °C scenario. Figure 4d shows that the GHG emissions from freight and passenger transportation are almost split equally.

**Fig. 5:**
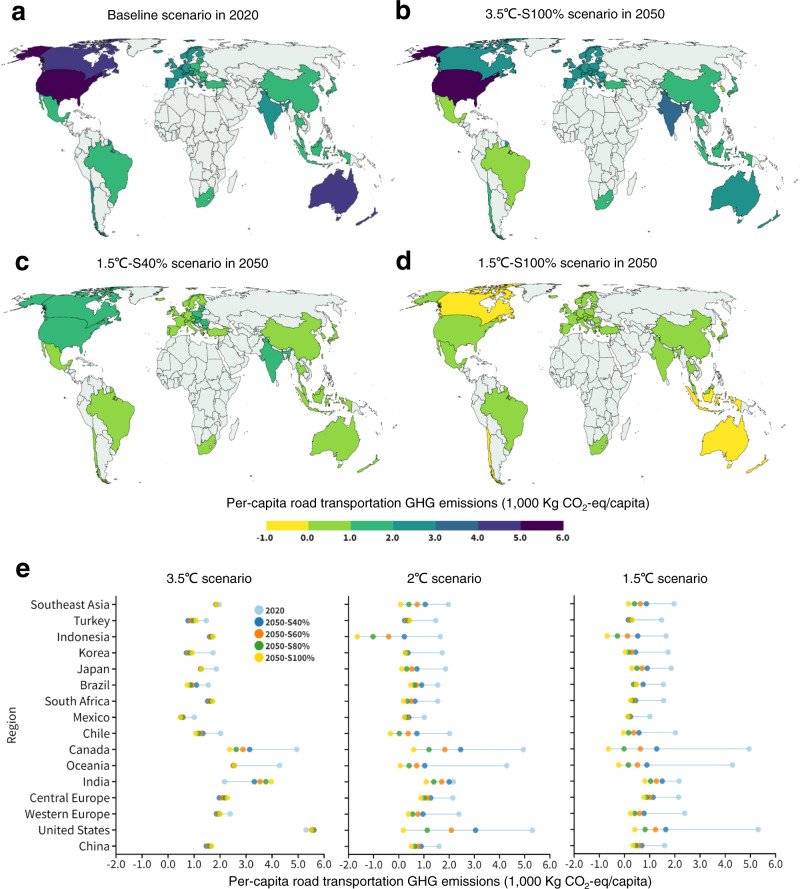
Per-capita greenhouse gas (GHG) emissions from road transportation in 16 regions. **a**–**d** Per-capita GHG emissions from road transportation under the **a** baseline scenario in 2020, **b** 3.5 °C-S100% scenario in 2050, **c** 1.5 °C-S40% scenario in 2050, and **d** 1.5 °C-S100% scenario in 2050. **e** Per-capita GHG emissions from road transportation in 16 regions across the EV penetration and energy transition scenarios.

Figure 5 shows the per-capita GHG emissions in the 16 investigated regions across different EV penetration and energy transition scenarios. We compared the baseline scenario in 2020 (Fig. 5a) with three extreme scenarios in 2050—3.5 °C-S100% (Fig. 5b), 1.5 °C-S40% (Fig. 5c), and 1.5 °C-S100% (Fig. 5d). In 2020 (Fig. 5a), the per-capita GHG emissions in the USA, Canada, Australia, and New Zealand exceeded 4000 Kg, mainly due to high annual per-capita traffic volumes, while that of the rest region ranged from 1000 to 3000 Kg. Under the 3.5 °C-S100% scenario (Fig. 5b), the USA still remains above 5000 Kg CO_2_-eq in 2050, and the per-capita GHG emissions in rest regions slightly decreased because of higher EV penetration, ranging from 0 to 4000 Kg CO_2_-eq. Yet, per-capita GHG emissions in India rise from 2170 in 2020 to 3970 Kg CO_2_-eq in the 3.5 °C-S100% scenario in 2050 due to the use of coal-fired electricity for EVs. Under the 1.5 °C-S40% scenario (Fig. 5c), per-capita GHG emissions in all regions are considerably reduced to 0–2000 Kg CO_2_-eq. Under the 1.5 °C-S100% scenario (Fig. 5d), per-capita GHG emissions in each region decrease below 1000 Kg CO_2_-eq, and Australia, New Zealand, Indonesia, Chile, and Canada drop to negative emissions, consequent from the application of biofuels and the implementation of CCS technologies. The detailed per-capita GHG emissions are depicted in Fig. 5e.

**Fig. 6:**
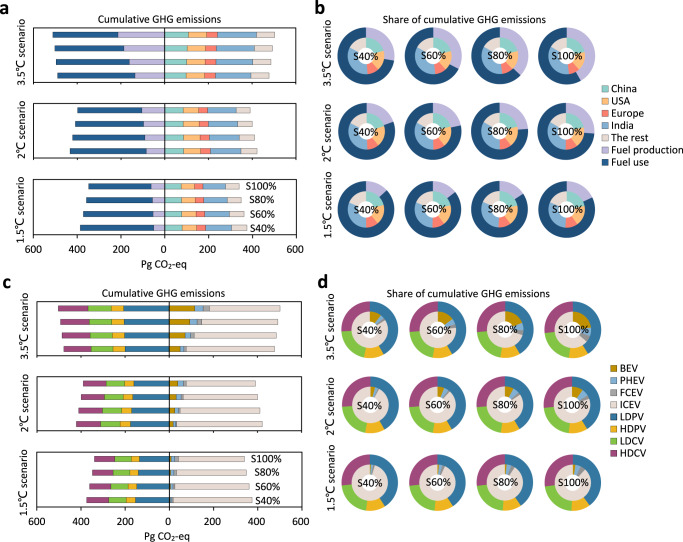
Comparison of cumulative road transportation fuel greenhouse gas (GHG) emissions. **a**, **b** Cumulative GHG emissions (Pg CO_2_-eq yr^−1^) breakdown and share based on fuel production and use and countries investigated. **c**, **d** Cumulative GHG emissions (Pg CO_2_-eq yr^−1^) breakdown and share based on fuel powertrain of vehicle and transportation sector. LDPV light-duty passenger vehicle, HDPV heavy-duty passenger vehicle, LDCV light-duty commercial vehicle, HDCV heavy-duty commercial vehicle, BEV battery electric vehicle, PHEV plug-in hybrid electric vehicle, FCEV fuel cell electric vehicle, ICEV internal combustion engine vehicle.

Figure 6 shows the cumulative GHG emissions (Fig. 6a, c) and the shares (Fig. 6b, d) from the perspective of fuel production and use, regions, vehicles, and transportation sectors. In the 3.5 °C scenario, the cumulative GHG emissions increase with EV penetration levels, from 478 Pg CO_2_-eq in the S40% scenario to 503 Pg CO_2_-eq in the S100% scenario. Facilitating the EV penetration can reduce the cumulative GHG emissions from 422 Pg CO_2_-eq to 391 Pg CO_2_-eq in the 2 °C scenario and from 374 to 339 Pg CO_2_-eq in the 1.5 °C scenario. From the perspective of fuel production and use, the cumulative GHG emissions from fuel use account for a much larger share, from 298–354 Pg CO_2_-eq (82–86% of the total cumulative GHG emissions) in the 3.5 °C scenario to 285–335 Pg CO_2_-eq (58–72%) in the 1.5 °C scenario. Whereas GHG emissions from fuel production are more sensitive to the energy transition, decreasing from 135–213 Pg CO_2_-eq in the 3.5 °C scenario to 51–62 Pg CO_2_-eq in the 1.5 °C scenario. Similarly, the cumulative GHG emissions from EVs are more sensitive to energy transitions, accounting for 16–36% of the total cumulative GHG emissions in the 3.5 °C scenario and dropping to 5–12% in the 1.5 °C scenario. From the perspectives of regions and transportation sectors, the contribution for each fraction of cumulative GHG emissions remains relatively stable regardless of energy transition and EV penetration.

## Discussion

Electrifying transportation and shifting toward a renewable-based energy supply allows road transportation to reach a deep-decarbonization level. Cumulative GHG emissions from road transportation in the investigated regions can be reduced by 12–33% compared with the baseline 3.5 °C scenario by 2050. Higher EV penetration leads to lower global GHG emissions from fuel use regardless of energy transition scenarios. The GHG emissions from fuel production will continue to increase through 2050 and grow with higher EV penetration in the 3.5 °C scenario but could reach nearly zero around 2040 in the 1.5 °C scenario. The period of 2010–2050 was selected as 2050 is a critical time point for most countries to achieve carbon neutrality. This study shows that no country is expected to achieve zero emissions by 2050 in the road transportation sector because GHG emissions from fuel use cannot be completely decarbonized in every region, and PHEVs still emit GHGs due to the combustion of fossil fuels. Biofuels are indispensable in providing an immediate solution to GHG emissions mitigation, especially before electricity and hydrogen production are decarbonized and fossil fuel-based vehicles are phased out. India is identified as the largest transportation GHG emitter (30–36%) among all surveyed regions; deploying EVs in India would not achieve a carbon peak under the 3.5 scenario by 2050.

Other studies support the findings of this study to a certain extent^27,39,49,75–78^. Fuel-related emissions contribute significantly to the life-cycle carbon footprints of FCEVs and BEVs, and cleaner production of hydrogen or electricity can lead to much less GHG emissions^77^. At a global scale, even if electrification is not matched by power-sector decarbonization, using EVs has average lower GHG emissions than ICEVs in most individual countries in 2015, as suggested by Knobloch et al.^49^. BEVs, for instance, have lower life-cycle GHG emissions than ICEVs in most European countries, as illustrated by Sacchi et al.^75^. For the private transportation sector in China, transportation electrification and energy system decarbonization can reduce the GHG emissions from by 28% in 2050^27^. While adopting BEVs in a region mainly powered by fossil-based electricity may not immediately reduce GHG emissions^76^. For example, BEVs in some parts of India^39^ can pose more GHGs than conventional compressed natural gas and diesel vehicles in 2030. In some regions of the USA, operating BEVs and PHEVs can result in higher GHG emissions than HEVs^78^.

The potential trade-off behind electrification is critical, as investing in large amounts of critical metals might not immediately reduce GHG emissions. Our simulations suggest that deploying EVs increases the cumulative demand for critical metals between 2020 and 2050 in the regions surveyed by factors of 29–75 for lithium, 21–54 for nickel, 10–26 for cobalt, 11–28 for manganese, and 1.3–1.8 for PGMs. The future demand for critical battery metals far exceeds the 2020 production capacity, as evidenced in global-level studies^42–44^. Other studies also show that monotonic growth in global demand for critical metals to 2050 is the most prevalent trend, mainly driven by the EV market penetration and battery technology development^79^. Another considerable challenge is that the current critical metals are centralized in a few politically unstable countries such as Chile, Congo, Indonesia, Brazil, Argentina, and South Africa, according to the World Bank^80^. The unstable supplies of critical metals can exacerbate supply risks under surging demand. According to the contribution analysis, China’s EV deployment accounts for more than half of the total critical metal supply in the investigated areas. Around 93% of battery-related critical metals are used in BEVs, while more than half of PGMs (59–85%) are used for ICEVs worldwide. It is also worth noting that we should be cautious about the electrification of the heavy-duty sector, especially heavy-duty BEVs. Although HDVs only account for 4–11% of the total road fleet in each country, battery-related critical metals used in HDVs account for 62% of the total critical metal demand. Regarding battery technics, if NMC/NCA batteries dominate the future battery market, the demand for cobalt, nickel, and manganese may double. The LFP and Li-S/air batteries are promising alternatives to eliminate or remarkably reduce the use of cobalt, nickel, and manganese while keeping the lithium demand relatively stable. On the other hand, the supply of secondary feedstock from recycling EV batteries is increasingly crucial in reducing the primary production of all five critical metals by less than 1% in 2020 to at least 24% in 2050. However, reusing EV batteries as ESSs can reduce the displacement of primary production to 8–18% in 2050 due to delayed recycling. Note that in a broader system context, second use still helps alleviate that same need in other sectors, but our transportation sector-based material accounting system treats open-loop recovery as a “loss”.

Policies are needed to secure the supply of critical metals. First, prioritizing alternative designs for cathodes/anodes and fuel cell systems is essential to reduce the reliance on primary critical metals. In addition to the metals’ high abundance in the crust, alternative cathode designs could also offer improved specific energy and operational safety^79^. Our sensitivity analysis indicates that LFP and Li-S/air batteries could be adopted to reduce the use of cobalt, nickel, and manganese compared with NMC/NCA batteries. Other batteries with manganese-rich cathodes, such as lithium manganese nickel oxide batteries and lithium manganese iron phosphate batteries, use a higher share of abundant metal manganese and can also serve as a solution to reduce the reliance on cobalt and nickel^79^. Post-LIB technologies such as sodium-ion and zinc-ion batteries are sustainable alternatives, enabling EVs to decouple from using lithium^81^. Similarly, the advancements in metal-organic framework-based catalysts can serve as a potential replacement for PGM in catalyzing the oxygen reduction reaction^82^. Second, a circular economy is also indispensable to secondary supply and even achieve a closed-loop supply chain in the future. Strategies should be considered to promote the recycling efficiency and recovery rate of EoL batteries at a proper pace. Investing in recycling infrastructure too early can lead to a waste of resources, as illustrated by our findings that waste battery metal flows will start booming around 2030. Reusing waste batteries for a second life can delay the emergence of the waste streams until 2035–2040. Therefore, long-term recycling plans need to be adopted to avoid oversupplies of secondary materials or idle infrastructure via strategically balancing the share of recycling/reuse with the capacity of infrastructure. Regarding recycling technologies, direct cathode reuse is considered a more advantageous route and should be boosted to improve recovery efficiency^74^. Standardizations and regulations on battery design of battery chemistries, types and sizes, labeling of cells, and pretreatment operations, including selective collection and sorting, should be established to ensure high-quality recovery^79^. For the waste batteries, we only consider the recyclability of EoL battery flows, while scraps in battery production also need to be considered as they are estimated to be over 900 Gg by 2030^83^. Therefore, scraps from battery production could be the main feed stream for battery recycling plants in the near future^84^. Recycling and re-manufacturing scraps from the upstream could also be important to reduce the demand for critical metals at the early stage. Third, since the production and demand of critical metals remain timely dynamic, further analysis of the supply-side resilience and foresight for demand are needed to provide more information on the overall supply-demand balances for critical metals. Such information can facilitate the formulation of specific energy and resource security strategies in managing critical metals with the highest supply risk for both governments and industries. Finally, strategic actions are also required to actively attract and provide support for investments and cooperation in strengthening the critical metals supply chain both at home and abroad. Countries with major EV markets (e.g., China, the United States, and India) should enhance domestic supply by exploring new deposits and raising the production capacity based on the given supply-demand information. Those countries should also seek opportunities to strengthen and diversify their trading networks with third-world countries with abundant metallic resources (e.g., Congo and South Africa) via resource diplomacy and technology sharing. This may include collaboratively discovering and exploiting new reserves, establishing research and innovation partnerships in metallurgy, material engineering, and geomatics to raise production and mining efficiency and reduce scrap rate, and investing in workforce training to advance their technical capabilities and skills related to processing, refining, and recycling of critical metals.

Regarding climate mitigation policies, decarbonization targets for road transportation should be coupled with EV deployment, the timing of carbon peak and neutrality, and accurate emission budgets. Higher EV penetration rates, earlier carbon peak and neutralization times, and lower GHG emissions may conflict. Therefore, climate targets that coordinate the different factors mentioned above can better facilitate the carbon-neutral transition of the transportation sector. Furthermore, biofuels can be considered a complementary strategy at the early stage of electrification. With embodied greenhouse gas emissions from biofuel production lower than in other regions, Turkey and South Korea are expected to expand the application of biofuels. In developing regions, such as India and South Africa, where grids cannot be fully decarbonized and hydrogen supply networks cannot be established in the short term, the potential adoption of biofuels and PHEVs should be considered as transition options for smoother electrification. A strong technological development trajectory for next-generation biofuels is essential for the introduction of electrification. Whereas widespread use of biofuels could also give rise to the lock-in of incumbent ICEV technologies, thus hindering further development of electrification^85^. To address this problem, various governmental efforts are needed. The government should have a clear phased blueprint for biofuels in future electrification. Regulations and legislations would create abundant niche markets so that some self-reinforcing entry and exit mechanisms for biofuel and EVs would become possible. In particular, coercive policies are crucial to escape lock-in. For instance, terminating sales of ICEVs can completely remove the path dependence on biofuels, such as Europe Union agreeing to end ICEV sales by 2035^86^. Government should also endorse technological expectations of greater safety in EV batteries and assurances that the electrification will benefit in terms of energy efficiency and cost effectiveness^87^. In this way, biofuels could emerge as a viable complement to electrification rather than a substitute. Last but not least, integrated strategies should be set for both GHG mitigation and critical metal use reduction, especially in the heavy-duty sector. Heavy-duty BEVs require more than half of the battery-related critical metals due to the requirement for high battery capacity and necessary battery replacement. Deploying alternative EVs, such as FCEVs and PHEVs, and biofuels for the heavy-duty sector can also achieve significant savings in the use of critical battery metals. On the other hand, regional EV deployment strategies must follow local decarbonization plans for local power grids, biofuel supply chains, and hydrogen production networks to reduce fuel-related GHG emissions.

Several limitations on the simplifications and assumptions exist in our methods. First, our study investigated the critical metals requirement for road transportation and fuel-related GHG emissions in 48 countries from 2010 to 2050. We aggregated the 48 countries into five major regions, which reduces the spatial resolution to some degree and might result in a biased global view. Further research could simulate the remaining variation within larger simulated world regions such as China and the USA, or perform case studies at a global level. These potential studies could also analyze the optimal EV penetration strategies combining the adoption of BEVs, PHEVs, and FCEVs for GHG emissions mitigation and critical metal requirement, or the spatial characteristics of environmental impacts of integrating EV batteries into the electricity grid as ESSs. Moreover, given the potential for hydrogen to play a significant role in decarbonizing the transportation sector, we anticipate a gradual increase in hydrogen production through steam methane reforming and coal gasification with CCS by 2050. However, it is important to note that the true impact of the widespread adoption of FCEVs will depend on the availability of green hydrogen sources, such as electrolysis and biomass, which will be evaluated as more ambitious energy transition scenarios become available in the IMAGE model. Then, instead of specifying the needs of each PGM, we model them as an aggregated group. Assessing the demand of each PGM individually can provide more comprehensive insights into sustainable resource management for deploying FCEVs. Additionally, we only considered the fuel emissions to specifically investigate the effect of alternative fuels on reducing road transportation GHG emissions. However, LIB and fuel cell system production^77,78^ can also drive EVs to higher GHG emissions than ICEVs. Investigating the complete life-cycle environmental impacts of the upstream processes, such as battery and fuel cell production, can provide more comprehensive insights into the decarbonization transition of the road transportation sector. Also, the choice of battery market modeling can significantly influence the critical metal requirements. Our study considered the three major battery options for EVs: NCA/NMC, LFP, and Li-S/air batteries. Despite the sensitivity analysis, advances in battery technologies can completely upend future battery market modeling assumptions. The critical metal requirement should be reinvestigated when the developmental trajectory of battery technologies becomes more predictable. Finally, like most studies, we applied a demand-based approach to account for the use of critical metals, whereas a trade-oriented or supply-based analysis can provide additional insights into global critical metals management.

## Methods

### Overview of the methodologies

The study uses a demand-based accounting approach to assess material requirements and GHG emissions leading to the decarbonization of the road transportation sector. As such, we consider the critical metal requirements and GHG emissions based on expected domestic end-use transportation services provided within a region. Therefore, the supply and trade of materials across countries are not considered in the material accounting system. This study considers the electrification of transportation in 48 countries, which are aggregated into 16 world regions based on the territorial division in the IAM IMAGE 3.2^66^. The transportation and fuel market shares are modeled individually for each of the 16 regions. The results for critical metal requirements and GHG emissions for the 16 regions are then aggregated into five major economic entities: China, the USA, Europe, India, and the remaining regions. The time frame of this study includes a retrospective period from 2010 to 2020 and a look-ahead period from 2021 to 2050. Regarding the scope of material requirements, we account for the direct critical metallic material requirements for LIBs and catalytic converters, including lithium, cobalt, nickel, and manganese used for EV LIBs, and PGMs (platinum, palladium, and rhodium) used to produce catalytic converters in FCEVs, PHEVs, and ICEVs. Material requirements for lead-acid batteries are not considered. The capacity of LIBs in HEVs and light-duty FCEVs is negligible, around 2 kWh^88^, thus not considered either. The key assumptions and parameters are summarized in Supplementary Table S1 in the Supplementary Information.

The methodological framework of this study comprises two modules: dynamic material flow analysis (dMFA) and life-cycle-based carbon footprint analysis, as shown in Supplementary Fig. S1. A dMFA has been proven to be a useful tool to project the metabolism of stocks and flows in the anthroposphere^89^. We modify the stock-driven dMFA developed by Müller^90^ to simulate the dynamics of vehicle stocks and flows. The dMFA model in this study consists of three layers. The vehicle layer simulates the gross vehicle stocks and flows in each region up to 2050. We consider four EV penetration scenarios, in which the market stock share of EVs reaches 40–100% by 2050. The vehicle stock determines the battery stock in the second battery layer, which further determines the battery inflow and end-of-life (EoL) outflows. The battery flows then determine the critical metal requirements and recycling potential at the material layer. Two EoL treatment approaches are modeled to evaluate how retired battery cells with and without second use could displace the production of primary critical metals.

GHG emissions from road transportation are estimated based on the IPCC transportation emissions framework^91^. Therefore, key parameters of transportation activities, transportation market, and fuel emission intensities of passenger and freight transportation are modeled to calculate the transportation GHG emissions. We use a life-cycle-based carbon footprint approach to evaluate the well-to-wheel GHG emissions for the energy chain used by vehicles, by coupling projections from the IAM IMAGE with process-based material and energy inventories from life-cycle assessment (LCA)^92^. The IAM IMAGE is a dynamic integrated assessment framework developed by the Netherlands’ Environmental Assessment Agency to analyze global issues, impacts, and challenges that tend to occur at different geographic scales in different parts of the world^66^. We use the three energy transition scenarios, IMAGE3.2 SSP2-RCP6 (3.5 °C), IMAGE3.2 SSP-RCP26 (2 °C), and IMAGE3.2 SSP2-RCP19 (1.5 °C), to reflect the penetration levels of renewable energy and the extent of negative emission technologies are deployed.

### Vehicle stock and flow modeling

Total vehicle stock in each region is simulated based on population and per-capita vehicle ownership. There are various methods to predict prospective vehicle ownership in a country, such as logistic, Gompertz functions, Logarithmic Logistic, and Cumulative Normal^93^. The Gompertz function is selected as it is more predictive regarding region-specific income levels^94^. This means that per-capita vehicle ownership in a country is driven by economic development. We represent the relationship between vehicle ownership per 1000 capita and gross domestic production (GDP) per capita via a modified Gompertz Model^95^ that considers temporal lags in the adjustment of the vehicle stock responding to income changes in each country. Based on the vehicle stock, dMFA is employed to compute the inflows of new vehicle sales and outflows of retired vehicles. An operable Python-based framework called the Open Dynamic Material Systems Model^96^ was used to perform the dMFA. We select the Weibull distribution to demonstrate the lifetime distributions of all types of vehicle types in our study. A shape parameter and a scale parameter need to be set up to define a Weibull distribution. The shape parameter ranges from 1.89 to 6.32 in past studies^23,24,28,44,50,51,57,97,98^, and we use the median of 4.11 as the shape parameter. A 15-year vehicle lifetime is assumed to estimate the scale parameter^28,41,44,56,99^. We consider four EV market penetration scenarios: S40%, S60%, S80%, and S100%, which represent the exact shares (40–100%) of the overall vehicle market that the EV stock will reach in 2050. The market shares of BEVs, PHEVs, and FCEVs in different regions are assumed based on the IEA^100^. The ICEV market share is assumed based on the BLUE Map scenario from IEA^101^. The share of LDV and HDV was obtained from the ANL^67^ and the study^42^.

### Vehicle categorization

We divide the road fleets based on two categorizations: from the powertrain-wise perspective and from the transportation-mode perspective. The powertrain classification is further divided into EVs and ICEVs. EVs can be normally categorized into four types based on the vehicle hybridization levels^102^: BEVs, PHEVs, HEVs, and FCEVs. BEVs operate solely on the electricity stored in LIBs as the propulsion source, therefore, they usually have the largest LIB pack than other EVs to ensure a long range^102^. PHEVs and HEVs propel through an electric motor as well as an internal combustion engine. PHEVs have a larger LIB pack than HEVs, as PHEVs can be charged by connecting to the local power grid; the LIB packs in HEVs just aim to recover energy from regenerative braking and provide supplemental power to the electric traction motor^102^. Because HEVs are primarily propelled by fossil fuels, we regard HEVs as ICEVs in our simulation. The hydrogen FCEVs can also be seen as EVs because they also propel through electricity and an electric motor^103^. The difference is FCEVs use a built-in electrochemical cell to convert hydrogen into electricity rather than drawing electricity from a battery only. ICEVs include diesel HEVs, gasoline HEVs, diesel ICEVs, gasoline ICEVs, natural gas ICEVs. Transportation sector-wise categorization includes light-duty passenger vehicles (cars), light-duty commercial vehicles (vans and light-duty trucks), heavy-duty commercial vehicles (heavy-duty trucks), and heavy-duty passenger vehicles (buses). Non-road vehicles such as airplanes, rail, ferries, and two/three-wheelers are omitted in this study.

### Critical metal requirements

The material requirements for EV batteries depend on the development of the battery technologies and the required battery capacity per vehicle type. The PGM loading of fuel cells and catalytic converters depends on the power of a vehicle. We only account for the direct material requirement to support the transportation service, and the indirect material use, such as loss in ore extraction and processing, and battery production scraps, are not included. The key assumptions and data sources on vehicle power, battery capacity, battery material composition, and PGM loading are presented in Supplementary Table S1. We do not model the lifetime of EV batteries separately but link the survival of the battery packs to the vehicle lifetime. For light-duty vehicles, it is assumed that the average service life of EV batteries is the same as that of EVs^104^; heavy-duty BEVs, PHEVs, and FCEVs need to replace one battery pack within their lifetime^104^.

### End-of-life battery treatment

After automotive use, there are four main approaches to recovering the retired EV batteries^105^: (1) second use as ESSs; (2) pyrometallurgical processing; (3) hydrometallurgical processing; (4) direct cathode reuse. Retired EV batteries retain a rather high energy storage capacity after their first life in EVs. Therefore, the values of the resources contained in EoL batteries are not entirely exploited if they are sent to a recycling plant. The EoL battery packs can be entirely removed from EVs for second use as ESSs. All EV batteries reach 80% of initial energy storage capacity at the end of their first life^74^. The second use of EV batteries will delay the recirculation of critical metals for 2–20 years^74^. The remaining three approaches can instantly extract target materials from EoL batteries. Pyrometallurgical recycling aims to recycle critical metals by smelting entire batteries, while hydrometallurgical recycling involves acid leaching and subsequent recovery of battery materials. Direct cathode reuse, which involves recovering cathode materials while maintaining their chemical structures, is more ecologically and environmentally viable compared to pyrometallurgy and hydrometallurgy^74^. As catalytic converters are not reused, the contained PGMs are immediately recovered and recycled.

Regarding the definition of recycling, Weil and Ziemann^106^ explained the concepts of collection rate, recycling efficiency, and recycling rate. To simplify LIBs recycling, we assume the collection rate and recycling efficiency are unified as the recycling rate. Besides, we simplify pyrometallurgical, hydrometallurgical, and direct recycling into “recycling”. Therefore, this study considers two EoL treatment methods for EoL LIBs: recycling without second use and recycling after the second use. Under the first recycling scenario, all EoL LIBs and catalytic converters are immediately recycled. The assumption on the recycling rate of each metal is listed in Supplementary Table S1. In the second-use scenario, all the EoL LIBs are second-used in a cascaded way as ESSs, and the catalytic converters are recycled immediately. After the second use, LIBs are recycled. It is assumed that the chemical composition of EoL LIBs remains the same after the second use. The lifetime of the second-used batteries is subject to the Weibull distribution with an average life span of 10 years^74,99^.

### Transportation market

The data on the passenger and freight traffic activities of the regions under study is collected and adapted from IEA, OECD, Eurostat, and NationMaster. The obtained passenger and freight transportation activities are shown in Fig. 1c. The data for the transportation market is derived from two sources—external statistics and the results of the dMFA model. The ITF forecasted the share of public and private passenger transportation till 2050. It is assumed that there is a shift from the future demand toward public means (buses) of road transportation. Thus, the market share of private car transportation activities decrease from 71% in 2010 to 62% in 2050. Regarding freight transportation, the last mile transportation by vans accounts for 28–35%, and long-haul transportation makes up 65–72%. The transportation shares for each vehicle category for public and private passenger transportation, and last-mile and long-haul transportation are assumed based on the annual stock share from the dMFA model. The detailed data sources are shown in Supplementary Table S1.

### Energy system transition

The energy transition scenarios are established by combining Shared Socio-economic Pathway (SSPs) and Representative Concentration Pathway (RCP) scenarios. The SSP-RCP concept is an emerging scenario framework that facilitates the integrated analysis of future climate impacts. The SSP-RCP framework can provide region-specific and long-term energy transition background projections, which can serve as a basis for life-cycle inventory modeling^107^. The SSP scenarios were established based on five alternative socio-economic developmental patterns: sustainable (SSP1), middle-of-the-road (SSP2), regional rivalry (SSP3), inequality (SSP4), and fossil-fueled (SSP5)^108^. We use the “middle-of-the-road” SSP2 pathway that anticipates a moderate population and GDP growth in line with historic development. RCP scenarios are radiative forcing trajectories proposed by IPCC^109^, including a concentration range of 1.9–6.0 W/m^2^. The Netherlands Environmental Assessment Agency developed the IAM framework IMAGE 3.2 to address global environmental, energy, and resource challenges^66^. SSP2-RCP6 (middle-of-the-road development with a radiative forcing of 6.0 W/m^2^ by 2100), SSP2-RCP26, and SSP2-RCP19 scenarios from the IMAGE 3.2 framework are selected to examine three different energy transition pathways to limit global temperature rise by 3.5, 2, and 1.5 °C by the end of this century relative to pre-industrial levels, respectively^66^. The unit fuel emission intensities are modeled by integrating the data from the SSP-RCP framework from the IAM IMAGE 3.2^66^ in the life-cycle inventory database *ecoinvent 3.8*^110^ (cut-off system model). Compared to the 3.5 °C scenario, the 2 °C scenario has a higher level of decarbonized transportation energy systems and applies CCS technologies^111^. The 1.5 °C scenario has even deeper decarbonized energy systems and CCS deployment. Moreover, in the 2 °C and 1.5 °C scenarios, higher shares of bioethanol and biodiesel are blended into gasoline and diesel.

### Carbon footprint assessment

We conduct a life-cycle-based carbon footprint analysis to compute the GHG emissions caused by the supply of energy to the passenger and freight transportation sectors. To that effect, regional and temporal developments regarding alternative fuels, including electricity, hydrogen, and biofuels, in the 16 regions of the IAM scenario are integrated into the life-cycle inventory database *ecoinvent 3.8*^110^ using the open-source Python tool *premise* v1.2.6^75^. Compared with the classical carbon footprint analysis that only accounts for carbon emission and sequestration flows, a life-cycle-based carbon footprint compiles energy and material inventory data throughout the investigated system boundaries^92^. Hence, the life-cycle GHG emissions from fuels supplied to operate vehicles are calculated for each region and time step under every climate scenario. The carbon footprint of fuels for road transportation comprises the embodied GHG emissions from fuel production and operational emissions, specifically including direct and indirect emissions from every relevant step along the well-to-wheel supply chain of fuels. For conventional fuels, it ranges from refining crude oil to distributing the refined fuel product at the fueling station. For electricity and electricity-based fuels (i.e., hydrogen), it includes the extraction of primary energy as well as its conversion, distribution, and transmission at low voltage to the consumer (i.e., the BEV owner). Also, a total 1.5% mass loss of hydrogen is considered to reflect leakages along the different components of the hydrogen supply chain^112^ (i.e., venting of electrolyzers, compression, storage, and evaporation). In relation to the combustion of fuel during vehicle operations, three GHGs are considered, namely CO_2_, N_2_O, and CO. Emissions of CO_2_ are based on the stoichiometric reaction of the fuel oxidization, while emission factors for CO and N_2_O for the different vehicles and fuels are provided by European Environment Agency’s air pollutant emission inventory guidebook 2019^113^. The emissions of other pollutants from the vehicle exhaust (e.g., NOx, NH_3_, SOx, PMs, etc.) are excluded because their contribution to climate change is negligible or inexistent^113^. Moreover, the emissions from battery and vehicle manufacture, road and vehicle maintenance, and EoL battery and vehicle treatment are also excluded. Life-cycle GHG emissions from producing biofuels are considered (i.e., farming, bioethanol conversion, and distribution), but the CO_2_ emissions stemming from its combustion match the uptake of CO_2_ by growing bioenergy feedstock and is therefore considered as net zero^114^. The environmental indicator, Global Warming Potential (GWP), expressed in kg CO_2_-eq from IPCC GWP 100a^115^, is used to calculate the warming caused by the release of GHG integrated over 100 years. There is an exception for hydrogen, which GWP 100a impact from leakages is calculated using the central estimate provided by Warwick et al. (i.e., 11 kg CO_2_-eq/kg hydrogen)^116^, as hydrogen is an indirect GHG and, as such, is not listed in the current IPCC GWP 100a impact method^115^. Finally, the analysis is performed by the advanced LCA framework Brightway2^117^ to assess the GHG emissions per unit of fuel, including electricity, gasoline, bioethanol, natural gas, diesel, biodiesel, and hydrogen.

## Supplementary information

Supplementary Information

## Source data

Source Data

## Data Availability

The data that support the findings of this study are available upon request. Source data are provided with this paper.
