# Addendum: Trade-off between critical metal requirement and transportation decarbonization in automotive electrification

**DOI:** 10.1038/s41467-025-57270-2

**Published:** 2025-03-13

**Authors:** Chunbo Zhang, Xiang Zhao, Romain Sacchi, Fengqi You

**Affiliations:** 1 Robert Frederick Smith School of Chemical and Biomolecular Engineering, Cornell University, Ithaca, New York 14853 USA; 2 Systems Engineering, Cornell University, Ithaca, New York 14853 USA; 3 Technology Assessment Group, Laboratory for Energy Systems Analysis, Paul Scherrer Institute, Villigen, Switzerland; 4 Cornell Atkinson Center for Sustainability, Cornell University, Ithaca, New York 14853 USA

**Keywords:** Energy infrastructure, Climate sciences, Energy and society

Addendum to: *Nature Communications* 10.1038/s41467-023-37373-4, published online 11 April 2023

The original version of this article contained conceptual ambiguousness in the “Requirement of critical metals” of the Results section. Clarifications that need to be made are presented as follows.

The conceptual ambiguousness of the share of gross reserves being assumed to be available for batteries and automotive catalysts as opposed to their use in other applications needs clarification. The data for gross reserves of each critical metal in 2020 was collected from the USGS^1^ (ref. 73 in the main text): 21 Tg for lithium, 94 Tg for nickel, 7 Tg for cobalt, 1300 Tg for manganese, and 69 Gg for platinum group metals (PMGs). It could be misleading as we did not clarify in the main text the assumptions on the share of gross reserves being available for battery production. These assumptions are based on two steps. First, we estimated what share of the annual mining of each critical metals went into battery production in 2020, based on our own material demand estimate and annual mining data from USGS. We estimated that in 2020, 74% of annual lithium mining^2^, 11% of nickel mining^3^, 40% of cobalt mining^4^, and 15% of manganese mining^5^ were used in the battery industry, and 39% of platinum group metal mining were used in automotive catalysts^6^. In the second step, we assumed that the demand in these metals for all other applications will increase as much as the demand for batteries increases. For that reason, we assumed that only 74% of the lithium reserves, 11% of nickel reserves, 40% of cobalt reserves, and 15% of manganese reserves could be available for batteries. Details of the demand-mining comparison for the shares can be found in Fig. S23 of the Supporting Information. We identified that lithium, nickel, and cobalt are more likely to suffer supply risks than manganese and PGMs, based on each metal’s cumulative demands and the share of its reserves assumed to be available for batteries. Although the use-end share of lithium and cobalt for clean energy technologies may rise up to around 90% and 70% in 2040^7^, the cumulative demands of lithium and cobalt under the S80% (22 Tg and 9 Tg) and S100% (27 Tg and 12 Tg) penetration rates exceed their total reserves (21 Tg and 7 Tg) in 2020. Nickel has greater amount of reserves (94 Tg) compared to lithium and cobalt in 2020. However, about 72% of the nickel currently produced is used for steel production, while only 11% is used for batteries^3^. Steel is primary used for six sectors—buildings, infrastructure, vehicles, mechanical and electrical equipment, consumer goods, and pre-consumer scrap^8^. In-use steel stocks in the three sectors of construction, infrastructure and vehicles are estimated to increase from 14 Pg in 2010 to 55 Pg in 2050^9^, with the cumulative demand around 68 Pg. Nickel content ranges from approximately 0.1% in steel, calculated based on the crude steel production in 2020 (1.86 Pg)^10^ and the nickel produced for steel (1.85 Tg)^1,3^ in 2020. This results in a cumulative nickel demand for steel production of at least 68 Tg in 2010–2050, and even more if demand for electromechanical equipment, consumer goods and pre-consumer scrap is taken into account. Therefore, the tremendous demand of nickel for steel production may pose a risk to the supply of nickel demand for batteries. For lithium and cobalt, such competing demand from other applications is currently not foreseeable. However, the assumption that the demand in these critical metals increases in all other applications as much as the demand for batteries increases may thus underestimate the amount of lithium, cobalt, and nickel being available for battery production. Also, with continuous exploration of mineral deposits and new mining technologies the gross reserves of critical metals tend to increase.

Additionally, in the version of the article initially published, in the third paragraph of the “Requirement of critical metals” in the Results section, the text “…the cumulative demand for manganese in 2050 (3–9 Tg)” should have read “…the cumulative demand for manganese in 2050 (3–7 Tg)”. The text “Global proven lithium reserves for EV batteries in 2020 are around 16 Tg, 44% and 22% of which are in Chile and Australia, respectively. The cumulative demand for lithium ranges from 12–26 Tg, determined by the level of EV penetration. Therefore, lithium supplies fall short under a high EV penetration rate (60–100%). Nickel and cobalt suffer from their supply risks as their reserves in 2020 (7 Tg for nickel and 3 Tg for cobalt^73^) fail to meet the cumulative demand by 2050 of 28–63 Tg and 5–11 Tg, respectively^73^” has been corrected to read “Global proven lithium reserves in 2020 are around 21 Tg, 44% and 22% of which are in Chile and Australia, respectively. It is assumed that 74% of these, or 16 Tg, are available for batteries. The cumulative demand for lithium ranges from 11–27 Tg, determined by the level of EV penetration. Therefore, lithium supplies may only fall short under the high EV penetration rates (80–100%), depending on what share of lithium reserves are assumed to be available for batteries. However, with more and more lithium deposits being discovered, and explorations and new mining technologies turning resources into reserves, available lithium reserves are expected to increase and close this gap. Based on the share of reserves in 2020 being available for battery production (10 Tg for nickel and 3 Tg for cobalt^73^), nickel and cobalt may fail to meet the cumulative demand by 2050 of 27–63 Tg and 5–12 Tg, respectively^73^. If a larger share or even all the 2020 reserves of 94 Tg for nickel and 7 Tg for cobalt would be available for battery production, there would be no supply risk for nickel. For cobalt, this gap could be closed by a stronger shift to low-cobalt or no cobalt containing battery chemistries.” Finally, two sentences below (“The three main nickel reserves...”), “Argentina (20 Tg)” has been corrected to “Australia (20 Tg)”. These corrections have been made to the HTML and PDF versions of the article.

We would like to apologize for any inconvenience and state that the extended discussion in this Addendum does not affect the scientific conclusions of the manuscript.

## Supplementary information

Updated Supplementary Information
