# Supplementary material for: Trade-off between critical metal requirement and transportation decarbonization in automotive electrification
Source: Nat Commun. 2023 Apr 11;14:1616. doi: 10.1038/s41467-023-37373-4 (PMC10090058; doi:10.1038/s41467-023-37373-4)
Supplement: Supplementary file 1 — Supplementary Information [file 41467_2023_37373_MOESM1_ESM.pdf]

SUPPORTING INFORMATION

for

Trade-off between critical metal requirement and transportation decarbonization in automotive electrification

Chunbo Zhang[1], Xiang Zhao[2], Romain Sacchi[3], Fengqi You[1,2*]

1. Robert Frederick Smith School of Chemical and Biomolecular Engineering, Cornell University, Ithaca, New York 14853, USA

2. Systems Engineering, Cornell University, Ithaca, New York 14853, USA

3. Technology Assessment Group, Laboratory for Energy Systems Analysis, Paul Scherrer Institut, Villigen, Switzerland

[*] Corresponding author. Phone: (607) 255-1162; Fax: (607) 255-9166; Email: fengqi.you@cornell.edu

Abbreviation list

Abbreviation	Full term
BEV	Battery electric vehicle
CCS	Carbon capture and storage
dMFA	Dynamic material flow analysis
ESS	Energy storage system
EV	Electric vehicle
EoL	End-of-life
FCEV	Fuel cell electric vehicle
GHG	Greenhouse gas
HDCV	Heavy-duty commercial vehicle
HDPV	Heavy-duty passenger vehicle
HEV	Hybrid electric vehicle
IAM	Integrated assessment model
ICEV	Internal combustion engine vehicle
IEA	International Energy Agency
LDCV	Light-duty commercial vehicle
LDPV	Light-duty passenger vehicle
LFP	Lithium iron phosphate battery
LIB	Lithium-ion battery
LCA	Life cycle assessment
Li-air	Lithium-air battery
Li-S	Lithium-sulfur battery
NCA	Lithium nickel cobalt aluminum oxide battery
NMC	Lithium nickel manganese cobalt oxide battery
PHEV	Plug-in hybrid electric vehicle
RCP	Representative Concentration Pathways
SSP	Shared Socioeconomic Pathways

1. Model framework and key parameters

The overall model framework of this study is shown in Figure S1. This model consists of a (i) dynamic material flow-stock analysis (dMFA) module that aims to simulate the stock and flow of vehicles and associated material requirements and (ii) a carbon footprint module is used to assess the fuel-induced road transportation greenhouse gas (GHG) emissions.

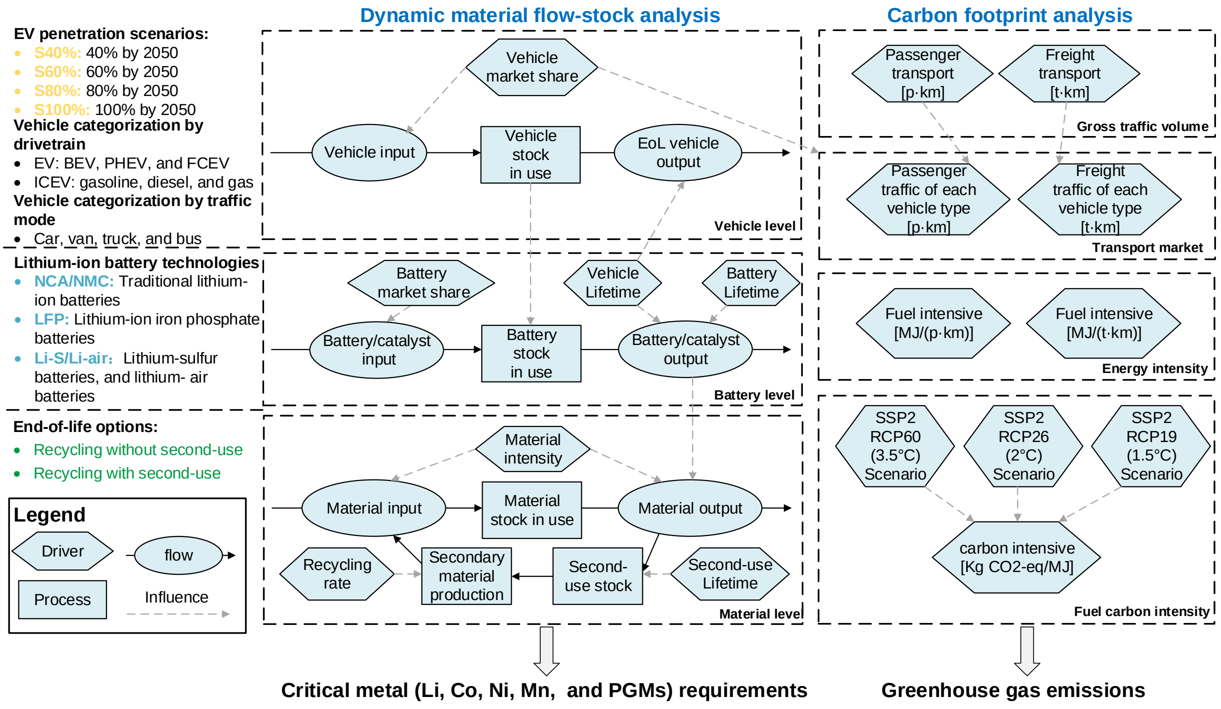

Figure S1. Schematic representation of the methodological route of this study. Note: hexagons indicate drivers and determinants, rectangles represent processes, ovals with solid lines denote flows, and dashed lines with arrows denote influences between two variables. ICEV: internal combustion engine vehicle, EV: electric vehicle, NMC: lithium nickel manganese cobalt oxide battery, NCA: lithium nickel cobalt aluminum oxide battery, LFP: lithium iron phosphate battery, Li-S: lithium-sulfur battery, Li-air: lithium-air battery, RCP: Representative Concentration Pathways, SSP: Shared Socioeconomic Pathways

Key parameters in this study include: (1) geographical scope and territorial division, (2) population, (3) gross domestic product (GDP), (4) vehicle ownership, (5) vehicle category; (6) electric vehicle (EV) and internal combustion engine vehicle (ICEV) market, (7) Battery and catalytic converter material intensity, (8) EV battery market, (9) EV lithium-ion battery (LIB) capacity, (10) Engine power, (11) vehicle and battery lifetime, (12) freight and passenger traffic volume, (13) transportation market, (14) energy transition and fuel emission, (15) material recycling. Key parameters and their assumptions are listed in Table S1.

Table S1 Key parameters and assumptions of this model

Key parameters	Descriptions/Assumptions
Geographical scope and territorial division	This study considers 48 countries that are further divided into 16 regions based on the framework of integrated assessment model IMAGE framework[1]: (1) Oceania (OCE): Australia, New Zealand; (2) Western Europe (WEU): Austria, Belgium, Denmark, Finland, France, Germany, Greece, Iceland, Ireland, Italy, Luxembourg, Liechtenstein, Malta, Netherlands, Norway, Portugal, Spain, Sweden, Switzerland, and the United Kingdom; (3) Central Europe (CEU): Bulgaria, Croatia, Cyprus, Czech, Estonia, Hungary, Latvia, Lithuania, Poland, Romania, Slovakia, and Slovenia; (4) Southeast Asia (SEAS): Malaysia, and Thailand; (5) South Africa (SAF): South Africa; (6) Rest of south America (RSAM): Chile; (7) Turkey (TUK); (8) Mexico (MEX); (9) India (INDIA); (10) Japan (JAP); (11) China (CHN); (12)Canada (CAN); (13)South Korea (KOR); (14) Indonesia region (INDO): Indonesia; (15) the United States (USA); and (16) Brazil (BRA).<br>Those 16 worldwide regions are further aggregated into five major economic entities: (1) China, (2) the United States, (3) Europe (Western Europe + Central Europe), (4) India, and (5) the rest.
Population	The population from 2010 to 2050 is from the World Bank[2].
Gross domestic product	The GDP of each country is obtained from the OECD[3] in Million USD at constant prices and Purchasing Power Parities (PPPs) of 2015, which is converted into 1995 level by the Purchasing power parity conversion factors from OECD[4].
Vehicle ownership	Vehicle ownership per 1,000 capita is assumed to grow based on regional historical vehicle ownership levels, population, and GDP. A modified Gompertz model was used to simulate how GDP and population drive the vehicle ownership level[5].
Vehicle category	Powertrain-based categorization: (i) Electric vehicle (EVs): battery electric vehicles (BEVs), plug-in electric vehicles (PHEVs), and fuel cell electric vehicles (FCEVs); (ii) internal combustion engine vehicles (ICEVs): gasoline-based ICEVs, diesel-based ICEVs, gasoline-based hybrid electric vehicles (HEVs), diesel-based HEVs, natural gas-based ICEVs.<br>Transport mode-based categorization: (i) light-duty passenger vehicles (LDPVs): cars; (ii) light-duty commercial vehicles (LDCVs): vans and light-duty trucks; (iii) heavy-duty passenger vehicles (HDPV): bus; (iv) heavy-duty commercial vehicles (HDCVs): heavy-duty trucks.
Vehicle market	The EV market share is assumed to increase based on the prediction of four World Energy Outlook-2021 (WEO-2021) scenarios from the International Energy Agency (IEA)[6–8], which considers regional differences in carbon mitigation ambitions and existing EV policies. Based on the four WEO-2021 scenarios (Stated Policies Scenario, Announced Pledges Scenario, Sustainable Development Scenario, Net Zero Emissions by 2050 Scenario) we created four new EV penetration scenarios, namely S40%, S60%, S80%, and S100%, which

	aims to realize the EV market stock of 40%–100% in 2050. The ICEV market share is assumed based on the BLUE Map scenario from IEA[9]. The share of LDV and HDV was obtained from the ANL[10] and the study[11].
Material intensity	The material compositions of EV Li-ion batteries are calculated based on the BatPaC model version 3.1[12] and the past study[13]. The data on PGM loading of vehicle catalysts is derived from the study[11].
Battery market	EV battery technologies of lithium nickel cobalt aluminum oxide (NCA) batteries, lithium nickel manganese cobalt oxide (NMC) batteries (NCA, NMC111, NMC523, NMC622, NMC811, and NMC955), lithium-sulfur (Li-S) batteries, and lithium-air batteries (Li-air) are considered. The market share of those battery technologies are calculated based on the study[13].
Battery capacity	The LIB battery capacity of BEVs and PHEVs is modified based on the study[13,14]. The battery capacity of light-duty FCEVs is not considered[15]. The battery capacity of heavy-duty is assumed based on the GREET model[16] and the study[17].
Engine power	The engine power (kW) of ICEVs and FCEVs is assumed based on the study[11].
Vehicle and battery lifetime	The average lifetime of a vehicle is assumed to be 15 years[13,18,19]. The average lifetime Li-ion batteries for light-duty EVs is assumed to be the same as the EVs, 15 years, considering battery lifetime extension in the furture[13]. For heavy-duty BEVs, PHEVs, and FCEVs, lower battery lifetime leads to battery replacement[16]. The lifetime of use EoL battery as an energy storage system is assumed to be 10 years[20,21]. Weibull distribution is used to simulate the survival distribution of vehicles.
Transportation activities	The data on the freight (t·km) and passenger (p·km) transportation are collected from multiple sources. The historical freight transportation data is from the ITF[22], Eurostat[23], and NationMaster[24]. The historic passenger transportation data is from the ITF[22], Eurostat[25], and NationMaster[26]. The forecasted freight transportation data was collected from IEA[27]. The data on forecasted passenger transportation activities was estimated based on population growth.
Transportation market	The transportation market is assumed based on the vehicle stock from the dMFA model and the ITF[28]. ITF forecasted the share of passenger public (buses) and private (cars) transportation till 2050[28]. The last mile transportation by vans would account for 28[29]–35%[30]. Therefore, the share of long-haul transportation can be calculated, which is 65%–72%.
Energy transition and fuel emission	The energy transition is simulated by referring to the SSP2 scenario in the IAM IMAGE 3.2[1]. Three pathways under the SSP2 scenario, namely SSP2-RCP60, SSP2-RCP26, and SSP2-RCP19, are selected to model the energy transition. Fuel emissions in each scenario were modeled based on the prospective life cycle assessment modeling platform Brightway2[31]. The Ecoinvent 3.8 (cut-off) database, IMAGE 3.2 database[1], and IMAGE-based inventory database PREMISE 1.2.6[32] are used.
Material recycling	The recycling rates for lithium, nickel, cobalt, and manganese are assumed based on the World Bank[33]. It is assumed that the recycling rate of lithium, nickel, cobalt, and manganese will reach 80% in 2030[34]. The recycling rate for PGMs is

	assumed based on the study[11]. Each region is assumed to have the same recycling rate in a year.

2. Vehicle stock and material requirement modeling

Vehicle ownership

We represent the relationship between vehicle ownership per 1,000 capita and gross domestic production (GDP) per capita via a modified Gompertz Model[5] that considers temporal lags in the adjustment of the vehicle stock responding to income changes, as shown in Eq. (1).

$$O_{n,y} = \gamma_{max}(\theta_R R_{n,y} + \theta_F F_{n,y})\, EXP\left(\alpha EXP(\beta_n GDP_{n,y})\right) + (1 - \theta_R R_{n,y} - \theta_F F_{n,y}) O_{n,y-1} \quad (1)$$

Where:

$O_{n,y}$ denotes vehicle ownership per 1,000 capita
γ_{max} denotes vehicle saturation level
$GDP_{n,y}$ denotes gross domestic product per capita (PPP constant 1995 international $)
θ_R denotes rising income adjustment coefficient ($0 < \theta_R < 1$)
θ_F denotes falling income adjustment coefficient ($0 < \theta_F < 1$)
$R_{n,y}$ denotes a dummy variable ($R_{n,y}=1$ *if* $GDP_{n,y} - GDP_{n,y-1} > 0$ *and* $= 0$ *otherwise*)
$F_{n,y}$ denotes a dummy variable ($F_{n,y}=1$ *if* $GDP_{n,y} - GDP_{n,y-1} < 0$ *and* $= 0$ *otherwise*)
α denotes coefficient ($\alpha<0$)
β_n denotes coefficient ($\beta_n<0$)
n denotes nation
y denotes year

The vehicle ownership per 1,000 capita based on Eq.(1) is shown in Figure S2. The historic data from 1990-2019 is from the International Historical Statistics[35] and NationMaster[36]. The data from 2020 to 2050 was estimated by the modified Gompertz Model.

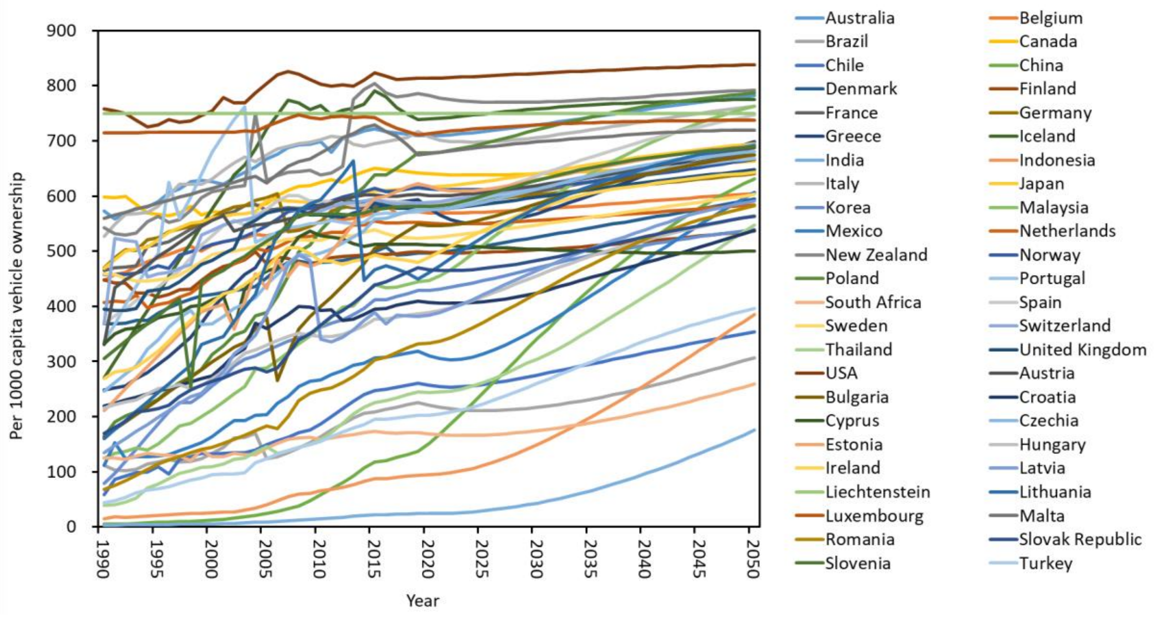

Figure S2. Per 1000 capita vehicle ownership from 1990 to 2050 based on the modified Gompertz Model

Dynamic stock and flow model

The computation of the vehicle stock and flow was based on an operable Python-based framework called the Open Dynamic Material Systems Model developed by Pauliuk and Heeren (2020). We use a stock-driven MFA model to simulate the inflow and outflow. The annual vehicle sales/inflow can be calculated by using Eq. (2). The annual end-of-life (EoL) vehicle outflow is modeled through Eq. (3). We select Weibull distribution to demonstrate the lifetime distributions of vehicles. The Weibull random variables t and t' are characterized by the shape parameter k and a scale parameter λ, as shown in Eq. (4).

$$V_{in}(t) = S(t) - S(t-1) + V_{out}(t), \tag{2}$$

$$V_{out}(t) = \int_{t_0}^{t} V_{in}(t')L_v(t,t')\, dt', \tag{3}$$

$$L(t,t') = \begin{cases} k\lambda^{-k}(t-t')^{k-1}e^{-\frac{(t-t')^k}{\lambda^k}}, & t' < t \\ 0, & t' \geq t \end{cases}, \tag{4}$$

where:

$V_{in}(t)$ denotes the number of vehicle sales in year t (2010, 2050);

$V_{out}(t)$ denotes the amount of EoL vehicles in year t (2010, 2050);

$S(t)$ denotes the vehicle stock in year t (2010, 2050);

$S(t_0)$ denotes the vehicle stock in the initial year 2010;

$L_v(t, t')$ denotes a probability distribution function that presents the probability that a vehicle manufactured in year $t' < t$ will be demolished in year t. k is the shape parameter and λ is the scale parameter λ.

Recycling rate

The recycling rate of each critical metal in this study is shown in Figure S3. Based on the recycling potential of lithium, nickel, cobalt, and manganese[34], we assumed their recycling rates will stabilize at 80% in 2030. The historic recycling rate of lithium, nickel, cobalt, and manganese was modified based on the World Bank's report[33]. The recycling rate of PGMs was modified based on the past study[11].

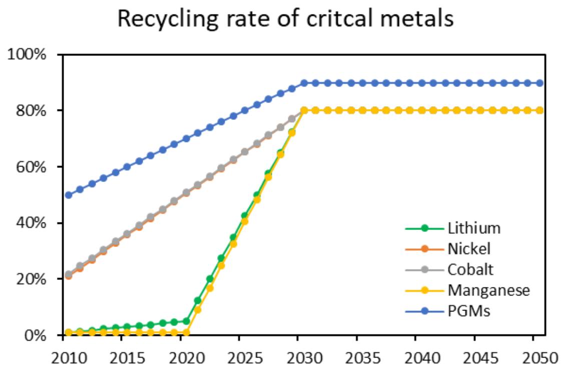

Figure S3. Recycling rate of each critical metal. PGM: platinum group metal

3. Road transportation emission modeling

The road transportation emissions were estimated based on the transportation emission framework of the IPCC[38]. The total transportation emissions can be calculated through four decomposition factors: (i) system-infrastructure modal choice, (ii) fuel carbon intensity, (iii) energy intensity, and (iv) activity. We modified IPCC's transportation emission model and the GHG emissions of road transportation in a region in year y can be calculated via Eq. (5).

$$E_{n,y} = T_{n,y}^{P} \sum_{v} M_{n,y,v}^{P} \left(\sum_{f} F_{n,y,v,f}^{P} C_{n,y,f}^{P} \right) + T_{n,y}^{F} \sum_{v} M_{n,y,v}^{F} \left(\sum_{f} F_{n,y,v,f}^{F} C_{n,y,f}^{F} \right) \quad (5)$$

Where:
E denotes road transportation greenhouse gas emission
T denotes traffic volume
M denotes transportation market share
F denotes fuel intensity
C denotes fuel carbon intensity
P denotes passenger transportation
F denotes freight transportation
n denotes nation
y denotes year
v denotes vehicle category
f denotes fuel category

4. Critical metal requirement modeling

The critical metal requirement is estimated based on four decomposition factors: (i) annual sale of vehicle ($V_{in|n,y,v,f}$), (ii) battery capacity/fuel cell power ($B_{n,y,v,f}$), and (iii) metal loading intensity ($I_{n,y,v,f,m}$). The critical metal requirement of road transportation in a region in year t can be calculated via Eq. (6).

$$Q_{n,y,m} = \sum_{v} V_{in|n,y,v,f} B_{n,y,v,f} I_{n,y,v,f,m} \tag{6}$$

Where:
Q denotes critical metal requirement
V_{in} denotes the amount of vehicle sales
B denotes battery capacity or fuel cell power
I denotes metal loading intensity
n denotes nation
y denotes year
v denotes vehicle category
f denotes fuel category
m denotes the type of required metal

5. EV stock projection

The forecasted EV stock is shown in Figure S4. Transport mode-specific EV stock of the S40%–S100% scenarios are shown in Figure S5–Figure S8. Hybrid electric vehicles (HEVs) are categorized as internal combustion engine vehicles (ICEVs) since the capacity of LIBs in HEVs is negligible (approximately 2 kWh).

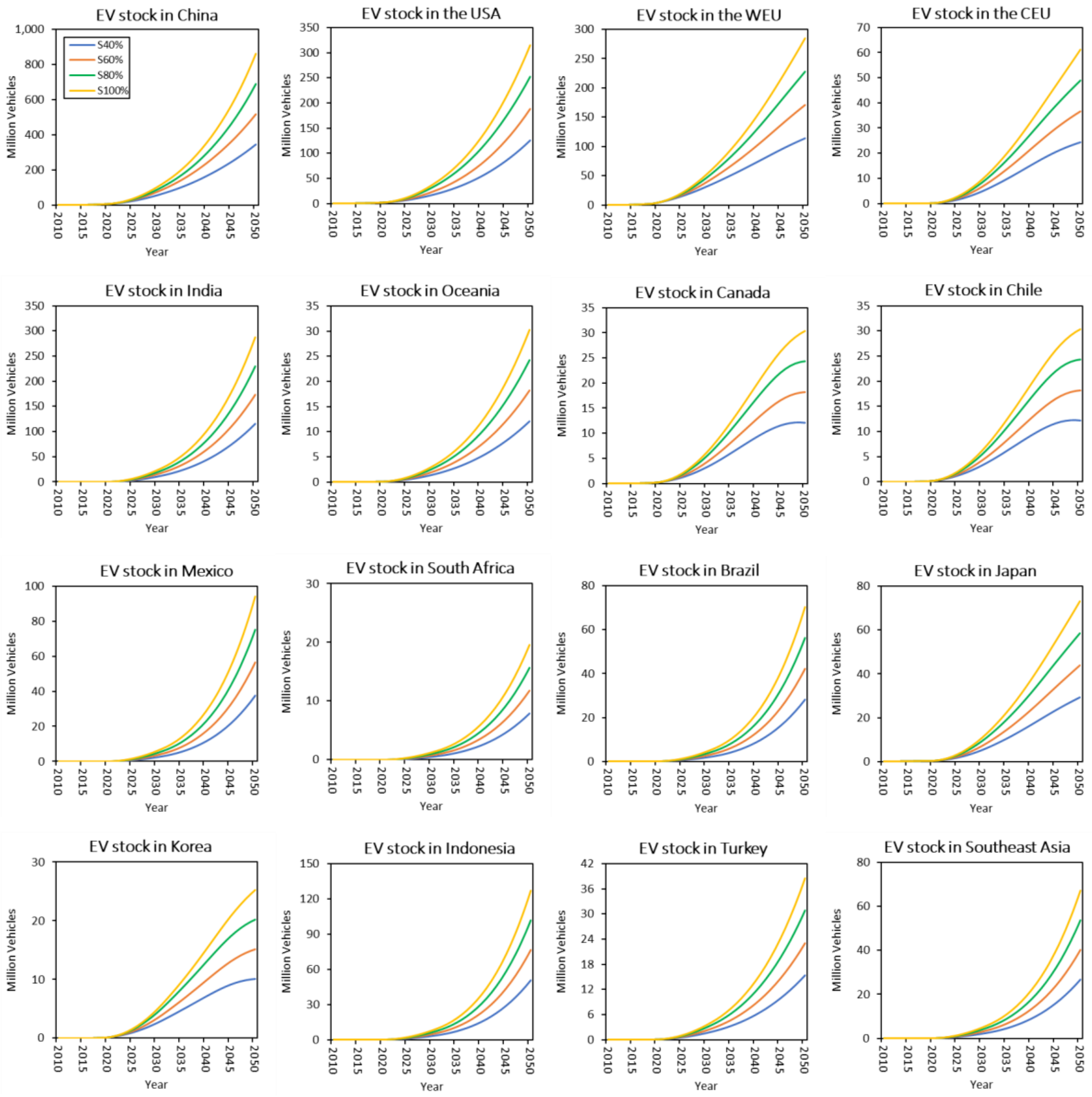

Figure S4. Stocks of electric vehicles (EVs) in the target 16 regions under four EV penetration scenarios. Note: EVs include battery electric vehicles (BEVs), plug-in electric vehicles (PHEVs), and fuel cell electric vehicles (FCEVs). WEU: Western Europe, CEU: Central Europe.

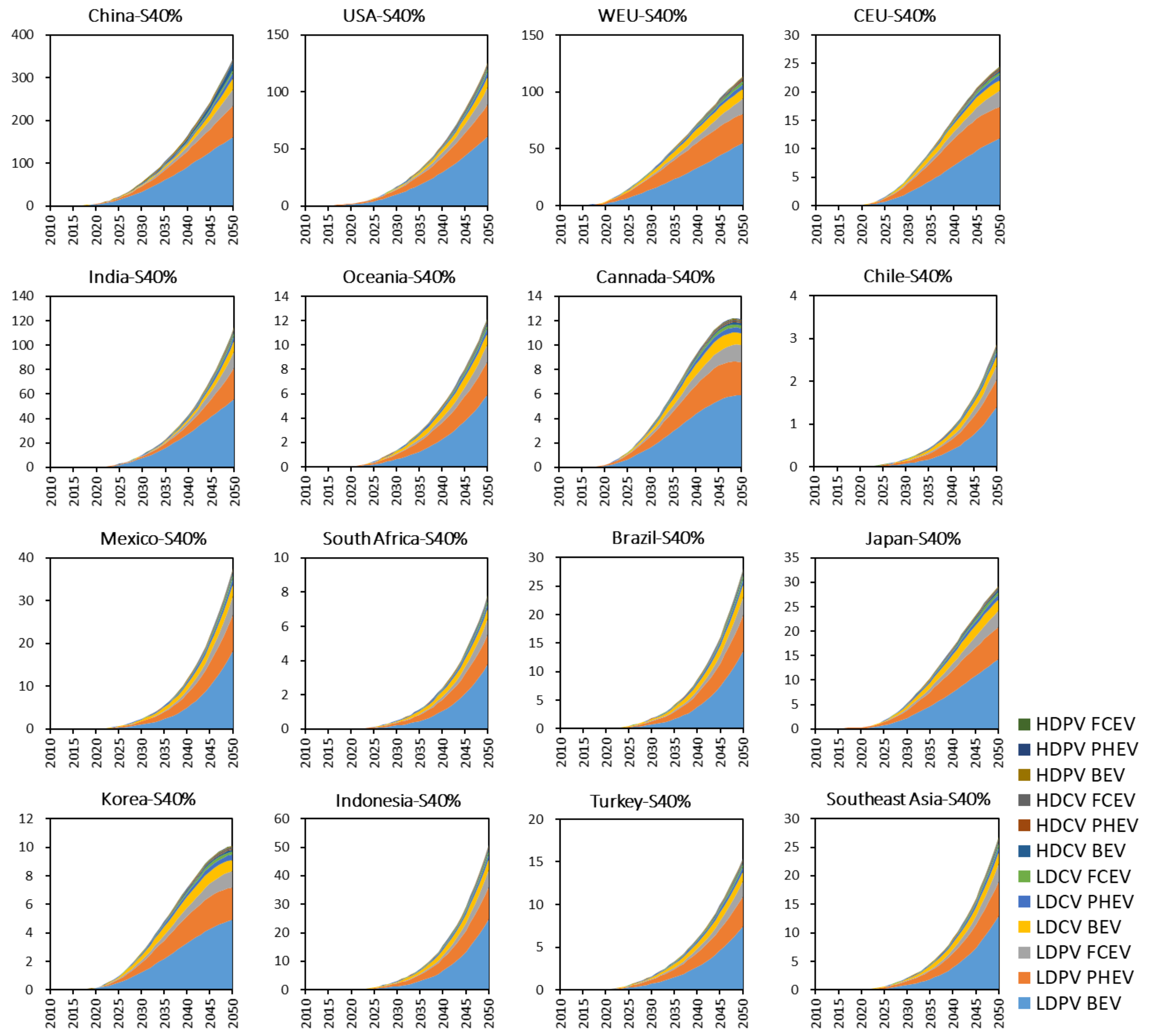

Figure S5. Transport mode-specific stocks (Unit: million vehicles) of electric vehicles (EVs) in the target 16 regions under the S40% scenarios. Note: EVs include battery electric vehicles (BEVs), plug-in hybrid electric vehicles (PHEVs), and fuel cell electric vehicles (FCEVs). LDPV: light-duty passenger vehicle, HDPV: heavy-duty passenger vehicle, LDCV: light-duty commercial vehicle, HDCV: heavy-duty commercial vehicle.

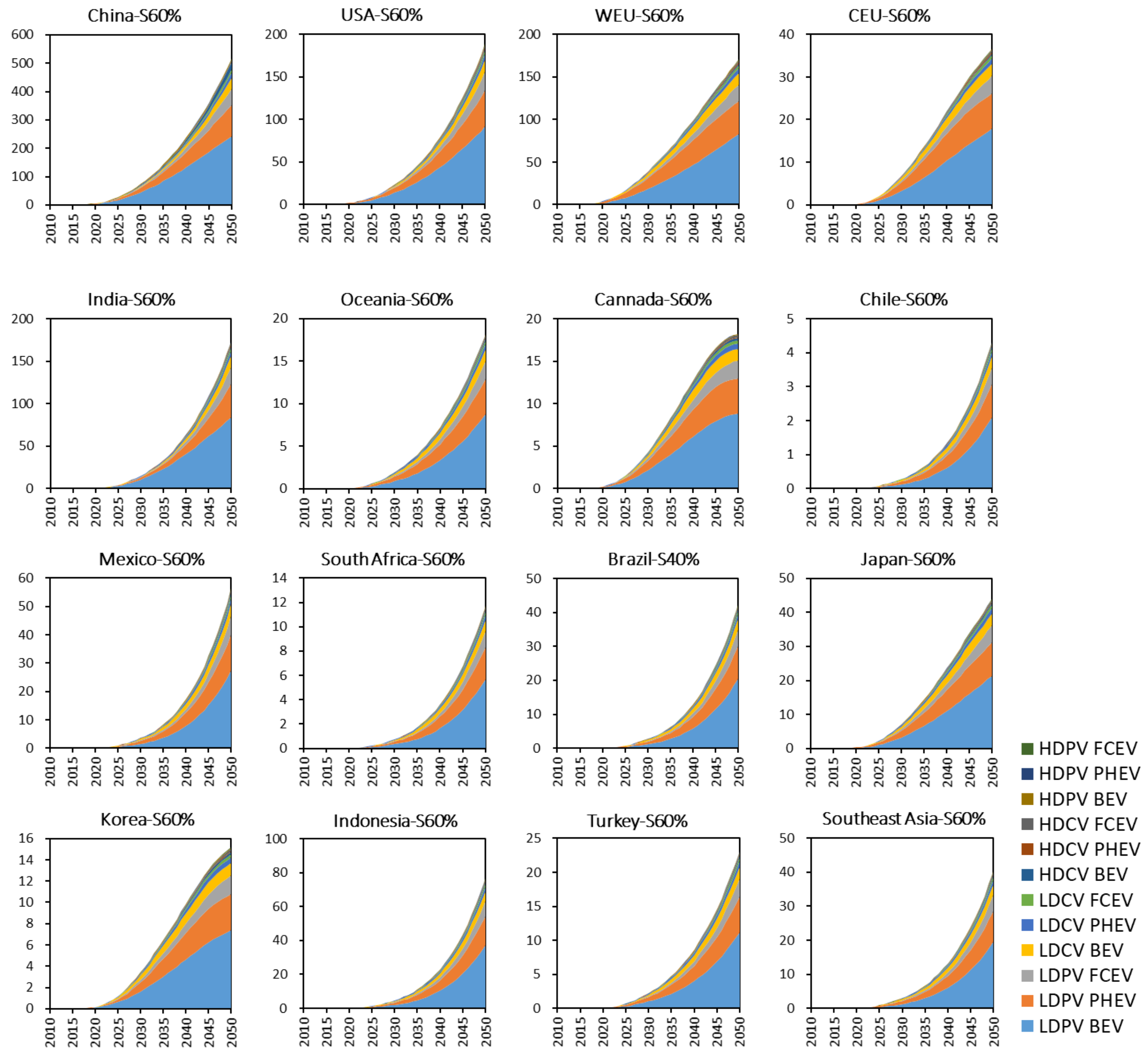

Figure S6. Transport mode-specific stocks (Unit: million vehicles) of electric vehicles (EVs) in the target 16 regions under the S60% scenarios. Note: EVs include battery electric vehicles (BEVs), plug-in hybrid electric vehicles (PHEVs), and fuel cell electric vehicles (FCEVs). LDPV: light-duty passenger vehicle, HDPV: heavy-duty passenger vehicle, LDCV: light-duty commercial vehicle, HDCV: heavy-duty commercial vehicle.

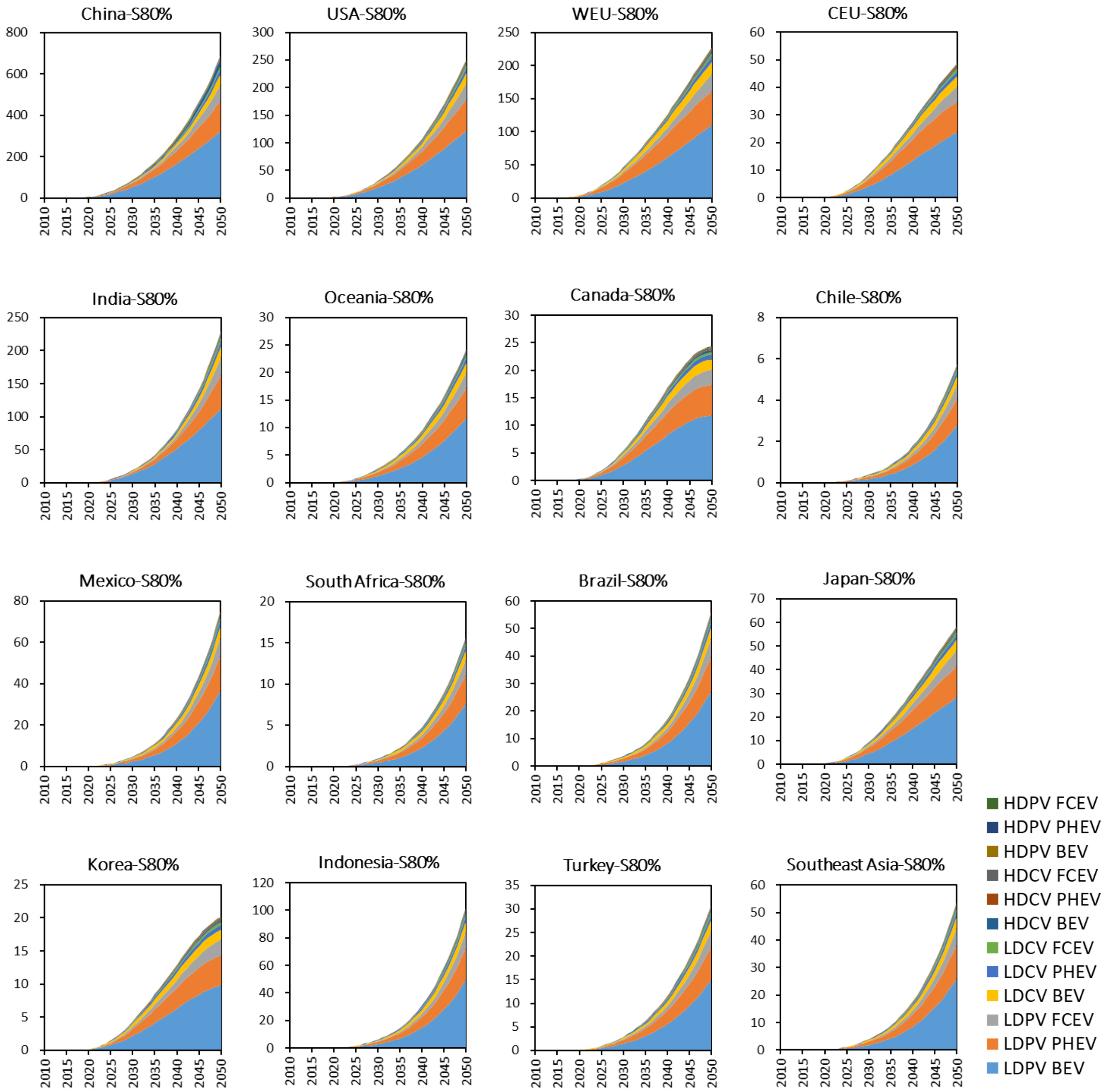

Figure S7. Transport mode-specific stocks (Unit: million vehicles) of electric vehicles (EVs) in the target 16 regions under the S80% scenarios. Note: EVs include battery electric vehicles (BEVs), plug-in hybrid electric vehicles (PHEVs), and fuel cell electric vehicles (FCEVs). LDPV: light-duty passenger vehicle, HDPV: heavy-duty passenger vehicle, LDCV: light-duty commercial vehicle, HDCV: heavy-duty commercial vehicle.

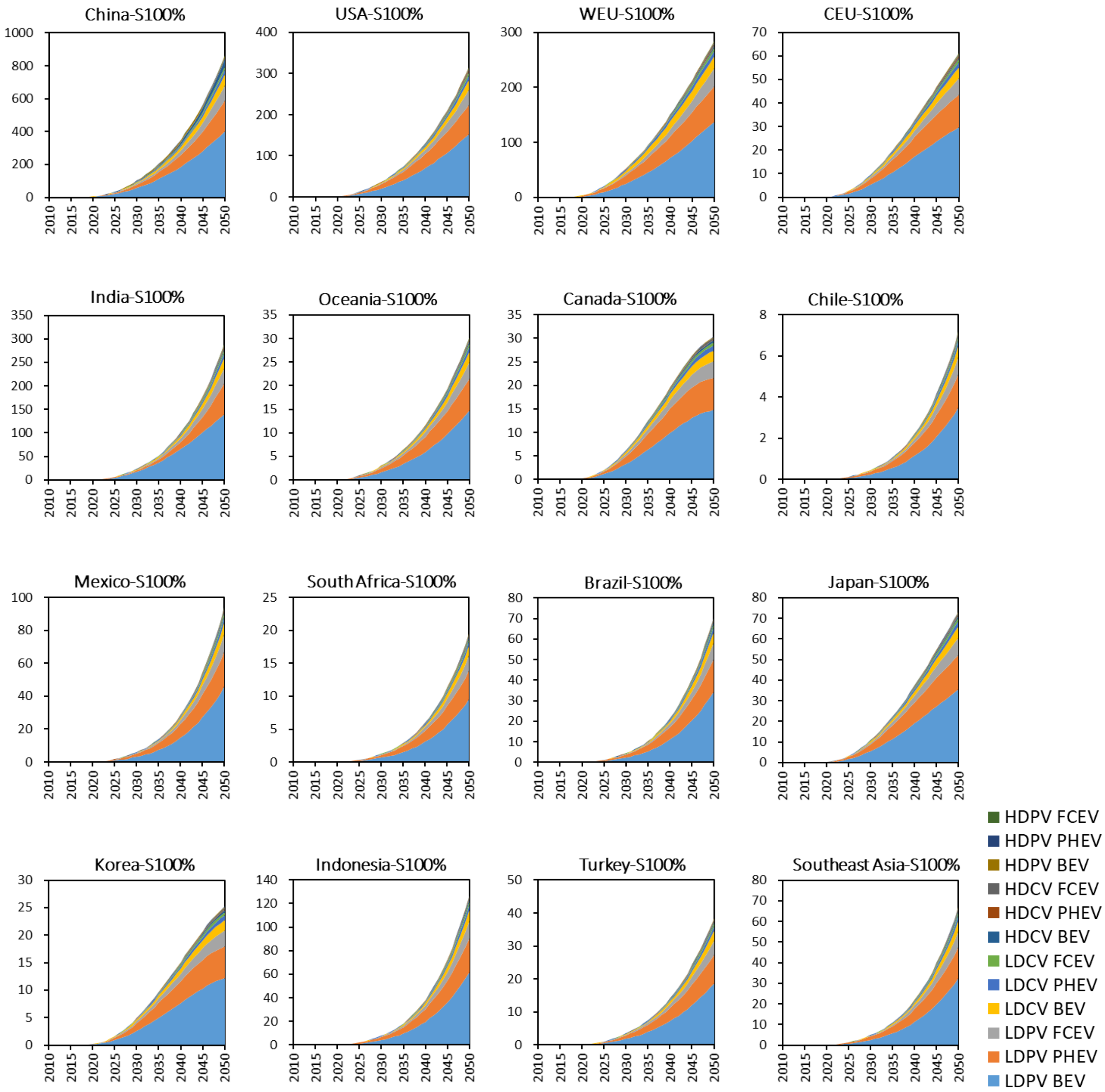

Figure S8. Transport mode-specific stocks (Unit: million vehicles) of electric vehicles (EVs) in the target 16 regions under the S100% scenarios. Note: EVs include battery electric vehicles (BEVs), plug-in hybrid electric vehicles (PHEVs), and fuel cell electric vehicles (FCEVs). LDPV: light-duty passenger vehicle, HDPV: heavy-duty passenger vehicle, LDCV: light-duty commercial vehicle, HDCV: heavy-duty commercial vehicle.

6. ICEV stock projection

The forecasted ICEV stock is shown in Figure S9. Transport mode-specific ICEV stock of the S40%–S100% scenarios are shown in Figure S10–Figure S13.

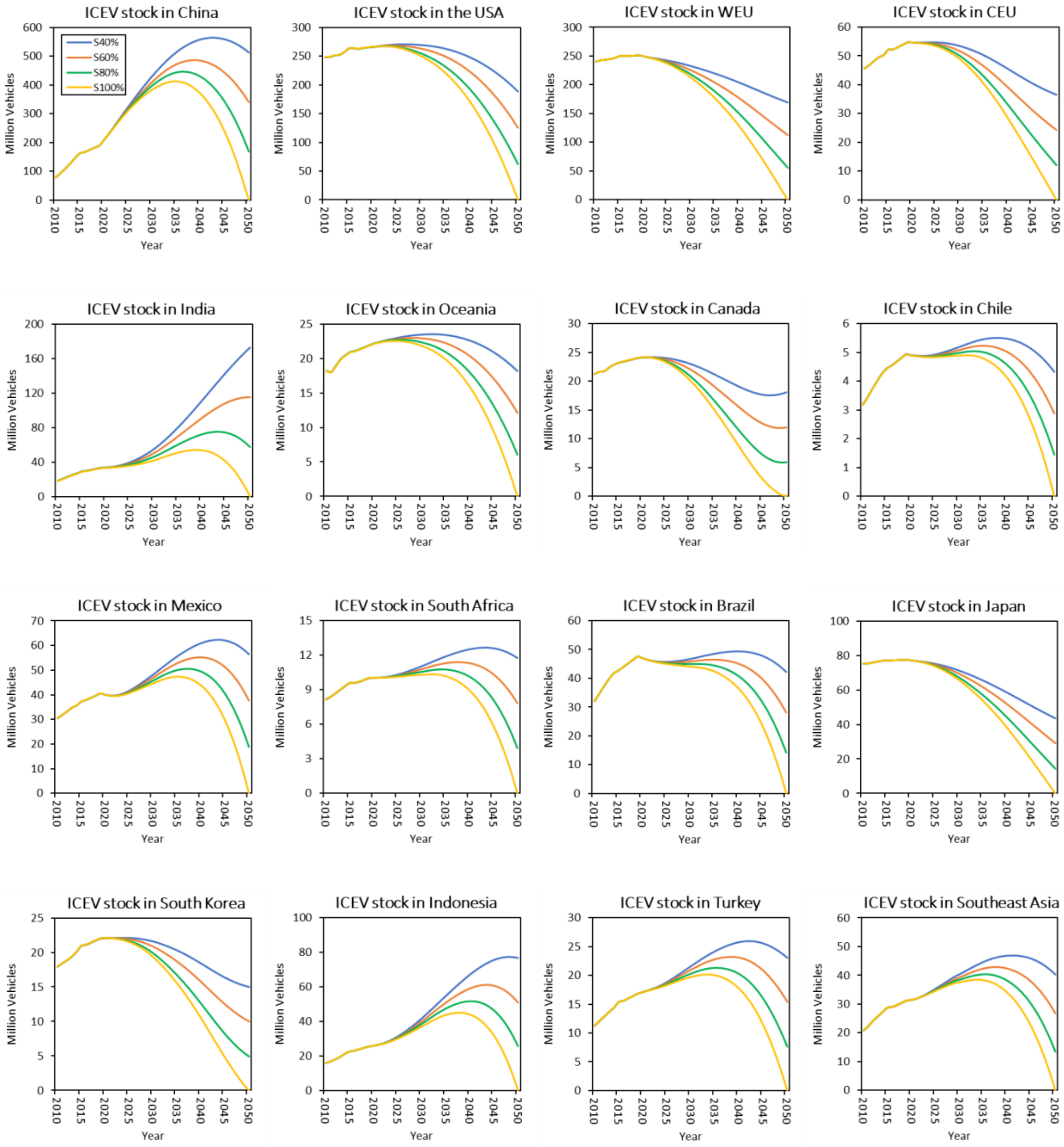

Figure S9. Stocks of internal combustion engine vehicles (ICEVs) in the target 16 regions under four EV penetration scenarios. Note: ICEVs include gasoline-based HEVs, diesel-based HEVs, gasoline ICEVs, diesel ICEVs, and natural gas ICEVs.

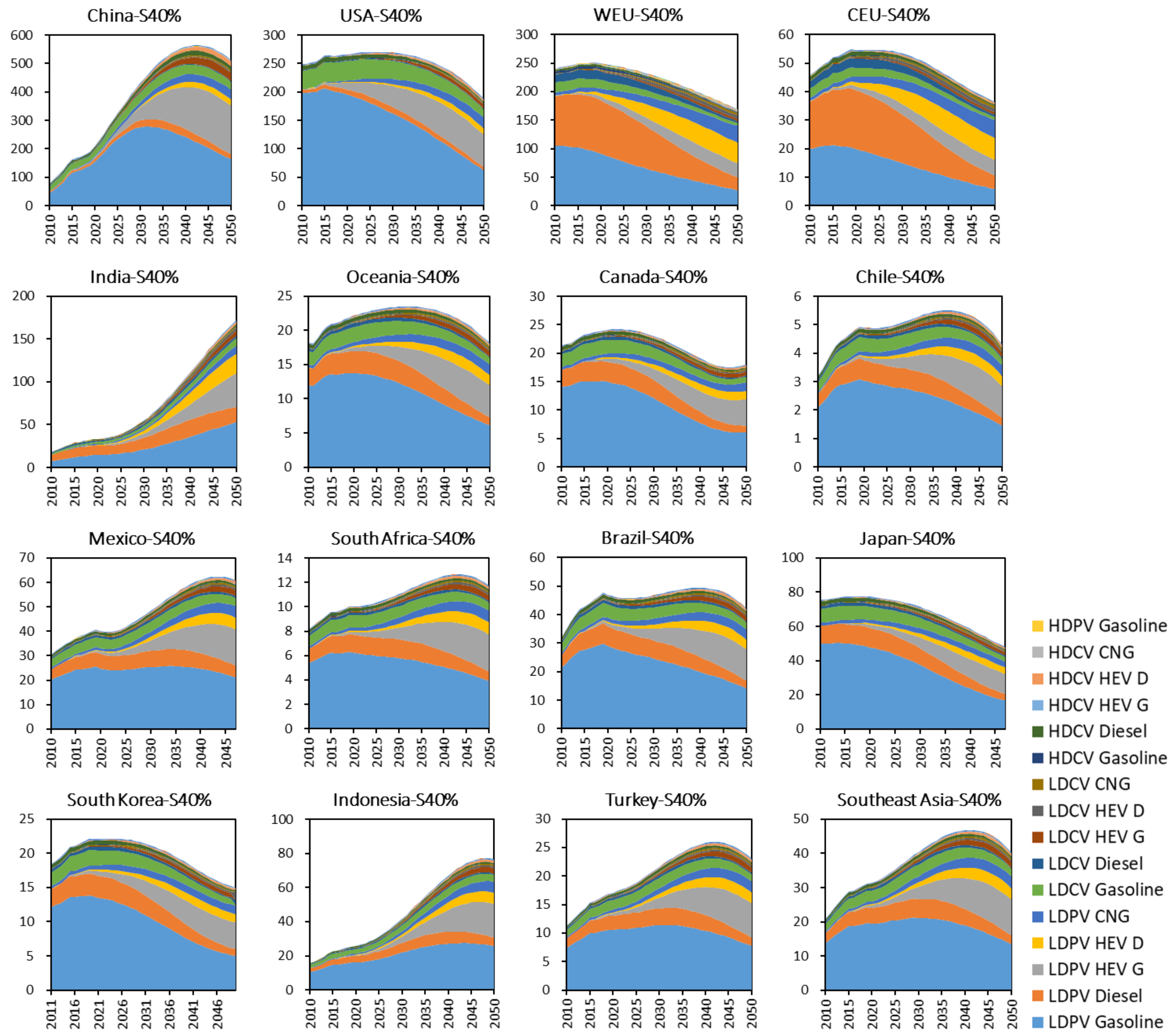

Figure S10. Transport mode-specific stocks (Unit: million vehicles) of internal combustion engine vehicles (ICEVs) in the target 16 regions under the S40% scenarios. Note: ICEVs include gasoline-based HEVs (HEV-G), diesel-based HEVs (HEV-D), gasoline ICEVs, diesel ICEVs, and natural gas ICEVs. LDPV: light-duty passenger vehicle, HDPV: heavy-duty passenger vehicle, LDCV: light-duty commercial vehicle, HDCV: heavy-duty commercial vehicle, CNG: condensed natural gas.

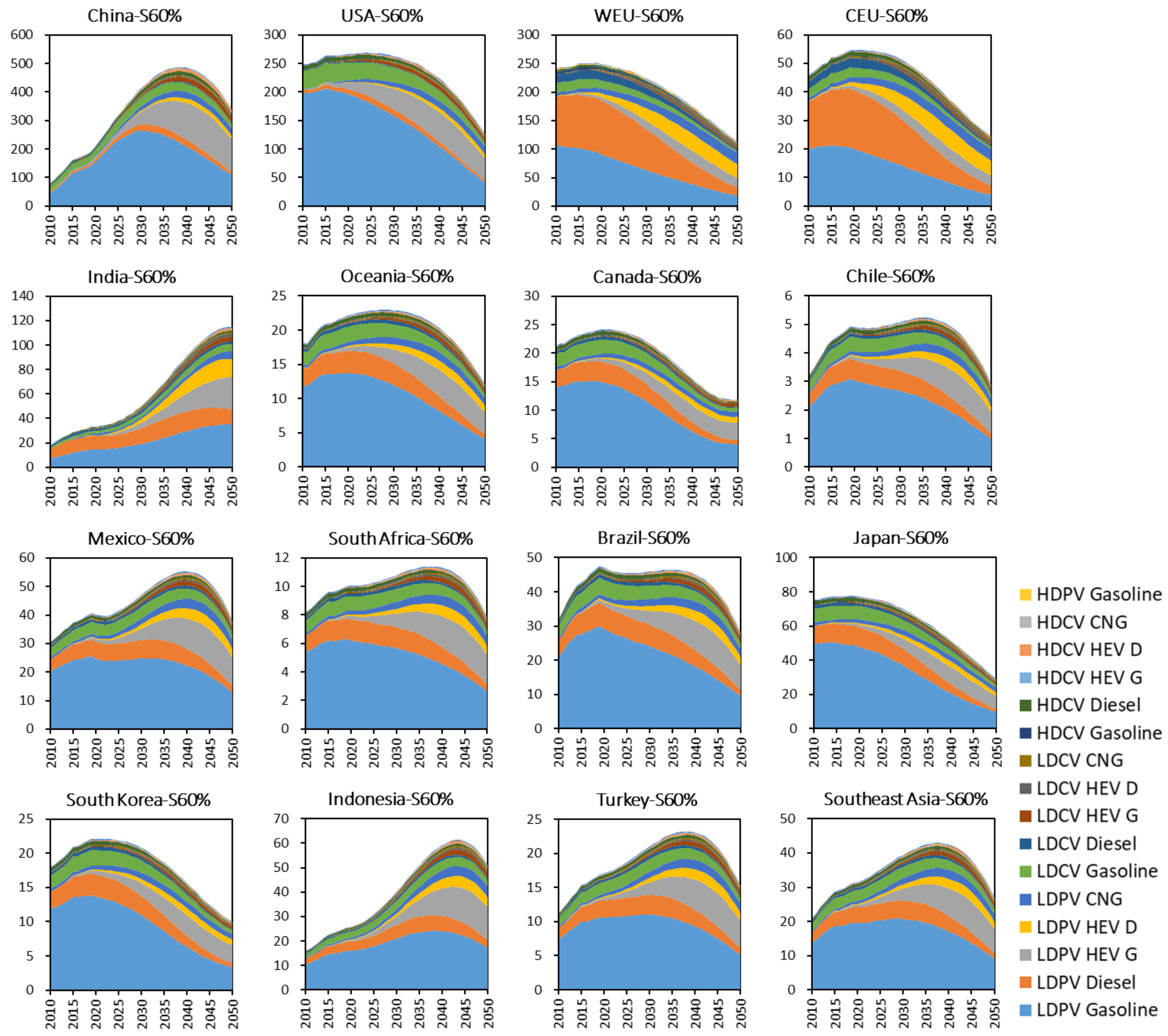

Figure S11. Transport mode-specific stocks (Unit: million vehicles) of internal combustion engine vehicles (ICEVs) in the target 16 regions under the S60% scenarios. Note: ICEVs include gasoline-based HEVs (HEV-G), diesel-based HEVs (HEV-D), gasoline ICEVs, diesel ICEVs, and natural gas ICEVs. LDPV: light-duty passenger vehicle, HDPV: heavy-duty passenger vehicle, LDCV: light-duty commercial vehicle, HDCV: heavy-duty commercial vehicle, CNG: condensed natural gas.

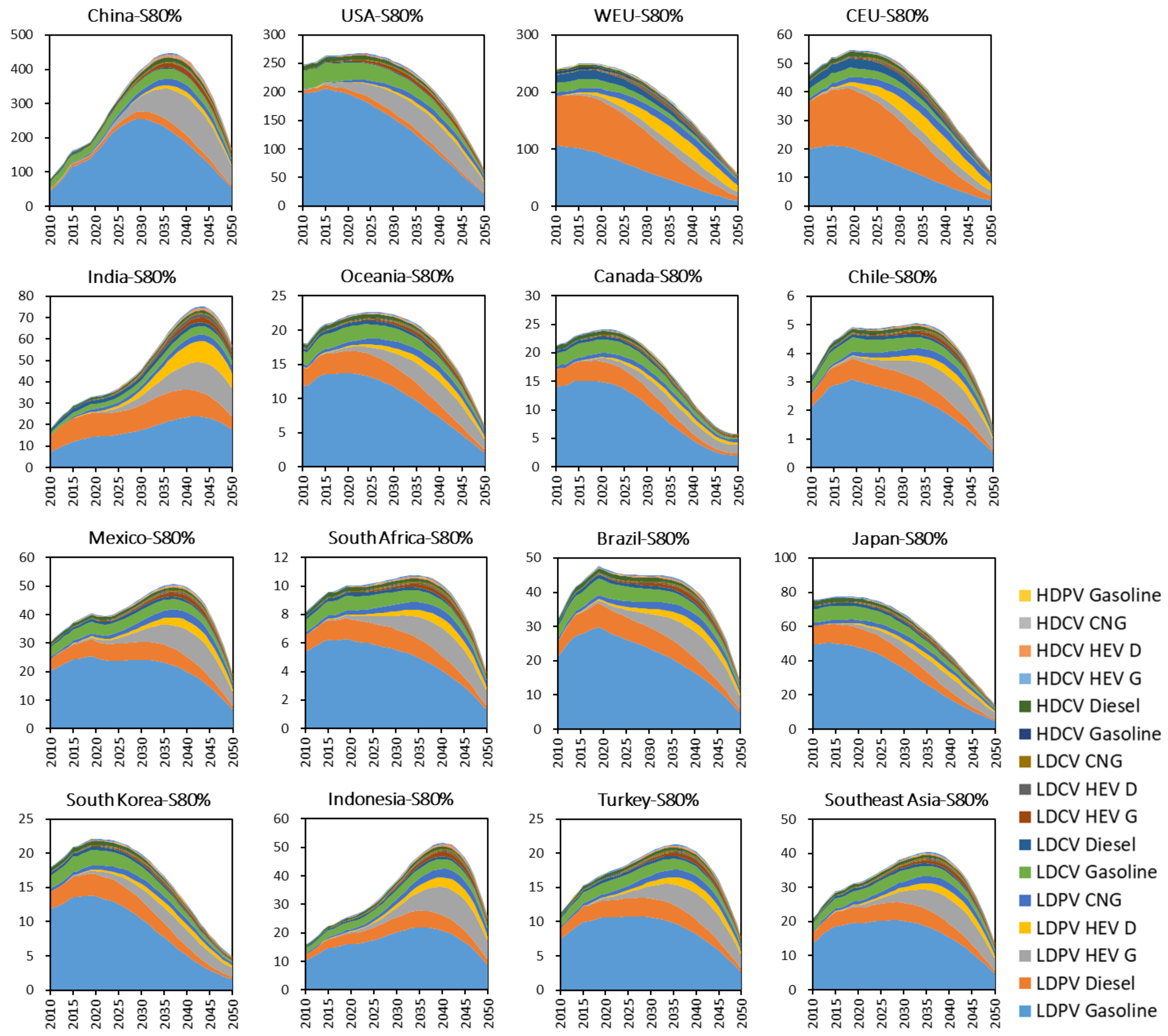

Figure S12. Transport mode-specific stocks (Unit: million vehicles) of internal combustion engine vehicles (ICEVs) in the target 16 regions under the S80% scenarios. Note: ICEVs include gasoline-based HEVs (HEV-G), diesel-based HEVs (HEV-D), gasoline ICEVs, diesel ICEVs, and natural gas ICEVs. LDPV: light-duty passenger vehicle, HDPV: heavy-duty passenger vehicle, LDCV: light-duty commercial vehicle, HDCV: heavy-duty commercial vehicle, CNG: condensed natural gas.

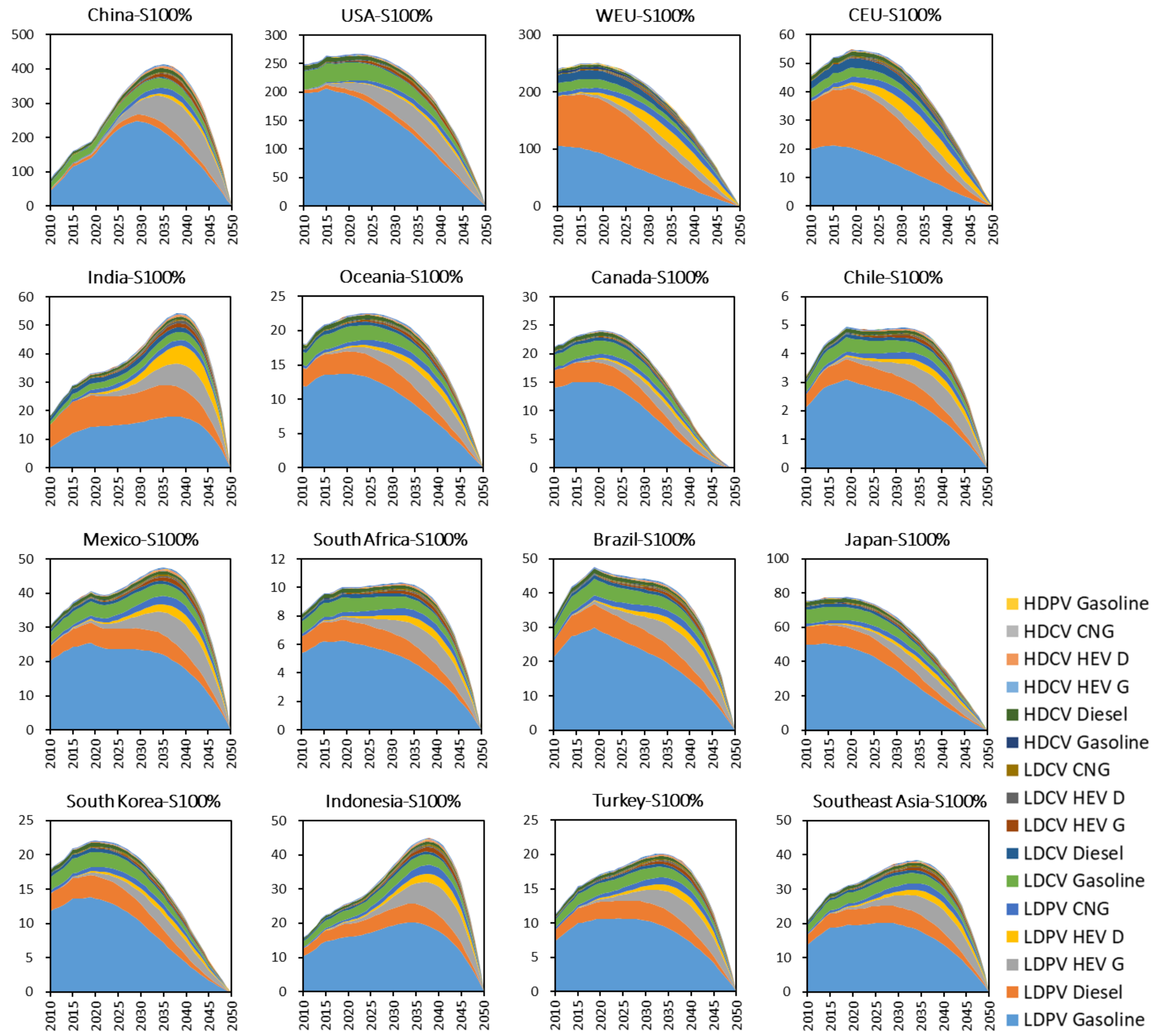

Figure S13. Transport mode-specific stocks (Unit: million vehicles) of internal combustion engine vehicles (ICEVs) in the target 16 regions under the S100% scenarios. Note: ICEVs include gasoline-based HEVs (HEV-G), diesel-based HEVs (HEV-D), gasoline ICEVs, diesel ICEVs, and natural gas ICEVs. LDPV: light-duty passenger vehicle, HDPV: heavy-duty passenger vehicle, LDCV: light-duty commercial vehicle, HDCV: heavy-duty commercial vehicle, CNG: condensed natural gas.

7. Sankey diagrams

The Sankey diagrams of the S40%–S100% scenarios are shown in Figure S14. Each Sankey diagram starts with a secondary production layer from the left side, followed by a primary production layer, a region layer, a vehicle layer, and a battery layer. The Sankey diagrams below show to which department cumulative (2010–2050) critical metals are used.

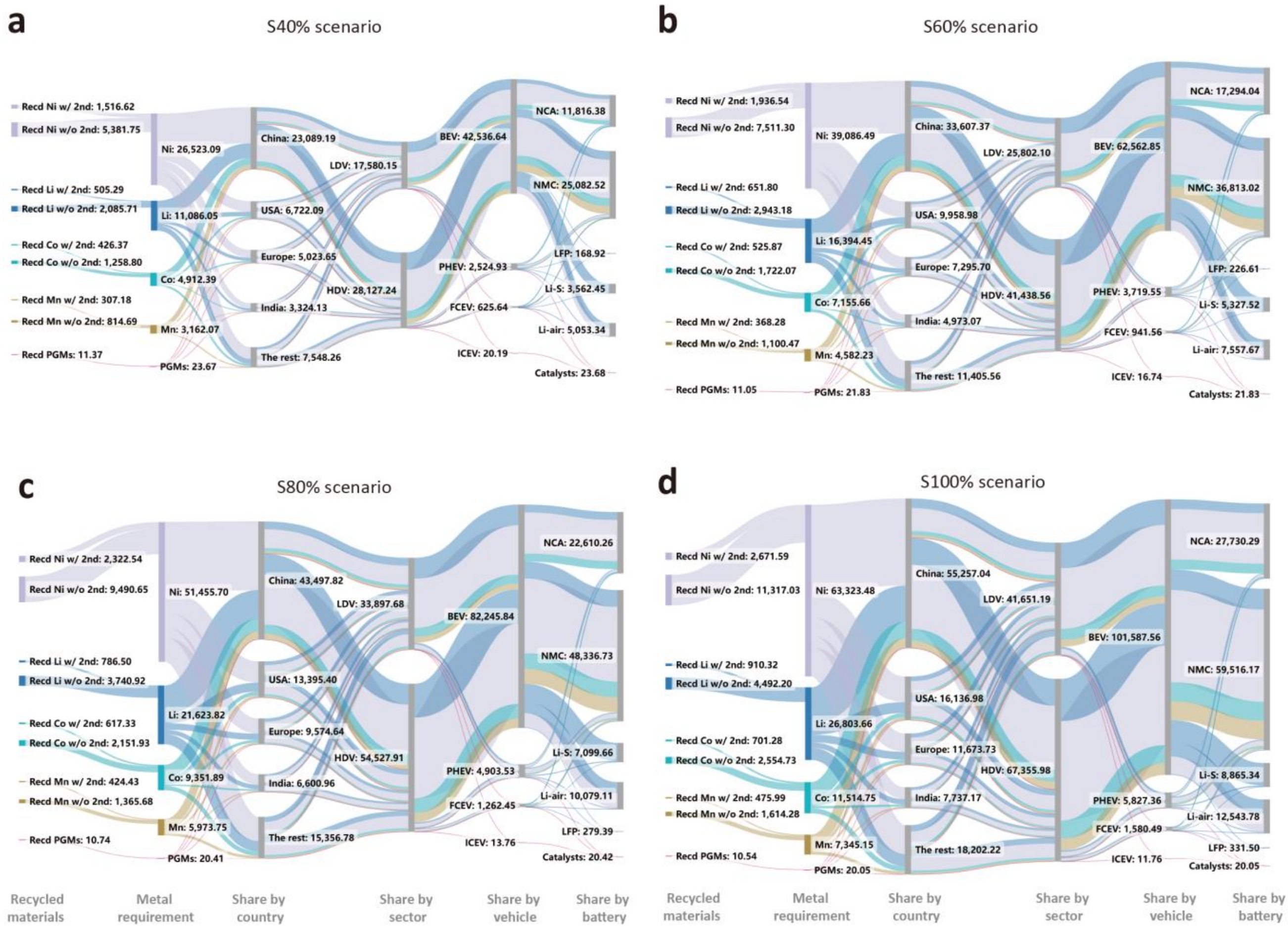

Figure S14. Sankey diagrams for cumulative critical metal requirements (Gg, also expressed as gigagram) under different S40% (a), S60% (b), S80% (c), and S100% (d) electric vehicle penetration scenarios. Note: The recycling nodes show the cumulative recycling potential of critical metals from either recycling with or without a second life. PGM: platinum group metal, Recd Li w/o 2nd: cumulative recycling potential of lithium without a second use, Recd Li w/ 2nd: cumulative recycling potential of lithium after second use, LDV: light-duty vehicle, HDV: heavy-duty vehicle, BEV: battery electric vehicle, PHEV: plug-in hybrid electric vehicle, FCEV: fuel cell electric vehicle, ICEV: internal combustion engine vehicle. LFP: lithium iron phosphate battery, NAC: lithium nickel cobalt aluminum battery, NMC: lithium nickel cobalt manganese battery, Li-S: lithium-sulfur battery, Li-air: lithium-air battery.

8. PGMs requirement for EVs and ICEVs

The requirement of platinum group metals (PGMs) for internal combustion engine vehicles (ICEVs) and electric vehicles (EVs) is shown in Figure S15.

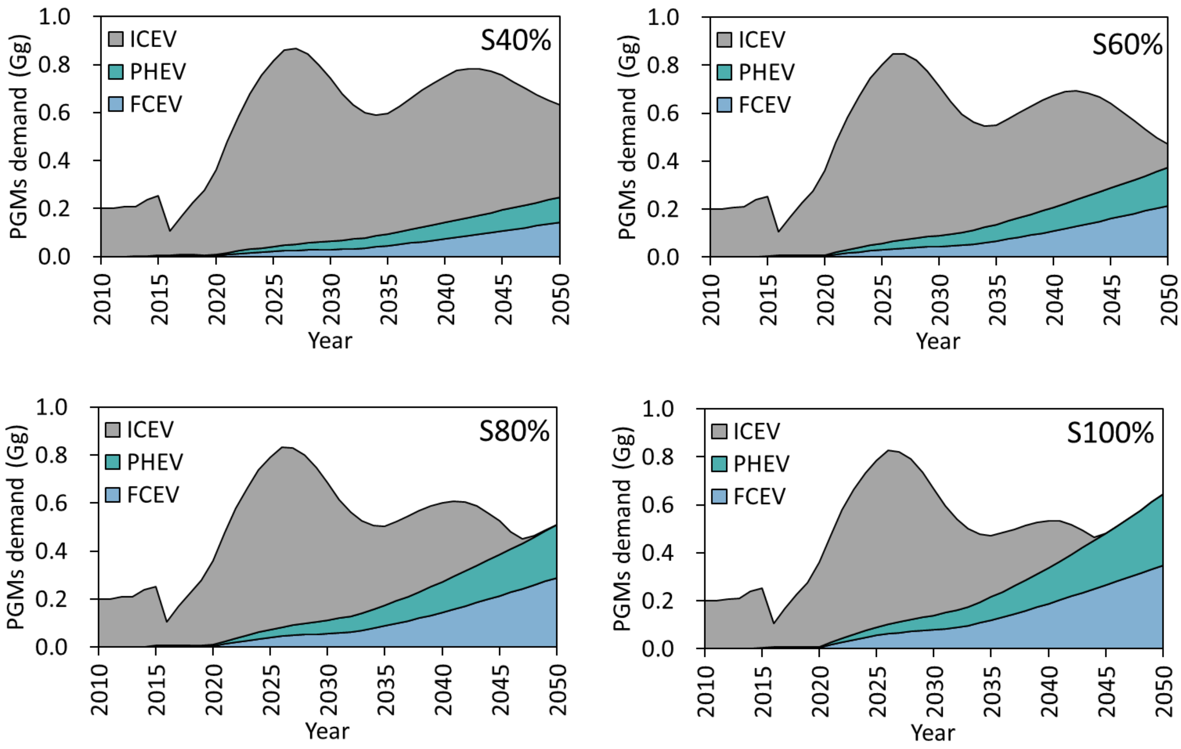

Figure S15 Platinum group metals (PGMs) requirement (Gg, also expressed as gigagram) for internal combustion engine vehicles (ICEVs) and electric vehicles (EVs). PHEV: plug-in hybrid electric vehicle, FCEV: fuel cell electric vehicle.

9. Sensitivity analysis for the future battery market

We established three extreme battery market scenarios—(i) NMC/NCA scenario, (ii) LFP scenario, and (iii) Li-air/Li-S scenario—to evaluate the sensitivity of the baseline battery scenario. It is assumed that the market shares of the NMC/NCA, LFP, and Li-air/Li-S battery will linearly increase to 100% under NMC/NCA, LFP, and Li-air/Li-S scenarios in 2050, respectively, as shown in Figure S16. The market share of NMC battery technologies in the four battery scenarios is shown in Figure S17.

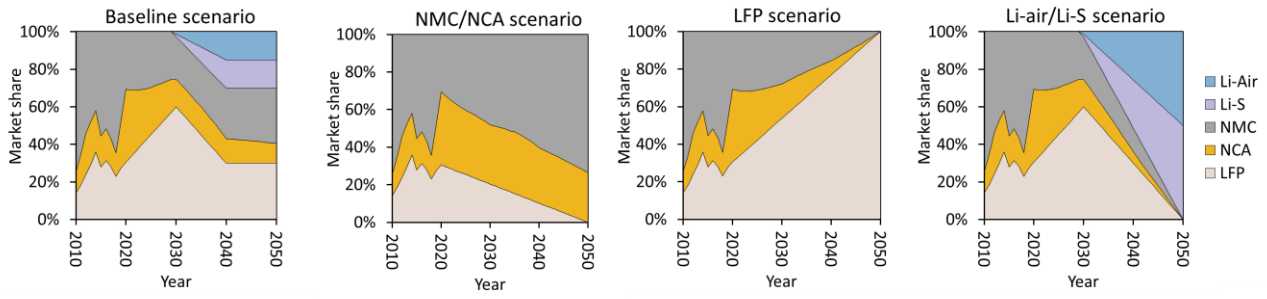

Figure S16 Different battery market scenarios for the sensitivity analysis of the critical metal requirement. LFP: Lithium iron phosphate battery, NCA: lithium nickel cobalt aluminum oxide battery, NMC: lithium nickel cobalt manganese oxide battery, Li-S: Lithium-sulfur battery, Li-air: Lithium-air battery.

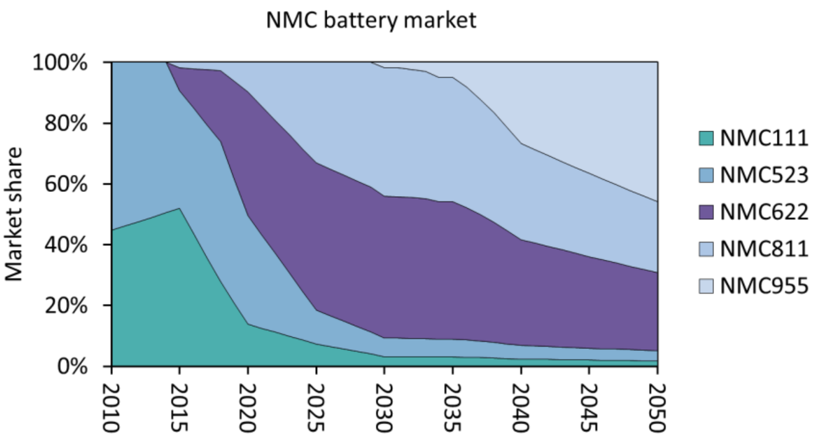

Figure S17 Battery market for NMC battery technology. LFP: Lithium iron phosphate battery, NCA: lithium nickel cobalt aluminum oxide battery, NMC: lithium nickel cobalt manganese oxide battery, Li-S: Lithium-sulfur battery, Li-Air: Lithium-air battery.

The annual critical metal requirement under four battery market scenarios is shown in Figure S18.

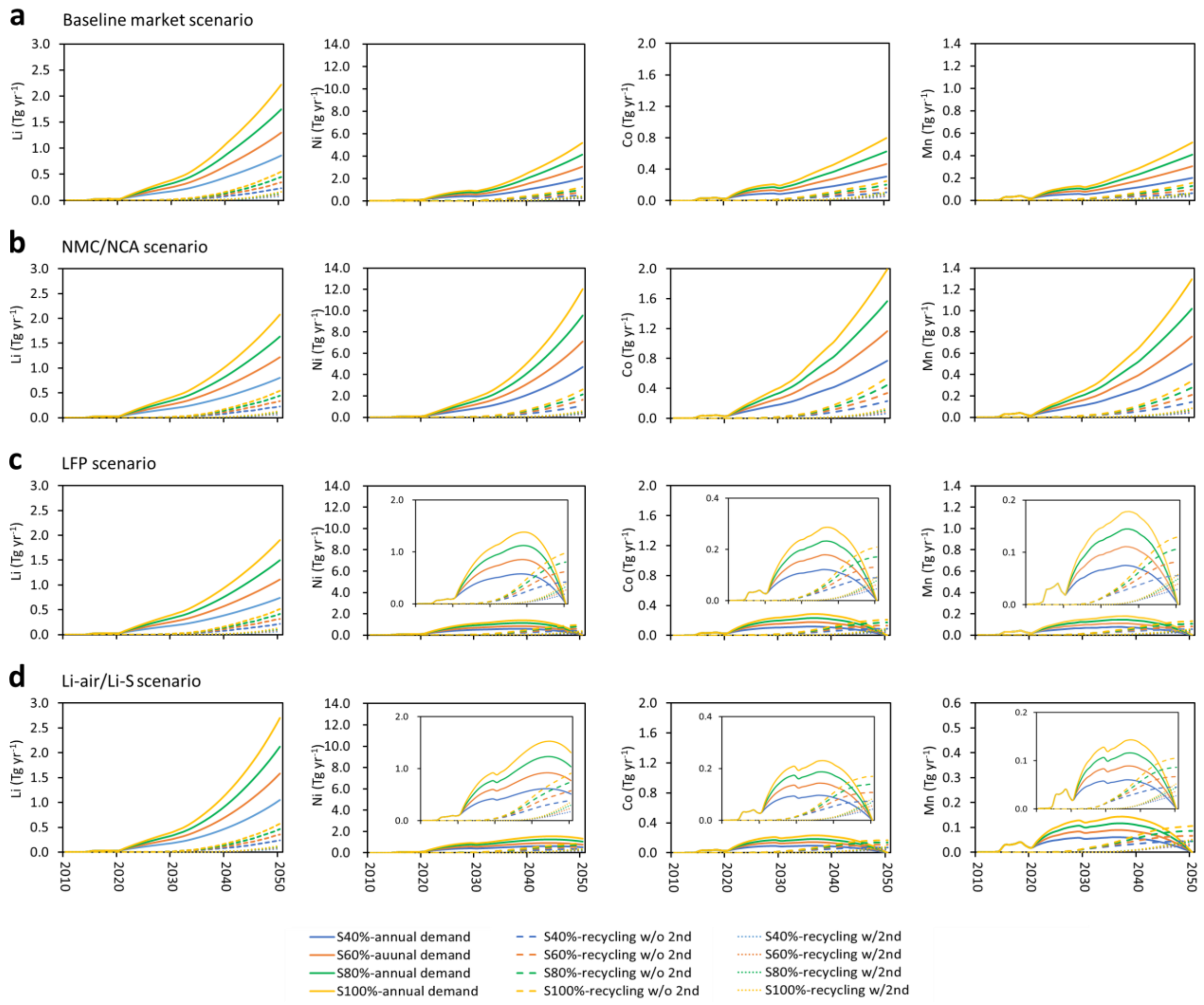

Figure S18 Sensitivity analysis of annual critical metal requirement (Tg, also expressed as teragram) under different battery market scenarios. LFP: Lithium iron phosphate battery, NCA: lithium nickel cobalt aluminum oxide battery, NMC: lithium nickel cobalt manganese oxide battery, Li-S: lithium-sulfur battery, Li-air: lithium-air battery.

Based on the annual critical metal requirement under different battery market scenarios in Figure S18 and the population in each region, the critical metal use per kWh of battery is shown in Figure S19.

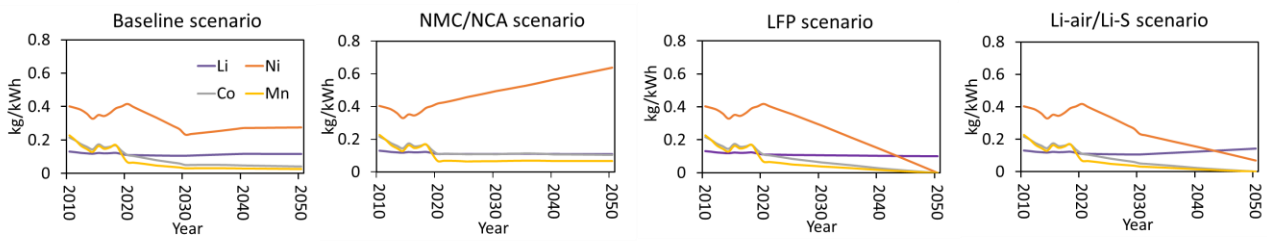

Figure S19 Market average of critical metal demand per kWh battery under four scenarios. LFP: lithium iron phosphate battery, NCA: lithium nickel cobalt aluminum oxide battery, NMC: lithium nickel cobalt manganese oxide battery, Li-S: lithium-sulfur battery, Li-air: lithium-air battery.

The cumulative material requirements of battery-related metals (lithium, nickel, cobalt, and manganese) under the NMC/NCA scenario, LFP scenario, and Li-S/air scenario are shown in Figure S20–Figure S22.

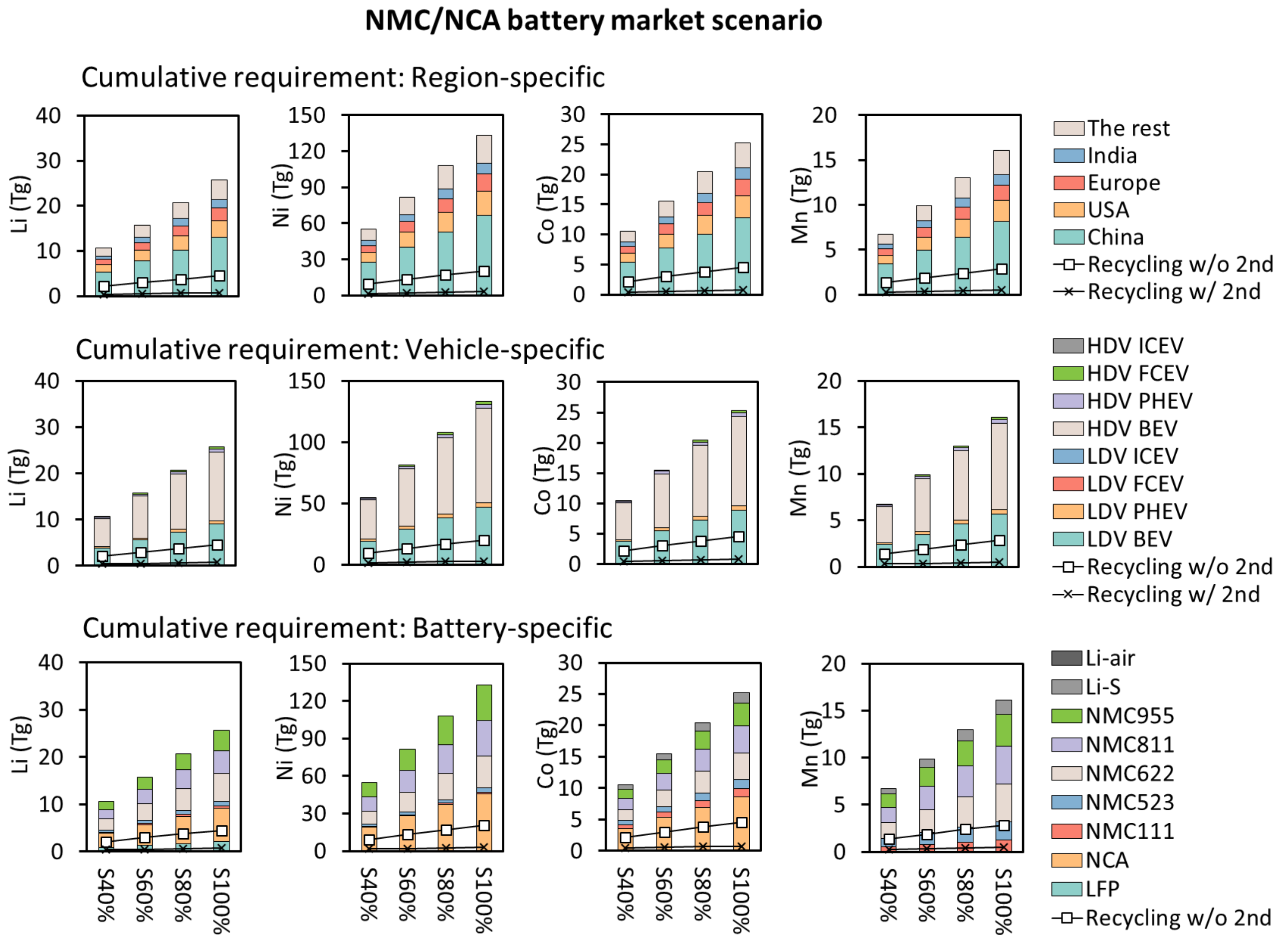

Figure S20 Cumulative material requirements (Tg, also expressed as teragram) of battery-related metals under the NMC/NCA scenario. "Recycling w/o 2nd" indicates retired batteries that are directly recycled without a second life as energy storage systems (ESSs). "Recycling w/2nd" denotes retired batteries reused as ESSs before recycling. LDV: light-duty vehicle, HDV: heavy-duty vehicle, BEV: battery electric vehicle, PHEV: plug-in hybrid electric vehicle, FCEV: fuel cell electric vehicle, ICEV: internal combustion engine vehicle. LFP: lithium iron phosphate battery, NCA: lithium nickel cobalt aluminum oxide battery, NMC: lithium nickel cobalt manganese oxide battery, Li-S: lithium-sulfur battery, Li-air: lithium-air battery.

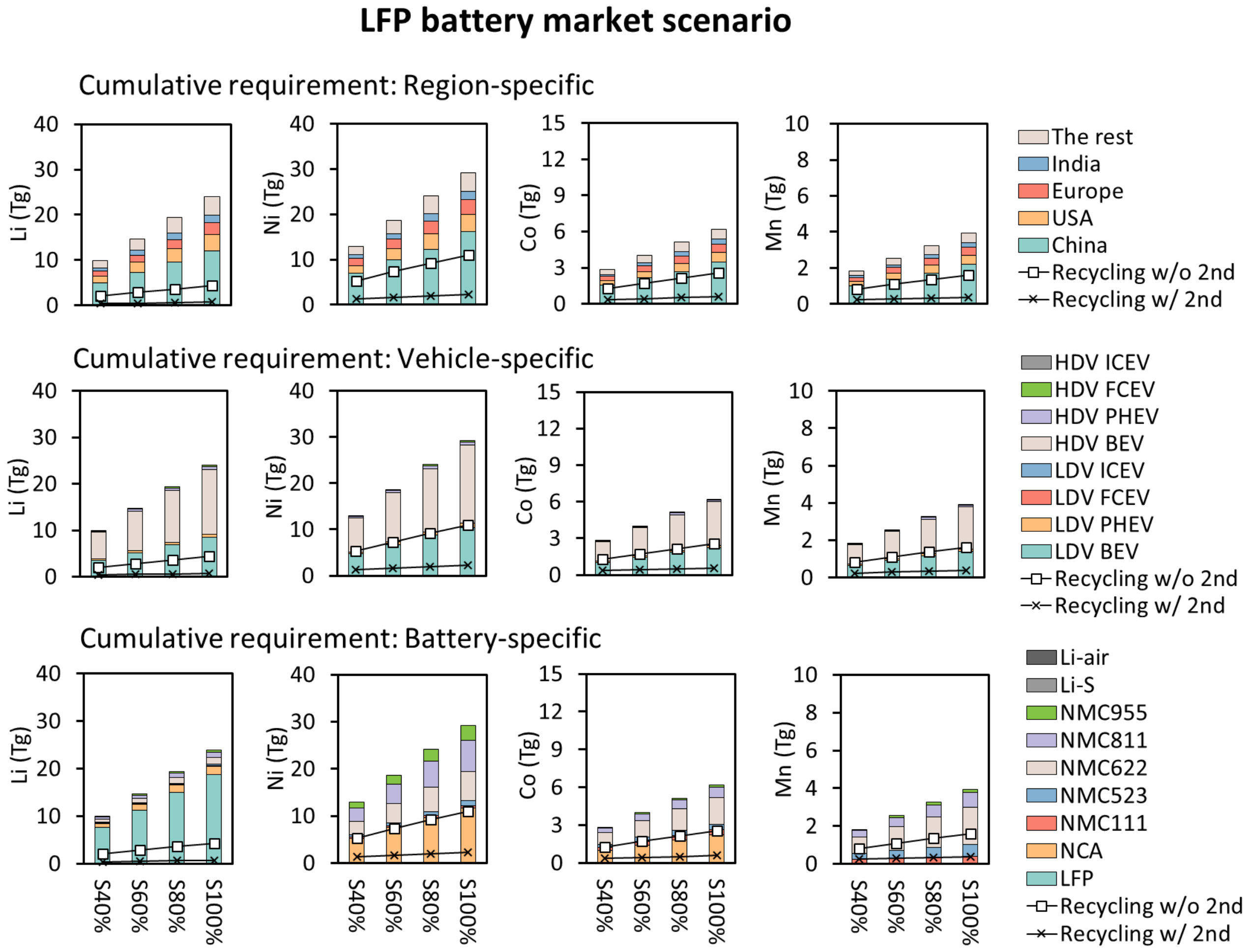

Figure S21 Cumulative material requirements (Tg, also expressed as teragram) of battery-related metals under the LFP scenario. "Recycling w/o 2nd" indicates retired batteries that are directly recycled without a second life as energy storage systems (ESSs). "Recycling w/2nd" denotes retired batteries reused as ESSs before recycling. LDV: light-duty vehicle, HDV: heavy-duty vehicle, BEV: battery electric vehicle, PHEV: plug-in hybrid electric vehicle, FCEV: fuel cell electric vehicle, ICEV: internal combustion engine vehicle. LFP: Lithium iron phosphate battery, NCA: lithium nickel cobalt aluminum oxide battery, NMC: lithium nickel cobalt manganese oxide battery, Li-S: lithium-sulfur battery, Li-air: lithium-air battery.

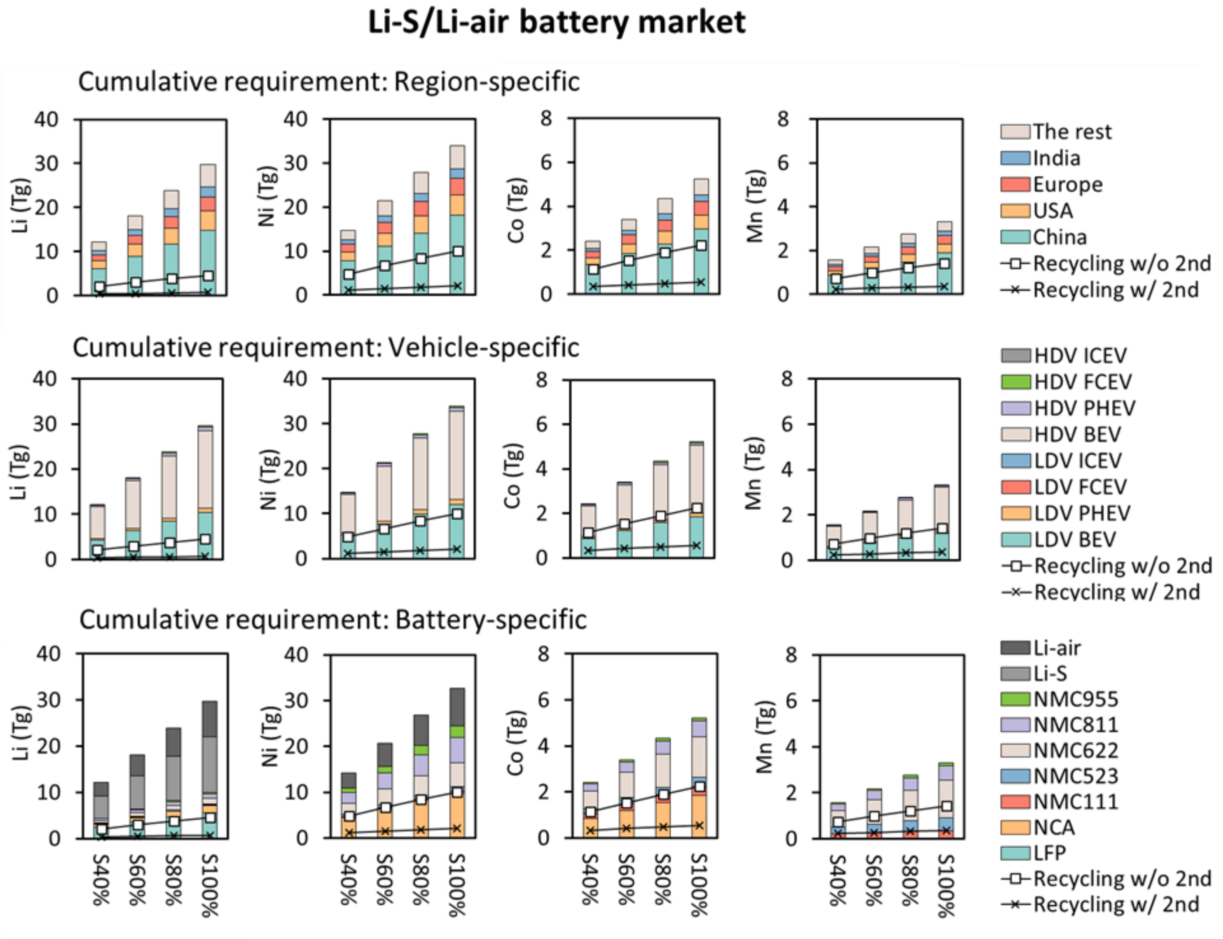

Figure S22 Cumulative material requirements (Tg, also expressed as teragram) of battery-related metals under the Li-S/Li-air scenario. "Recycling w/o 2nd" indicates retired batteries that are directly recycled without a second life as energy storage systems (ESSs). "Recycling w/2nd" denotes retired batteries reused as ESSs before recycling. LDV: light-duty vehicle, HDV: heavy-duty vehicle, BEV: battery electric vehicle, PHEV: plug-in hybrid electric vehicle, FCEV: fuel cell electric vehicle, ICEV: internal combustion engine vehicle. LFP: lithium iron phosphate battery, NCA: lithium nickel cobalt aluminum oxide battery, NMC: lithium nickel cobalt manganese oxide battery, Li-S: lithium-sulfur battery, Li-air: lithium-air battery.

10. Comparison of critical metal demand with resources and reserves

Figure S23a–e compares the annual demand and global gross production of each critical metal in 2020. Figure S23f–j compares the cumulative production and global reserves of each critical metal in 2020. The global lithium production in 2020 was estimated at 82.5 Gg, approximately 74% of which (61.1 Gg) was used for the battery industry. Australia is the primary lithium supplier and produced 39.7 Gg of lithium in 2020 (48% of the global production). While lithium production in China was around 13.3 Gg in 2020, the USA, Europe, and India are not major producers either. The global nickel production was around 2.5 Tg in 2020, and only 7% of that was used for the battery industry. China will contribute 43.4–47.1% to the gross nickel demand in 2050. Indonesia was the biggest producer, yielding 0.8 Tg (31%) of nickel in 2020. The nickel production in the two biggest nickel consumers, China and the USA, accounted for only 5% and 0.4%, respectively. Manganese production in 2020 was around 11 Tg and is sufficient for manufacturing EV batteries. In contrast, cobalt is more likely to encounter supply risks. The global cobalt production was just 0.1 Tg in 2020, and around 70% of production was from the Democratic Republic of the Congo. Approximately 40% of the global cobalt was supplied to the battery industry. The global PGM industry produced 0.4 Gg of PGMs in 2020, and 50% and 29% are from South Africa and Russia, respectively. Around 39% (approximately 0.1 Gg) of the production in 2020 was used in the vehicle sector. It is worth noting that reserves of the five critical metals have remained relatively stable over the last two decades[39], while reserves may undergo major volatilities in the future based on new geological discoveries, technological advancements, etc.

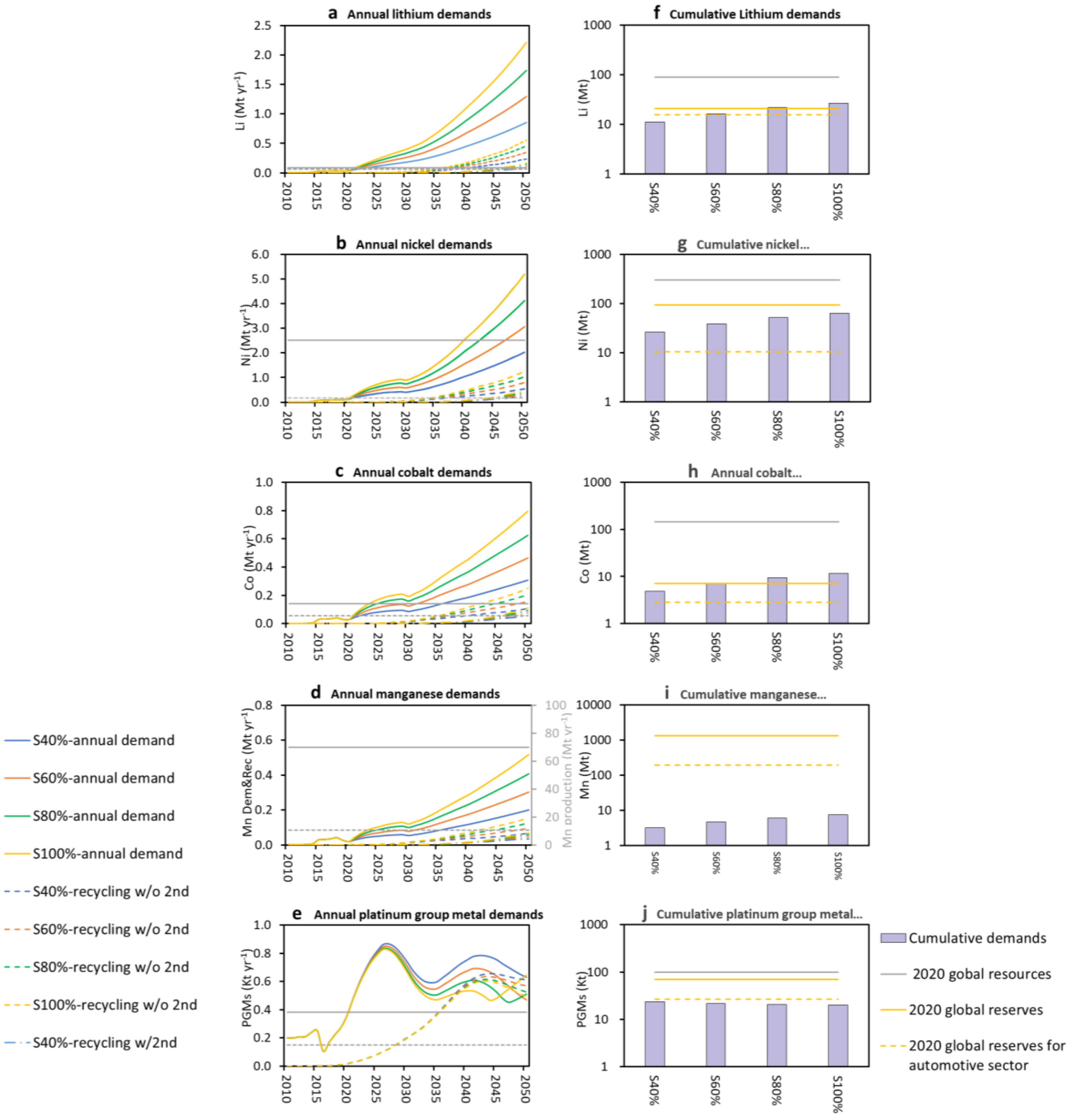

Figure S23 Comparison of annual critical metal demands and annual production (a-e), and comparison of cumulative critical metal demands and reserves and resources (f-j). Note: Tg means teragram, and Gg denotes gigagram. “Recycling w/o 2nd” indicates retired batteries are directly recycled without a second life as energy storage systems (ESS). “Recycling w/2nd” denotes retired batteries are used with a second life as ESS before recycling. Note: the global resources and reserves data were collected from USGS[39]. The end-usage shares of lithium (74%) and cobalt (40%) production and reserves used for the LIB industry are assumed based on the data from Statista[40,41]. The end-usage share of nickel (11%) production and reserves used for the LIB industry are assumed based on the data from Nickel Institute[42]. The end-usage share of manganese (15%) production and reserves used for the

LIB industry is assumed based on the data from Mordor Intelligence via https://www.mordorintelligence.com/industry-reports/manganese-market. The end-usage share of platinum group metals (36–42%) production and reserves used for the automotive sector is assumed based on the data from Mordor Intelligence via CME Group[43]. The recycling rates for lithium, nickel, cobalt, and manganese are assumed based on the World Bank[33]. The recycling rate for PGMs is assumed based on the study[11].

Figure S24 shows the production and reserve share of each critical metal in 2020. The data was collected from the United States Geological Survey (USGS)[39]. Australia (40 Gg), the Democratic Republic of the Congo (Congo) (95 Gg), and Indonesia (760 Gg) were the largest producers of lithium, cobalt, and nickel worldwide in 2020, respectively. South Africa produced the most manganese (5,200 Gg) and PGMs (190 tons) in 2020. Regarding the reserves, Chile and Congo owned the largest lithium and cobalt reserve in 2020, amounting to 9.2 and 3.6 Tg, respectively. For nickel, the largest three reserves in 2020 were located in Indonesia (21 Tg), Argentina (20 Tg), and Brazil (16 Tg). South Africa had the largest manganese and PGMs reserves in 2020, 520 Tg and 63 Gg, respectively.

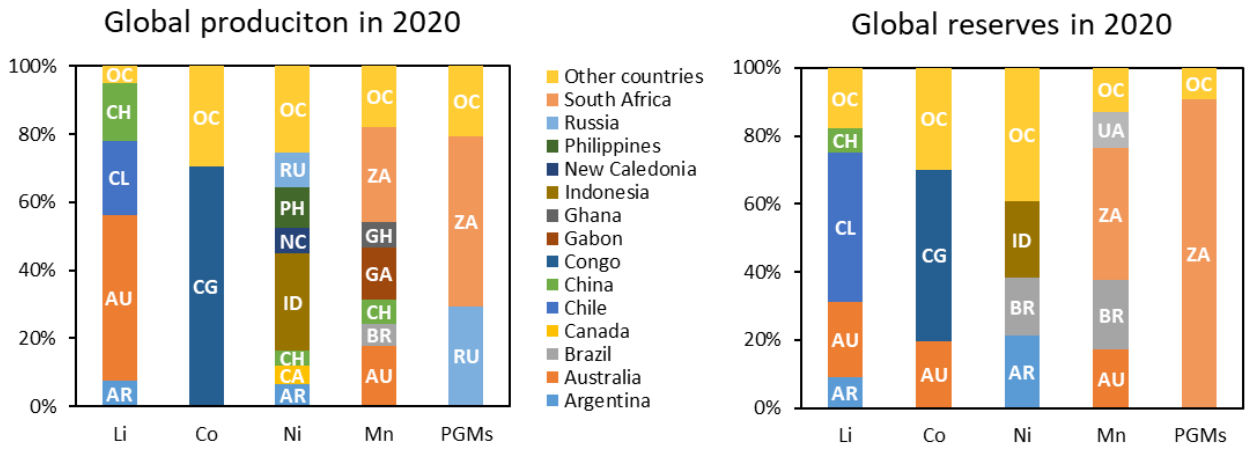

Figure S24 Global share production and reserves of lithium, cobalt, nickel, manganese, and platinum group metals in 2020. Note: data from USGS[39].

11. Annual greenhouse gas emissions

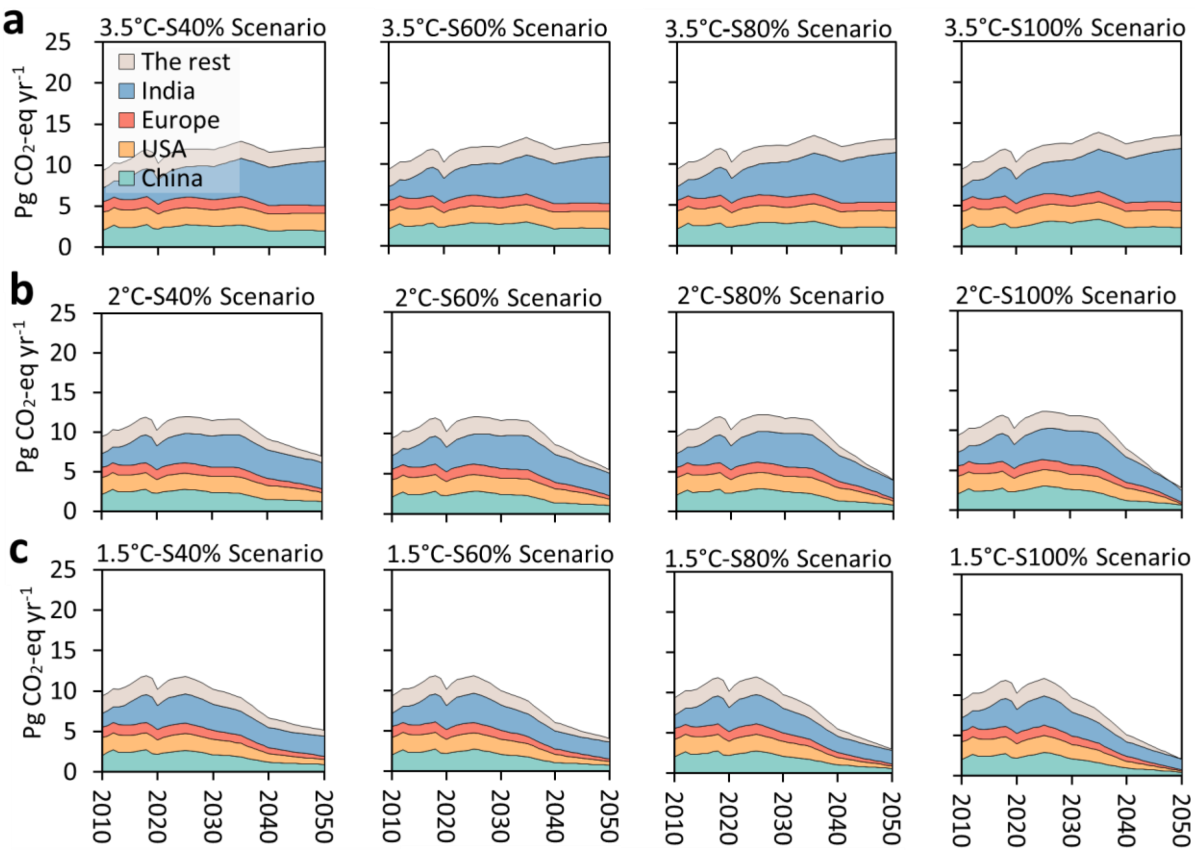

Figure S25 Annual greenhouse gas (GHG) emissions (Pg, also expressed as petagram) based on country-wise breakdown. a Annual GHG emissions from fuel for road transportation across four EV penetration levels (40–100%) under the 3.5℃ (IMAGE 3.2 SSP2-RCP6) energy transition scenario. The results for 2010–2020 are historical statistics; the results for 2021–2050 are projected. **b** Annual GHG emissions from fuel for road transportation under the 2℃ (IMAGE 3.2 SSP2-RCP26) energy transition scenario. **c** Annual GHG emissions from fuel for road transportation under the 1.5℃ (IMAGE 3.2 SSP2-RCP19) energy transition scenario.

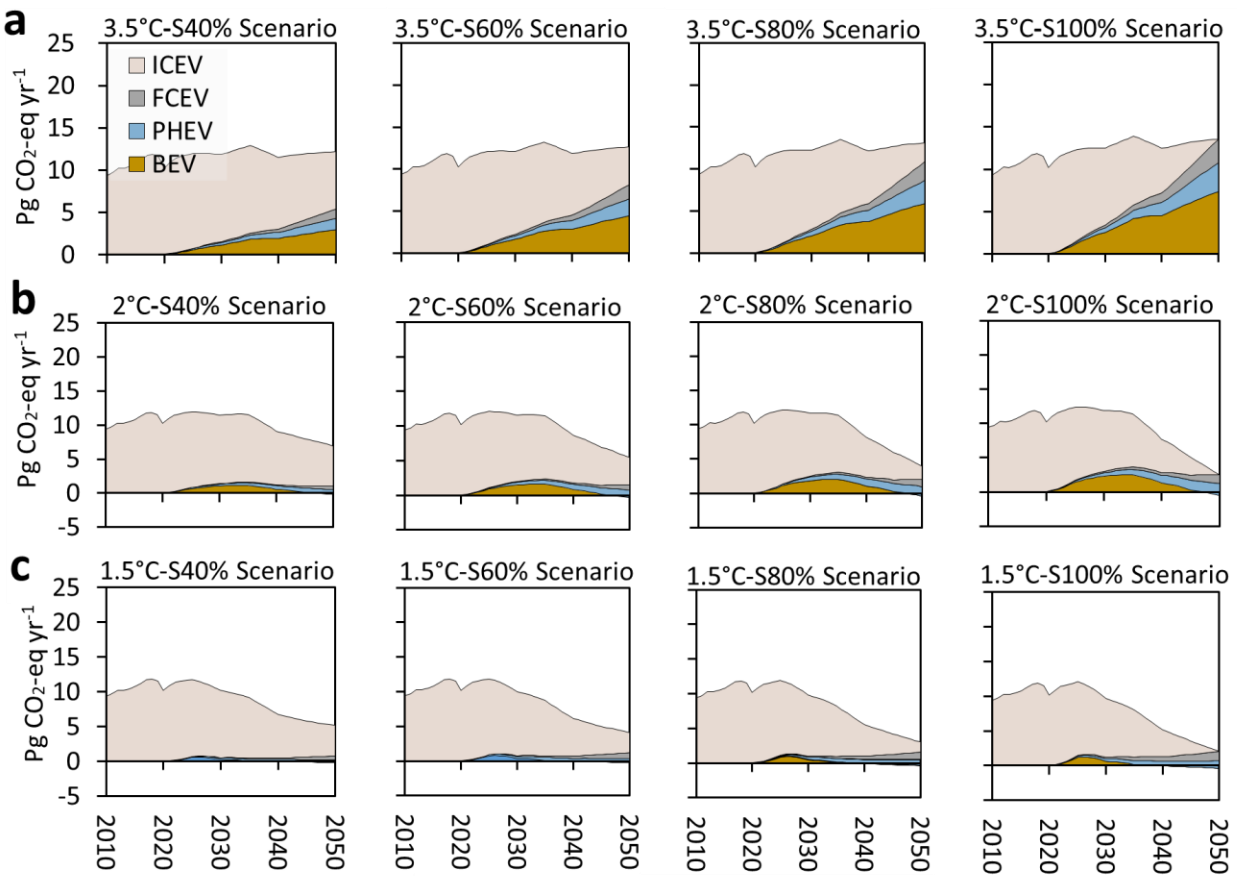

Figure S26 Annual greenhouse gas (GHG) emissions (Pg, also expressed as petagram) based on powertrain-wise breakdown. a Annual GHG emissions from fuel for road transportation across four EV penetration levels (40–100%) under the 3.5℃ (IMAGE 3.2 SSP2-RCP6) energy transition scenario. The results for 2010–2020 are historical statistics; the results for 2021–2050 are projected. **b** Annual GHG emissions from fuel for road transportation under the 2℃ (IMAGE 3.2 SSP2-RCP26) energy transition scenario. **c** Annual GHG emissions from fuel for road transportation under the 1.5℃ (IMAGE 3.2 SSP2-RCP19) energy transition scenario. BEV: battery electric vehicle, PHEV: plug-in hybrid electric vehicle, FCEV: fuel cell electric vehicle, ICEV: internal combustion engine vehicle.

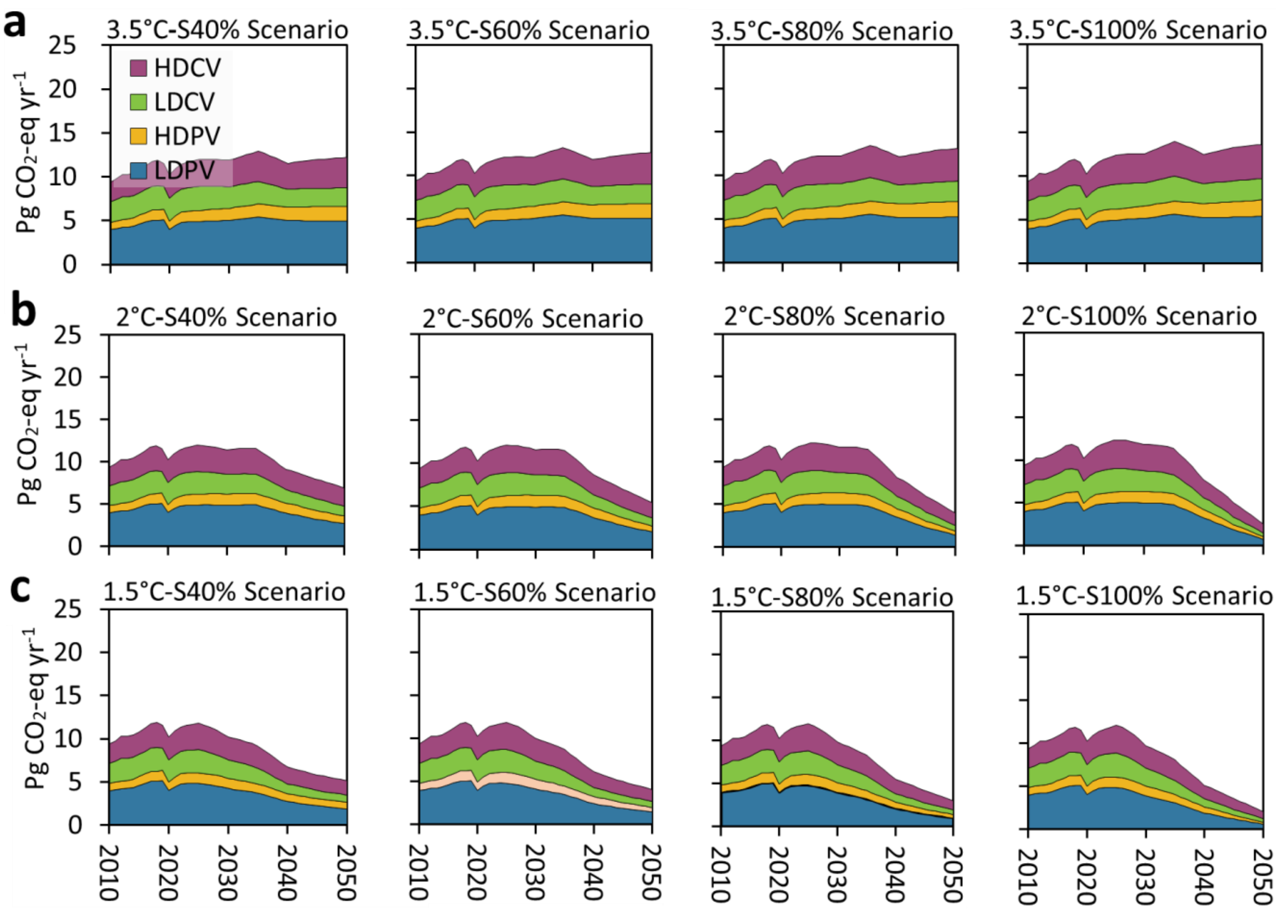

Figure S27 Annual greenhouse gas (GHG) emissions (**Pg, also expressed as petagram**) **based on sector-wise breakdown. a** Annual GHG emissions from fuel for road transportation across four EV penetration levels (40–100%) under the 3.5℃ (IMAGE 3.2 SSP2-RCP6) energy transition scenario. The results for 2010–2020 are historical statistics; the results for 2021–2050 are projected. **b** Annual GHG emissions from fuel for road transportation under the 2℃ (IMAGE 3.2 SSP2-RCP26) energy transition scenario. **c** Annual GHG emissions from fuel for road transportation under the 1.5℃ (IMAGE 3.2 SSP2-RCP19) energy transition scenario. LDPV: light-duty passenger vehicle, HDPV: heavy-duty passenger vehicle, LDCV: light-duty commercial vehicle, HDCV: heavy-duty commercial vehicle.

12. Fuel emission intensity

Fuel production emissions

Figure S28 shows the carbon footprint per kWh of electricity and per kg of fuel in 16 regions. Those emission indicators were estimated based on the PREMISE v1.2.6 database[32] and IMAGE 3.2 database[1].

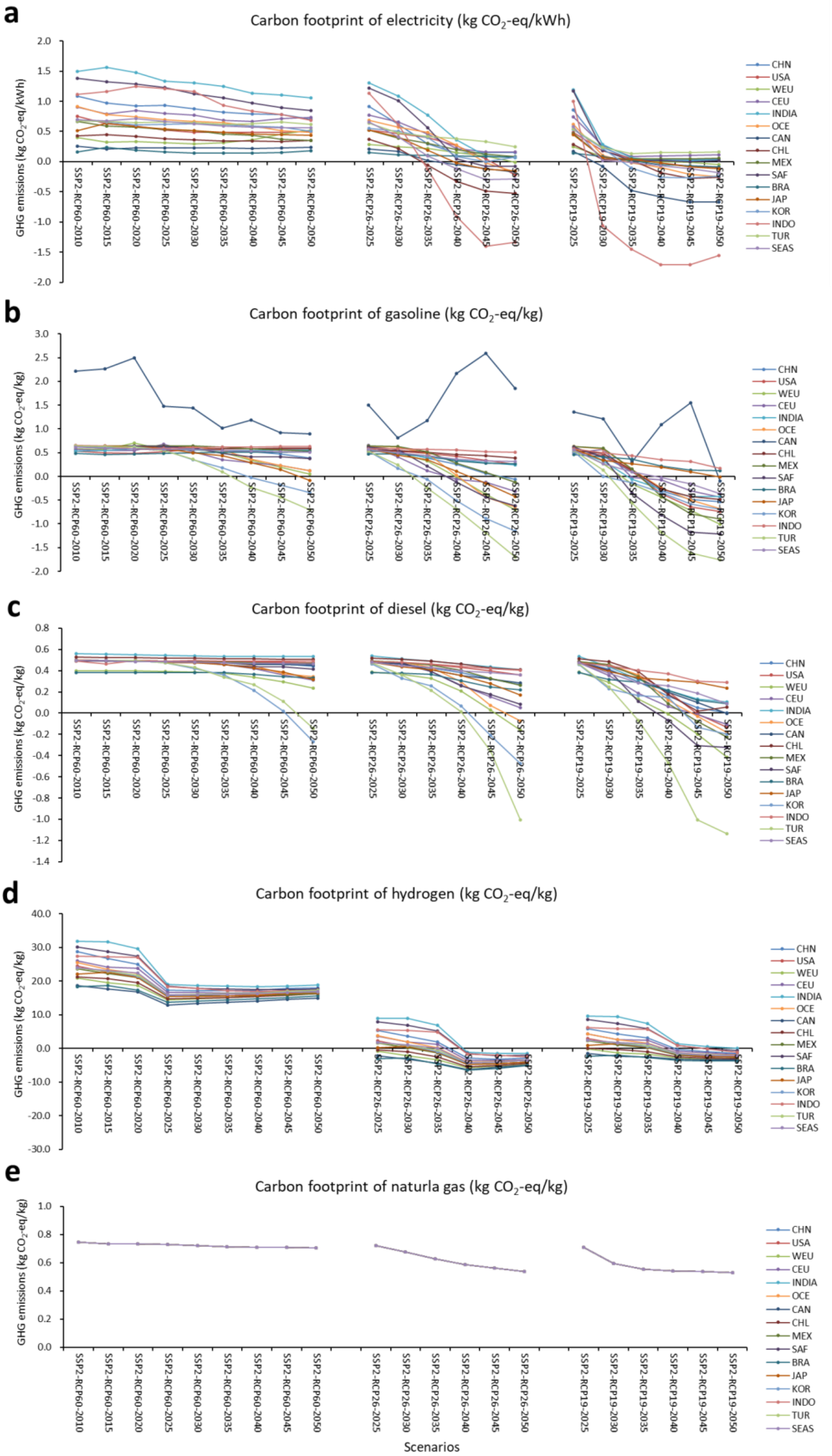

Figure S28. Carbon footprint per kWh (kilowatt hour) of electricity and per kg (kilogram) of fuel in 16 regions under three energy transition scenarios. OCE: Oceania, WEU: Western Europe, CEU:

Central Europe, SEAS: Southeast Asia, SAF: South Africa, RSAM: Rest of south America, TUK: Turkey, MEX: Mexico, INDIA: India; JAP: Japan, CHN: China, CAN: Canada, KOR: South Korea, INDO: Indonesia region, USA: the United States, and BRA: Brazil.

Biofuels blended in gasoline and diesel

The Share (by weight) of the bioethanol blended in gasoline in 16 regions under three energy transition scenarios is shown in Figure S29.

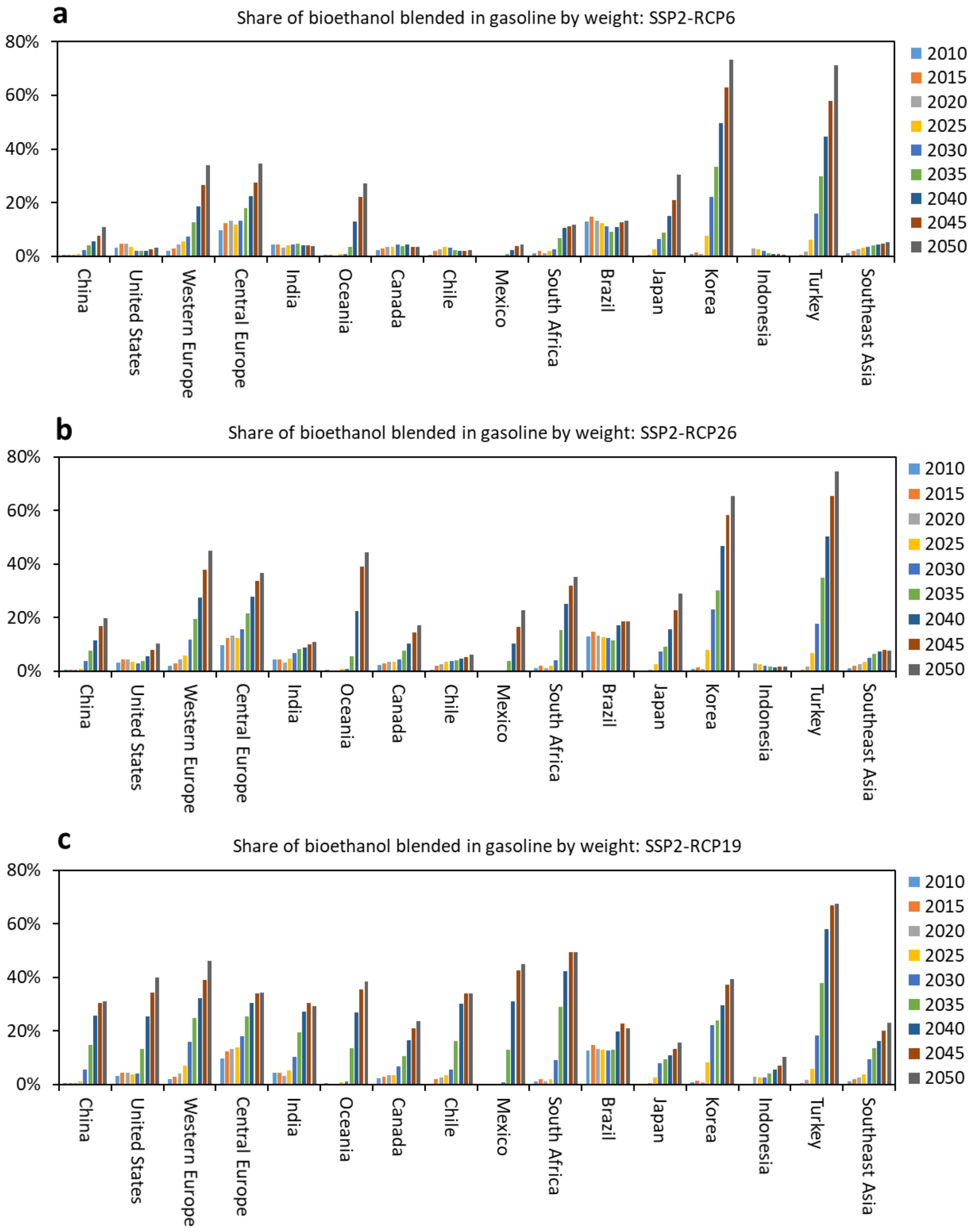

Figure S29. Share of bioethanol blended in gasoline by weight in 16 regions under three energy transition scenarios

The Share (by weight) of the biodiesel blended in diesel in 16 regions under three energy transition scenarios is shown in Figure S30.

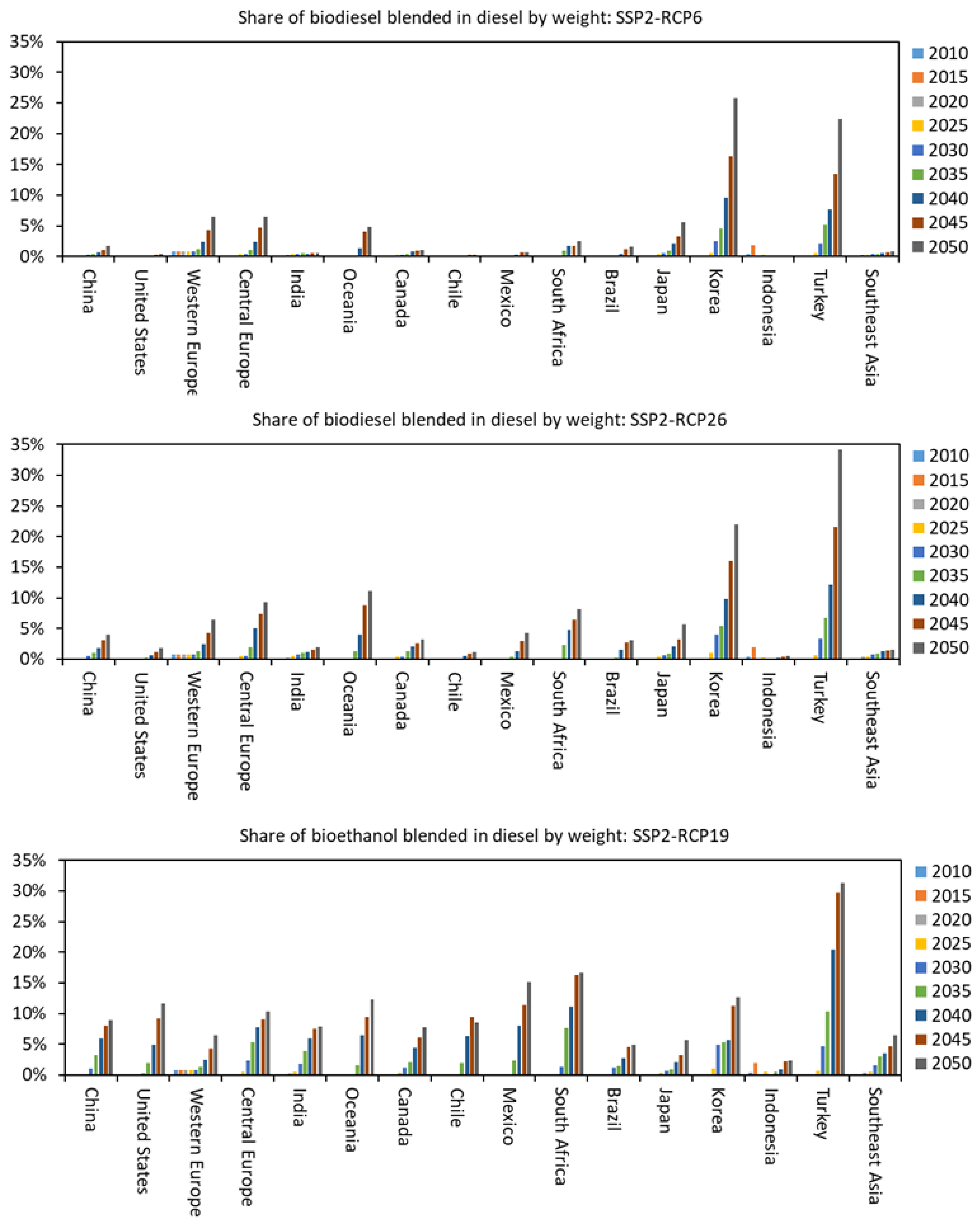

Figure S30 Share of biodiesel blended in diesel by weight in 16 regions under three energy transition scenarios

The premise v1.2.6 database[32] does not include the market share of hydrogen production approaches. The projected global hydrogen production market data from IMAGE 3.2 was used to represent the hydrogen production market in each region, as shown in Figure S31. From 2010 to 2020, over 80% of the hydrogen was produced based on fossil fuels such as coal gasification and methane auto-thermal reforming (ATM). Under the 3.5% scenario, grey hydrogen dominates the global hydrogen production market; and steam methane reforming (SMR) will dominate the hydrogen market since 2025 (80%) and will slowly decrease to 60% in 2050. In the more ambitious 2°C and 1.5°C scenarios, CCS technologies will be employed for coal gasification, ATM, and SMR for blue hydrogen production. The green hydrogen produced from water electrolysis and solar thermal is less than 1%, thus does not consider.

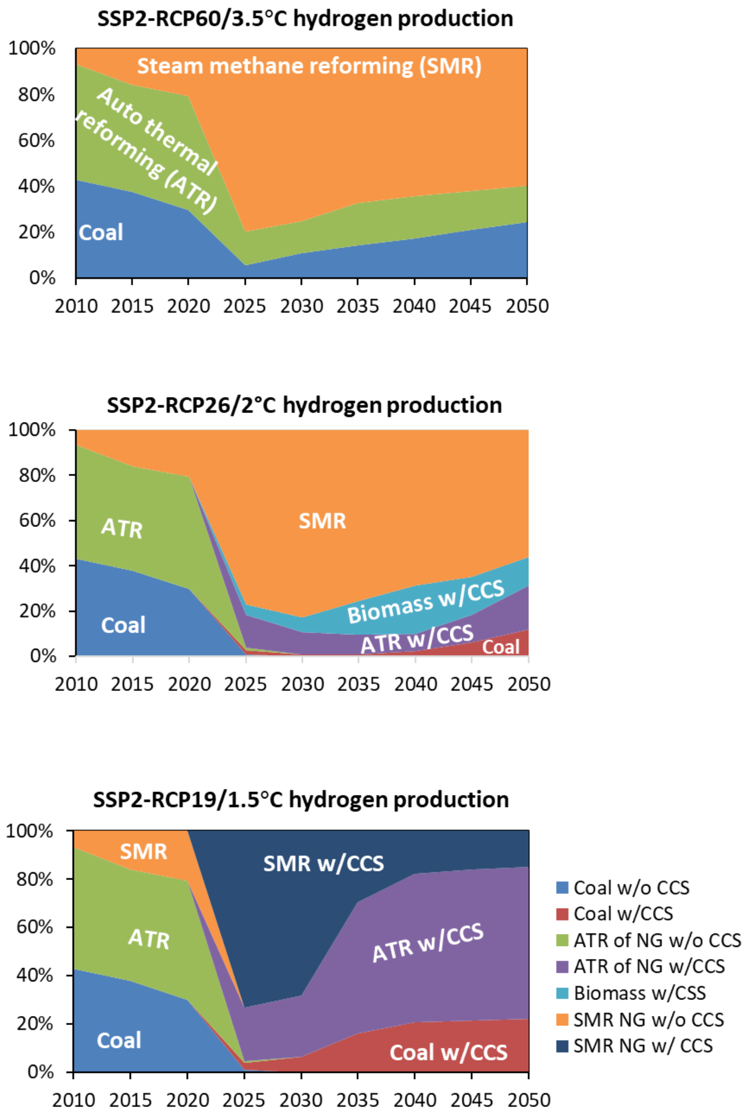

Figure S31 Hydrogen production market in the three energy transition scenarios.

Fuel use emissions

The fuel emission factors of fuel are based on the European Union European Environment Agency's (EEA) Air pollutant emission inventory guidebook 2019[44]. Emission factors for CO_2, CO, and N_2O different road transport fuels are listed in Table S2 to Table S4.

Table S2 CO_2 emission factor for different road transport fuels

Vehicle category	Fuel	Kg CO_2 per kg(/kWh) fuel
All vehicle types	Petrol	3.169
	Diesel	3.169
	Natural gas	3.024
	Electricity	0.000

Table S3 CO emission factor for different road transport fuels. Note: mean value

Vehicle category	Fuel	g CO per kg(/kWh) fuel
Passenger car	Petrol	84.70
	Diesel	3.33
	Electricity	0.00
Light-duty commercial vehicle	Petrol	152.3
	Diesel	7.40
	Electricity	0.00
Heavy-duty commercial vehicle	Diesel	7.58
	Natural gas	5.70
	Electricity	0.00

Table S4 N_2O emission factor for different road transport fuels. Note: mean value

Vehicle category	Fuel	g N_2O per kg(/kWh) fuel
Passenger car	Petrol	0.206
	Diesel	0.087
	Electricity	0.000
Light-duty commercial vehicle	Petrol	0.186
	Diesel	0.056
	Electricity	0.000
Heavy-duty commercial vehicle	Diesel	0.051

Regarding global warming (GWP) potential characterization factors for CO_2, CO, and N_2O are derived from IPCC's Fifth Assessment Report[45], shown as in Table S5. For H_2 supplied to FCEVs, 1.5% mass loss (0.015 kg lost to air per kg of hydrogen supplied to vehicles) is assumed with 99 % confidence[46]. The GWP characterization factors for H_2 is from the report[47].

Table S5 Characterization factors of global warming potential for different greenhouse gases

Greenhouse gas	GWP characterization factor	Remark
CO_2	1.00	1 kg CO_2 = 1.00 kg CO_2-eq.
CO	4.06	1 kg CO = 4.06 kg CO_2-eq.
N_2O	264.80	1 kg N_2O = 264.80 kg CO_2-eq.
H_2	11.00	1 kg H_2 = 11.00 kg CO_2-eq.

References

1. PBL. Integrated Model to Assess the Global Environment (IMAGE) 3.2. https://models.pbl.nl/image/index.php/Welcome_to_IMAGE_3.2_Documentation (2021).
2. Wolrd Bank. Population estimates and projections. https://databank.worldbank.org/source/population-estimates-and-projections# (2022).
3. OECD. Real GDP long-term forecast. https://data.oecd.org/gdp/real-gdp-long-term-forecast.htm (2022).
4. OECD. Purchasing power parities (PPP). https://data.oecd.org/conversion/purchasing-power-parities-ppp.htm (2022).
5. Dargay, J., Gately, D. & Sommer, M. Vehicle ownership and income growth, worldwide: 1960-2030. *Energy J.* **28**, 143–170 (2007).
6. IEA. *Global EV Outlook 2021: Accelerating ambitions despite the pandemic*. https://iea.blob.core.windows.net/assets/ed5f4484-f556-4110-8c5c-4ede8bcba637/GlobalEVOutlook2021.pdf (2021).
7. IEA. *World Energy Outlook 2021*. https://iea.blob.core.windows.net/assets/4ed140c1-c3f3-4fd9-acae-789a4e14a23c/WorldEnergyOutlook2021.pdf (2021).
8. IEA. *Net Zero by 2050-A Roadmap for the Global Energy Sector*. https://iea.blob.core.windows.net/assets/deebef5d-0c34-4539-9d0c-10b13d840027/NetZeroby2050-ARoadmapfortheGlobalEnergySector_CORR.pdf (2021).
9. IEA. *Energy technology perspective 2010: scenarios & strategies to 2050*. https://iea.blob.core.windows.net/assets/04776631-ea93-4fea-b56d-2db821bdad10/etp2010.pdf (2008).
10. ANL. *China vehicle fleet model: estimation of vehicle stocks,usage, emission, and energy use*. https://www.anl.gov/argonne-scientific-publications/pub/148761 (2018).
11. Hao, H. *et al.* Securing Platinum-Group Metals for Transport Low-Carbon Transition. *One Earth* **1**, 117–125 (2019).
12. ANL. *Modeling the Performance and Cost of Lithium-Ion Batteries for Electric-Drive Vehicles, Third Edition*. https://www.osti.gov/servlets/purl/1503280 (2019).
13. Xu, C. *et al.* Future material demand for automotive lithium-based batteries. *Commun. Mater.* **1**, 99 (2020).
14. Hao, H. *et al.* Impact of transport electrification on critical metal sustainability with a focus on the heavy-duty segment. *Nat. Commun.* **10**, 5398 (2019).
15. Miotti, M., Hofer, J. & Bauer, C. Integrated environmental and economic assessment of current and future fuel cell vehicles. *Int. J. Life Cycle Assess.* **22**, 94–110 (2017).
16. ANL. *The Greenhouse gases, Regulated Emissions, and Energy use in Technologies Model*. https://greet.es.anl.gov/index.php (2021) doi:10.11578/GREET-Excel-2021/dc.20210902.1.
17. Pederzoli, D. W. *et al.* Life cycle assessment of hydrogen-powered city buses in the High V.LO-City project: integrating vehicle operation and refuelling infrastructure. *SN Appl. Sci.* **4**, 57 (2022).
18. Ai, N., Zheng, J. & Chen, W. Q. U.S. end-of-life electric vehicle batteries: Dynamic inventory modeling and spatial analysis for regional solutions. *Resour. Conserv. Recycl.* **145**, 208–219 (2019).

19. Ziemann, S., Müller, D. B., Schebek, L. & Weil, M. Modeling the potential impact of lithium recycling from EV batteries on lithium demand: A dynamic MFA approach. *Resour. Conserv. Recycl.* **133**, 76–85 (2018).
20. Tao, Y., Rahn, C. D., Archer, L. A. & You, F. Second life and recycling: Energy and environmental sustainability perspectives for high-performance lithium-ion batteries. *Sci. Adv.* **7**, 1–17 (2021).
21. Neubauer, J., Smith, K., Wood, E. & Pesaran, A. *Identifying and Overcoming Critical Barriers to Widespread Second Use of PEV Batteries*. https://www.nrel.gov/publications. (2015).
22. ITF. Internaltional transport forum dataset. https://stats.oecd.org/Index.aspx?DataSetCode=ITF_GOODS_TRANSPORT (2021).
23. Eurostat. Goods transport by road. https://ec.europa.eu/eurostat/databrowser/view/ttr00005/default/table?lang=en (2022).
24. NationMaster. *Top countries in road freight transport*. https://www.nationmaster.com/nmx/ranking/road-freight-transport (2022).
25. Eurostat. Passenger road transport on national territory, by type of vehicles registered in the reporting country. https://ec.europa.eu/eurostat/databrowser/view/ROAD_PA_MOV/default/table?lang=en&category=road.road_pa (2021).
26. NationMaster. Top countries in road passenger transport. https://www.nationmaster.com/nmx/ranking/road-passenger-transport (2022).
27. IEA. *The future of trucks – Implications for energy and the environment(2nd edition)*. https://iea.blob.core.windows.net/assets/a4710daf-9cd2-4bdc-b5cf-5141bf9020d1/TheFutureofTrucksImplicationsforEnergyandtheEnvironment.pdf (2017).
28. ITF. *ITF Transprot Outlook 2019*. https://www.oecd-ilibrary.org/transport/itf-transport-outlook-2019_transp_outlook-en-2019-en (2019).
29. Hochfelder, B. What retailers can do to make the last mile more efficient. https://www.supplychaindive.com/news/last-mile-spotlight-retail-costs-fulfillment/443094/ (2017).
30. Deloitte. Last mile delivery landscape in the transportation sector. (2022).
31. Steubing, B., de Koning, D., Haas, A. & Mutel, C. L. The Activity Browser — An open source LCA software building on top of the brightway framework. *Softw. Impacts* **3**, 100012 (2020).
32. Sacchi, R. *et al.* PRospective EnvironMental Impact asSEment (premise): A streamlined approach to producing databases for prospective life cycle assessment using integrated assessment models. *Renew. Sustain. Energy Rev.* **160**, 112311 (2022).
33. World Bank. *Mineral for climate action: The mineral intensity of the clean energy transition*. https://pubdocs.worldbank.org/en/961711588875536384/pdf/Minerals-for-Climate-Action-The-Mineral-Intensity-of-the-Clean-Energy-Transition.pdf (2020).
34. Weil, M. & Ziemann, S. Recycling of Traction Batteries as a Challenge and Chance for Future Lithium Availability. in *Lithium-Ion Batteries* 509–528 (Elsevier, 2014). doi:10.1016/B978-0-444-59513-3.00022-4.
35. EUI. *International Historical Statistics*. (Palgrave Macmillan UK, 2013). doi:10.1057/9781137305688.

36. NationMaster. Vehicles in Use. https://www.nationmaster.com/nmx/timeseries/united-states-vehicles-in-use (2022).
37. Pauliuk, S. & Heeren, N. ODYM—An open software framework for studying dynamic material systems: Principles, implementation, and data structures. *J. Ind. Ecol.* **24**, 446–458 (2020).
38. IPCC. *Climate Change 2014: Mitigation of Climate Change. Contribution of Working Group III to the Fifth Assessment Report of the Intergovernmental Panel on Climate Change*. https://www.ipcc.ch/site/assets/uploads/2018/02/ipcc_wg3_ar5_chapter8.pdf (2014).
39. USGS. *Mineral commodity summaries 2021*. https://pubs.usgs.gov/periodicals/mcs2021/mcs2021.pdf (2021).
40. Statista. Distribution of cobalt demand worldwide from 2010 to 2025, by end use. https://www.statista.com/statistics/875803/cobalt-demand-share-end-use-worldwide/ (2022).
41. Statista. Distribution of lithium end-usage worldwide in 2021, by area of application. https://www.statista.com/statistics/268787/lithium-usage-in-the-world-market/ (2022).
42. Nickel Institute. About nickel. https://nickelinstitute.org/en/about-nickel-and-its-applications/ (2022).
43. CMP Group. Platinum Industrial Demand. https://www.cmegroup.com/education/articles-and-reports/platinum-industrial-demand.html (2020).
44. EEA. *Air pollutant emission inventory guidebook 2019*. https://www.eea.europa.eu/publications/emep-eea-guidebook-2019/part-b-sectoral-guidance-chapters/1-energy/1-a-combustion/1-a-3-b-i/view (2019).
45. IPCC. *IPCC fifth assessment report*. https://www.ipcc.ch/assessment-report/ar5/ (2013).
46. Consultancy, F.-N. *Fugitive Hydrogen Emissions in a Future Hydrogen Economy*. https://assets.publishing.service.gov.uk/government/uploads/system/uploads/attachment_data/file/1067137/fugitive-hydrogen-emissions-future-hydrogen-economy.pdf (2022).
47. Warwick, N. *et al. Atmospheric implications of increased Hydrogen use*. https://assets.publishing.service.gov.uk/government/uploads/system/uploads/attachment_data/file/1067144/atmospheric-implications-of-increased-hydrogen-use.pdf.
